# ACTIVATE: A protocol for a randomised controlled evaluation of Thumos: A cognitive-behavioural intervention for medical students

**DOI:** 10.1371/journal.pone.0355371

**Published:** 2026-09-25

**Authors:** Lucy Pointon, William Lea, Christopher D. J. Taylor, Luke Budworth, Dialechti Tsimpida, Rebecca Coleman, Nathan Shearman, Reema Harrison, Vijay Jayagopal, Gursharan Johal, Katharina Sophie Vogt, Raoul Ovalekar, Rajjan Singh, Nathan Randles, Jamie Read, Faye Gishen, John Hines, Justin Ward, Sam Thenabadu, Toby Helliwell, Joanne Ogundiran, Judith Johnson

**Affiliations:** 1 School of Health Sciences, University of Manchester, Manchester, United Kingdom; 2 York and Scarborough Teaching Hospitals NHS Foundation Trust, York, United Kingdom; 3 Hull York Medical School, York, United Kingdom; 4 School of Psychology, University of Sheffield, Sheffield, United Kingdom; 5 Pennine Care NHS Foundation Trust, Ashton Under Lyne, United Kingdom; 6 School of Psychology, University of Leeds, Leeds, United Kingdom; 7 Division of Public Health and Epidemiology, School of Medical Sciences, College of Life Sciences University of Leicester, Leicester, United Kingdom; 8 College of Medical, Veterinary and Life Sciences, University of Glasgow, Glasgow, United Kingdom; 9 Independent Practice, Sheffield, United Kingdom; 10 Faculty of Medicine, Health and Human Sciences, Macquarie University, Sydney, Australia; 11 Department of Psychology, University of Liverpool, Liverpool, United Kingdom; 12 School of Medical Sciences, University of Manchester, Manchester, United Kingdom; 13 School of Medicine, Keele University, Keele, United Kingdom; 14 Lincoln Medical School, University of Lincoln, Lincoln, United Kingdom; 15 UCL Medical School, Faculty of Medical Sciences, University College London, London, United Kingdom; 16 GKT School of Medicine, King’s College London, London, United Kingdom; 17 Midlands Partnership University NHS Foundation Trust, Stafford, United Kingdom; 18 Faculty of Medicine, University of Leeds, Leeds, United Kingdom; 19 Manchester University NHS Foundation Trust, Manchester, United Kingdom; King Abdulaziz University Faculty of Medicine, SAUDI ARABIA

## Abstract

**Background:**

Medical students experience high rates of burnout and mental health distress, with approximately half reporting burnout, one-third experiencing depression, and one-tenth having suicidal thoughts. These issues are exacerbated during clinical placements and contribute to workforce attrition after qualification. Existing interventions are largely generic and show limited effectiveness. Thumos, a cognitive-behavioural therapy-based intervention specifically tailored for healthcare professionals and medical students, addresses the stressful situations intrinsic to medical training and practice. This randomised controlled trial will evaluate whether Thumos improves psychological resilience, confidence in dealing with stressful situations, burnout, and depression in medical students compared with services as usual (SAU).

**Methods/design:**

Two hundred and twenty UK medical students in a year that contains a clinical placement of a week or longer will be recruited nationally from UK medical schools. Participants will be randomised 1:1 to receive either Thumos (two online group workshops and one individual video/phone call) or SAU. Assessments will occur at baseline, post-intervention, 4-month, and 9-month follow-up. The primary outcome is psychological resilience measured by the Brief Resilience Scale at 4-month follow-up. A mixed-methods process evaluation will assess acceptability and feasibility.

**Discussion:**

To our knowledge, this is the first randomised controlled trial of an intervention specifically designed to prepare medical students for stressful clinical events. If effective, Thumos could potentially be adapted for medical students in other countries.

**Trial identifier and registry name:**

ISRCTN12744098: https://www.isrctn.com/ISRCTN12744098; An evaluation of a cognitive-behavioural intervention for medical students

## Introduction

Healthcare systems internationally are experiencing a workforce crisis, with a current global shortage of six million physicians [1]. While poor workforce planning has contributed to this problem, high turnover has been identified as the main contributing factor [2]. Inadequate staffing is linked with negative patient outcomes including extended hospital stays and mortality [3]. It also creates a vicious cycle, whereby existing staff become overworked and burnt-out, a syndrome characterised by exhaustion and disengagement [4]. Higher burnout then increases the risk of sickness absence and turnover, further exacerbating staffing shortages [5,6] and directly leading to the delivery of poorer patient care [7].

In a review of 170 studies in doctors, higher burnout was associated with twice the risk of patient safety incidents and a two-fold reduction in patient satisfaction [7]. Within this vicious cycle, provider burnout and poor well-being must be recognised as an active component, serving to drive the workforce crisis. Equally, addressing this component by better supporting healthcare professionals could help to break this cycle, leading to reduced sickness absence, better retention, and better patient care [8].

Most burnout research has focused on qualified professional groups, but there is a growing recognition that it could be beneficial to intervene during training, prior to professional registration, due to high rates of mental health distress in these cohorts [9]. Studies in medical students indicate half report burnout, one in three report depression and one in ten experience suicidal thoughts [10–12]. Furthermore, the immediate post-qualification years have been recognised as a high-risk time for physicians leaving the profession [13]. Poor mental health and burnout are linked with higher attrition [14], so improving their mental well-being could be one way to enhance retention and reduce physician shortages.

Multiple risk factors unique to medical training have been implicated in these mental health difficulties, notably demanding academic requirements and perfectionism-related cognitive patterns [15,16] and exposure to stressful clinical events on placement [17]. Indeed, rates of burnout in medical students increase significantly during the years involving clinical placements [18]. However, there has been a lack of interventions tailored for medical students and none which prepare medical students for stressful workplace events [19]. Previous evaluations of mental health support for medical students have predominantly examined non-tailored approaches such as mindfulness-based programmes, relaxation techniques, and yoga [19]. Evidence for these interventions is weak and mixed, and they have been criticised for lacking relevance [20]. A recent meta-analysis of resilience interventions for medical students indicated that these interventions are moderately effective at reducing stress and mildly effective at increasing resilience within medical students [21]. The implication that these interventions may have more favourable impacts on stress suggests that further research is required to examine the specific factors that influence resilience. There is an urgent need for effective, preventative interventions which help students cope with the specific occupational stressors they will encounter during their placements, training, and beyond.

The current trial will evaluate Thumos, an intervention that employs cognitive-behavioural therapy (CBT) techniques to prepare healthcare professionals and students to manage stressful work or training events. Thumos is based on the principles of Reboot (Recovery-boosting), an approach that has been investigated, tested and developed in several studies previously [9,22–26]. The approach used in Thumos differs from many previously evaluated psychological interventions for medical students in three ways. Firstly, it has been designed with healthcare professionals in mind, based upon a combination of 1) research into the effects of patient safety incidents and other work stressors, 2) resilience theory, and 3) CBT techniques. Secondly, Thumos integrates complementary therapeutic formats: group sessions that facilitate peer connection, alongside an individual consultation that provides personalised support. Thirdly, Thumos addresses the stressful situations that are intrinsic to healthcare work, providing support for those unavoidable events that cannot be improved through organisational interventions.

Characteristics of Thumos may make it particularly well suited for medical students. For example, the preventive framing of the intervention may help address barriers related to help-seeking stigma, which research has identified as a significant obstacle to engagement with mental health support among medical students [20,27]. It also uses case studies and materials which are tailored towards the stressful experiences medical students may face on placements. This approach increases the sense of relevance of the intervention for participants which may increase engagement [9]. Furthermore, it directly addresses unhelpful perfectionist thinking styles that have been associated with psychological distress in this population [16].

There have been three uncontrolled evaluations of versions of Reboot, the CBT-based intervention on whose principles Thumos is based, which indicate positive findings. The first was in a group of 66 multi-disciplinary healthcare professionals and students; results indicated it was associated with improvements in participants’ confidence, coping knowledge and psychological resilience [22,23]. The second was in 80 Critical Care Nurses (CCNs) [24,25]. Participation was associated with increased confidence and resilience and reduced depression and burnout. Nurses also reported a lower intention to leave clinical practice following the intervention [24]. The third evaluation was in 115 medical students [9]. Findings indicated the intervention was feasible and potentially effective in this group, with participants reporting significant increases in resilience and confidence in coping with adverse events, and significant decreases in burnout and depression [9]. Qualitative findings from these evaluations indicate that participants appreciate the intervention’s tailored format and opportunity for peer support alongside professional input. They describe increased self-awareness and report the acquisition of psychological skills which they could apply to cope with work stressors [22–26].

Feedback from these evaluations was used to make improvements to, and to further tailor, Reboot towards medical students, including new exercises, reduction of didactic information and changing the phone call discussion. There is now a need to test this refined intervention, Thumos, and to ascertain whether observed improvements in outcome indicators can be attributed to the intervention. This study addresses this gap, through a definitive randomised controlled trial (RCT). The comparator intervention is Services as Usual (SAU); this comparison will establish whether and what benefits Thumos may offer above what is currently available. The final follow-up time point is 9 months and create the possibility of conducting a subsequent 5-year follow-up study, should initial results be positive.

## Aims and objectives

The main purpose of the trial is to establish whether Thumos increases resilience and improves mental health in medical students.

The primary objective is:

- To determine whether Thumos is superior to the control condition (services as usual; SAU) at 4-month follow-up in increasing psychological resilience in medical students.

Secondary objectives are:

1. To determine whether Thumos is superior to SAU at 9-month follow-up in increasing psychological resilience.
2. To determine whether Thumos is superior to SAU at 4-month and 9-month follow-ups in the endpoints of confidence, burnout, depression, mental health, and self-reported sickness absence.
3. To assess acceptability and how Thumos is delivered in practice through a process evaluation with users, medical educators, and providers

## Materials and Methods

### Trial design

**Fig 1:**
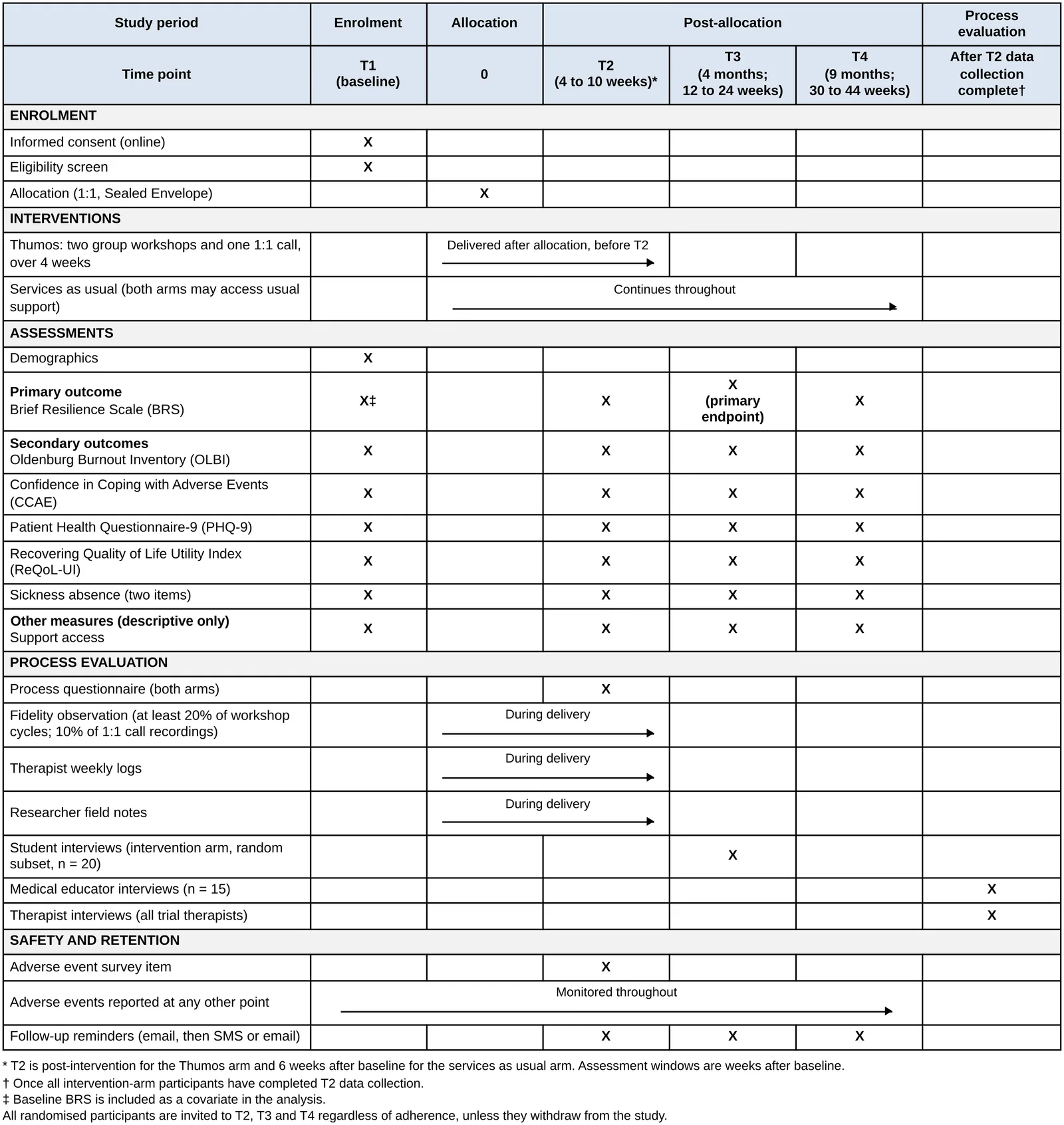
SPIRIT schedule of enrolment, interventions, and assessments.

This study is a randomised controlled parallel groups intervention trial (see S1 CONSORT Checklist in S1 File). All United Kingdom (UK) medical schools will be invited to participate (see S3 Appendix 1 in S3 File). Participants will be self-selecting and screened for eligibility based on the criteria that they are a) UK medical student and b) in a year of study that contains a placement of one week or longer. The trial will compare Thumos to a control condition of services as usual (SAU), where medical students in either condition can continue to access available mental health support as they usually would. Data collection will occur at four time-points, Time 1 (T1), pre-intervention (baseline); Time 2 (T2), post-intervention (following completion of Thumos/6 weeks after baseline for control group); Time 3 (T3), 4 months following baseline and Time 4 (T4), 9 month following baseline (see Fig 1). All elements will be conducted online, with questionnaires administered via Qualtrics and interviews/intervention sessions via online video platform. Participants will be contacted to complete the post-baseline measures via Qualtrics using email as the first point of contact and subsequent attempts to contact will be via SMS or email. All participants that complete post-baseline measures will be awarded a £10 shopping voucher per survey as compensation. Informed consent will also be taken online, with participants asked to read the information sheet and provide written informed consent via Qualtrics prior to responding to any data collection items (see S4 Appendix 2 in S4 File). A process evaluation will include medical student participants, medical educators from medical schools of participating students and the Thumos therapists.

### Randomisation and Blinding

Randomisation will be undertaken following completion of baseline assessments using the web-based Sealed Envelope software. Randomisation will be in the ratio 1:1 to the two groups. *The statistician will conduct the primary analysis using masked treatment labels, with allocation revealed only after the primary dataset is locked and the masked primary results have been checked, as described under Statistical Analysis*. The CBT therapists, researchers and participants will be unblinded.

### Study population for randomised controlled trial

#### Inclusion criteria.

UK medical students in years involving clinical placements of a week or longer nationally. We will recruit from these groups to the main trial until recruitment numbers are saturated.

### Exclusion criteria

Medical students based in a medical school outside of the UK or students in a year without a clinical placement of a week or longer. Recruitment will end once we have reached our maximum number of participants to recruit (n = 220) and exclude all prospective participants at this time.

### Study population for process evaluation

#### Inclusion criteria.

Participating medical students: We will recruit medical students in the main trial to also take part in the process evaluation. Both control and intervention arm students will be invited to complete process questionnaires; only intervention arm medical students will be invited to take part in qualitative interviews.

UK medical educators: We will recruit medical educators in schools with participating students within the intervention arm to qualitative interviews as part of the process evaluation.

The cognitive behavioural therapists/clinical psychologists delivering the intervention will be invited to qualitative interviews as part of the process evaluation.

### Exclusion criteria

Medical students: We will exclude those who did not complete the intervention from the qualitative interviews.

UK Medical educators: We will exclude those from medical schools without participating intervention arm students. For the qualitative interviews, we will stop recruiting once we have reached data saturation, defined as ‘information redundancy’ where no new information or value is generated from the data.

### Recruitment Methods

Medical student participants will be recruited via the Medical Protection Society (emails to medical student members), the Medical Schools Council (email cascade to UK medical schools), direct email communications to heads of UK medical school and via posts on social media (LinkedIn, BlueSky, Twitter, Instagram). Participants will then be directed to a Qualtrics survey online where they will have access to the participant information sheet before being asked to complete the online consent form and baseline measures.

For the process evaluation, medical educators in schools with participating students who were allocated to the intervention arm will be contacted via emails sent to heads of medical schools with participating students. Trial therapists will be contacted directly via email.

Regarding the recruitment period, the recruitment of medical students commenced on 16^th^ June 2025 and is expected to end on the 31^st^ March 2026. Following this, the recruitment of Medical Educators and trial therapists for the process evaluation is scheduled to commence July 2026 and is expected to be completed by no later than October 2026.

### Intervention

#### Participants randomised to THUMOS (Experimental group).

Thumos is a brief, manualised cognitive-behavioural coaching programme developed specifically for medical students, involving case studies of stressful events medical students may experience on placements. It is based upon the principles of the Reboot (Recovery-boosting) coaching programme, an earlier CBT-based resilience intervention for healthcare professionals and trainees [9,22–26]. Thumos is preventative in focus, equipping students with cognitive and behavioural strategies before, rather than only in response to, the stressful events intrinsic to clinical training. It is grounded in cognitive-behavioural therapy (CBT), in which thoughts, emotions, physical sensations and behaviours are understood as interconnected, such that modifying unhelpful thinking and behaviour can improve emotional wellbeing [28,29]. It also draws on resilience theory [30]. The strategies taught are cognitive (for example, challenging negative automatic thoughts and reattributing responsibility), behavioural (for example, mood-boosting activities, problem-solving and breathing exercises) and meta-cognitive (worry postponement and gratitude). The full intervention manual is available from the authors on request.

The programme runs over four weeks and comprises two online group workshops (120 minutes each, in stable groups typically of around four to eight) and one individual coaching call (60 minutes), with an intervening practice week. All sessions are delivered remotely by qualified therapists (HCPC-registered Clinical Psychologists or BABCP-accredited CBT therapists) trained in the Thumos protocol, and participants receive a workbook in advance.

**Workshop 1** addresses the CBT model, identifying and challenging negative automatic thoughts, common unhelpful thinking habits (for example, catastrophising and mind-reading), and the role of beliefs and self-esteem. Individual exercises include mapping a personal placement stressor onto the CBT model (identifying the most distressing “hot thought”) and completing a thought record. Group-based activities include a facilitator-led formulation of a shared example and discussion of a medical-student case study to spot thinking habits.

**Workshop 2** addresses the physiological stress response and breathing exercises, mood-boosting activities, reducing self-blame through balanced appraisals of responsibility, and a five-step problem-solving activity. Individual exercises include a guided breathing exercise, a personal mood-boosting plan, and a “responsibility pie chart”. Group-based, breakout-room activities use medical-student case studies to distinguish helpful from unhelpful behaviours and to practise responsibility appraisal and problem-solving.

**The individual coaching call** personalises the content: the facilitator reviews the strategies tried, identifies “stuck points”, and chooses from a selection of CBT-based intervention strategies (e.g., self-statements and thought challenging, gratitude, distraction, postponing worry, breathing exercises) using a decision tree, before co-producing a personal “blueprint” of takeaways and support sources.

To ensure the quality and fidelity of the Thumos intervention throughout the trial, a structured fidelity monitoring protocol is in place whereby researchers observe a minimum of 20% of workshop cycles and review recordings of 10% of individual video/phone calls, using documented fidelity assessment criteria. Therapists complete workshop diaries capturing their experiences of intervention delivery, including perceived benefits and challenges, and researcher field notes track any deviations from the protocol in real time. Participant adherence is defined as completion of both group workshops with at least 75% attendance at each session, and those who miss sessions are proactively contacted by email, text, and telephone to support engagement. The quality of qualitative process data is further strengthened through credibility strategies including respondent validation of transcripts, peer debriefing, and negative case analysis.

### Participants Randomised to Control Group

**Fig 2:**
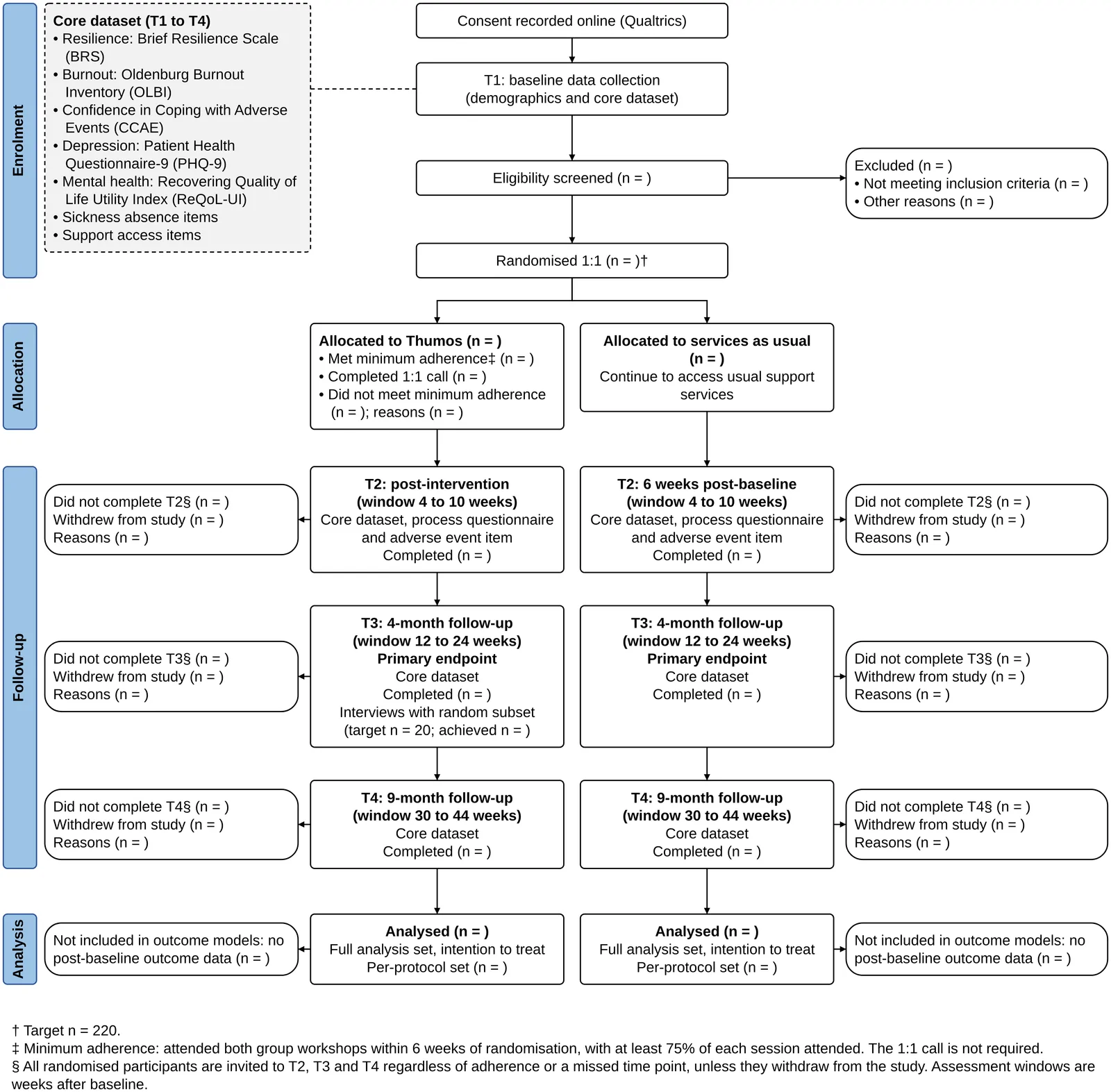
Flow diagram of participants through the trial.

Control condition participants will not receive an alternative intervention. However, they can continue to access mental health services that are usually available via medical schools, universities or to the public throughout the period that they are participating. A flow diagram of the participants journey through the trial can be seen in Fig 2.

### Data Collection and Outcome Measures

A core online questionnaire set including measures of resilience (Brief Resilience Scale [31]), burnout (Oldenburg Burnout Inventory; OLBI [4]) confidence in coping with adverse events (CCAE [9]) depression (Patient Health Questionnaire, PHQ-9 [32]), mental health (Recovering Quality of Life-Utility Index, ReQoL-UI [33]) sickness absence and support access will be completed at T1, T2, T3 and T4 (see Tables 1 and 2). In addition, at T2 a process questionnaire will be added to this dataset (see Table 3).

**Table 1:** Outcome measures.

Outcome	Time	Variable definition	Effect measure
BRS (primary)	T3	BRS mean score	Adjusted mean difference/Cohen’s *d*
BRS (secondary)	T4	BRS mean score	Adjusted mean difference/Cohen’s *d*
CCAE	T3/T4	CCAE mean score	As above
OLBI	T3/T4	OLBI total and subscales	As above
PHQ-9	T3/T4	PHQ-9 total score	As above
ReQoL-UI	T3/T4	Utility score	As above
ReQoL-UI QALYs	BL-T4	QALYs area under the curve	Adjusted mean difference
Sickness absence	T3/T4	Days lost in past 4 weeks	Adjusted rate ratios with 95% CI

BRS: Brief Resilience Scale; CCAE: Confidence in Coping with Adverse Events; OLBI: Oldenburg Burnout Inventory; PHQ-9: Patient Health Questionnaire-9 item depression scale; ReQoL-UI: Recovering Quality of Life – Utility Index; QALYs: Quality-adjusted life years; BL: Baseline; T1, T2, T3, T4: Time points 1, 2, 3, and 4 (T = time)

**Table 2:** Outcome scoring.

Outcome	Items	Measure	Construct(s) & direction	Time points	Derived variable/scoring (incl. item response scale)
BRS (Primary)	6	Brief Resilience Scale	Resilience; higher = better	T3 [12–24 wks]	Mean of the six items after reverse scoring items 2, 4 and 6. Each item rated on a 5-point scale: 1 (Strongly disagree), 2 (Disagree), 3 (Neutral), 4 (Agree), 5 (Strongly agree).
BRS	6	Brief Resilience Scale	Resilience; higher = better	T4 [30–44 wks]	Mean of the six items after reverse scoring items 2, 4 and 6; each item rated 1 (Strongly disagree) to 5 (Strongly agree), with a “Neutral” midpoint.
CCAE	3	Confidence in Coping with Adverse Events questionnaire	Confidence coping with adverse events; higher = better	T3, T4 [12 –24, 30–44]	Mean of 3 items. Each item rated on a 4-point scale: “No – not at all”, “Unsure”, “Yes – to some extent”, “Yes – definitely”.
OLBI (total & subscales)	16	Oldenburg Burnout Inventory	Burnout (exhaustion, disengagement); higher = worse	T3, T4 [12 –24, 30–44]	Subscale means and total mean, with reverse scoring of positively worded items according to the published scoring instructions. Each item rated on a 4-point scale: Strongly disagree, Disagree, Agree, Strongly agree.
PHQ-9	9	Patient Health Questionnaire-9	Depressive symptoms; higher = worse	T3, T4 [12 –24, 30–44]	Total score obtained by summing the nine items (range 0–27). Each item rated over the past two weeks: 0 (Not at all), 1 (Several days), 2 (More than half the days), 3 (Nearly every day).
ReQoL-UI (utility)	10	Recovering Quality of Life – Utility Index (ReQoL-UI)	Quality of life utility; higher = better	T3, T4 [12 –24, 30–44]	Utility index derived via the published ReQoL-UI algorithm, which combines the 10 mental-health items with the single physical-health item. Each mental-health item is rated on a 5-point scale from “None of the time” to “Most or all of the time” and coded 0–4 before the algorithm is applied; the physical-health item is rated from “No problems” to “Severe problems”.
ReQoL-UI QALYs	10	Recovering Quality of Life – Utility Index (ReQoL-UI)	Quality adjusted life years; higher = better	Baseline–T4	Area under the utility-time curve (derived from the utility scores above; no separate item response scale).
Sickness absence	2	Sickness absence items	Days lost last 4 weeks; higher = worse	T3, T4 [12 –24, 30–44]	Count of days lost in past 4 weeks (free numeric response).

BRS: Brief Resilience Scale; CCAE: Confidence in Coping with Adverse Events; OLBI: Oldenburg Burnout Inventory; PHQ-9: Patient Health Questionnaire-9 item depression scale; ReQoL-UI: Recovering Quality of Life – Utility Index; QALYs: Quality-adjusted life years; T3, T4: follow-up time points.

**Table 3:** Questionnaire assessment schedule.

	Baseline	T2 (post intervention or equivalent time)	T3 (4 month follow up)	T4 (9 month follow up)
**Enrolment**				
Informed consent form	X	–	–	–
**Primary outcome**				
Brief Resilience Scale (BRS)	X	X	X	X
**Secondary outcomes**				
Oldenburg Burnout Inventory (OLBI	X	X	X	X
Confidence in coping with Adverse Events (CCAE) questionnaire	X	X	X	X
Patient-Health Questionnaire-9 (PHQ-9)	X	X	X	X
Recovering Quality of Life-Utility Index (ReQoL-UI)	X	X	X	X
Self-reported sickness absence	X	X	X	X
Other measures (descriptive only)				
Support access	X	X	X	X
**Process evaluation**				
Process questionnaires	–	X	–	–

#### Primary outcome.

**To determine whether Thumos is superior to SAU at a 4-month follow-up in increasing psychological resilience.**

- Brief Resilience Scale (BRS): The 6-item BRS measures perceptions of personal resilience, including ‘I tend to bounce back quickly after hard times’. This questionnaire has good internal reliability (α = .80−.91), concurrent validity with other resilience questionnaires [31], and detects learning changes [9].

#### Secondary outcomes.

**To determine whether Thumos is superior to SAU at 4-month and 9-month follow-ups in the endpoints of confidence, burnout, depression, mental health, and self-reported sickness absence.**

- Oldenburg Burnout Inventory (OLBI). The scale comprises 16 items and two subscales (exhaustion and disengagement). It has been used widely and found to have good internal reliability (α = .88) [4].
- Confidence in coping with Adverse Events (CCAE) questionnaire: This 3-item questionnaire assesses how confident participants are in coping with adverse events, e.g., ‘If I was involved in an adverse event for which I thought I held some responsibility I know the things I would do to help manage my stress levels’. Previous studies suggest this scale has good internal reliability (α = 0.85) [26] and detects learning changes [9].
- Patient-Health Questionnaire-9 (PHQ-9). This 9-item questionnaire is widely used to measure depression and has been validated in various groups, including medical students [34]. It has good internal consistency (α = .83) and captures fluctuations [35].
- Recovering Quality of Life-Utility Index (ReQoL-UI) [33]. Mental health will be measured with the 10-item ReQoL-UI. This validated, reliable questionnaire measures mental health across areas of quality of life. It applies to the full spectrum of mental health conditions and provides Quality of Life Adjusted Years (QALY) estimates.
- Self-reported sickness absence. Two items will be used to measure sickness absence, the first which asks about total days lost within the past 4 weeks; the second of which asks how many working or studying days specifically were lost within this timeframe.

Other Measures:

- Support access. Four items will be used to assess whether participants are accessing any form of psychological wellbeing service, which type of support they are accessing, when they began accessing this, and when they anticipate, they will stop accessing this. These data will be collected at baseline, T2, T3 and T4 and summarised descriptively by arm and time point to describe support received alongside trial participation. Support access is not a secondary efficacy outcome and will not be analysed using models or used as a covariate.

### Process evaluation outcomes

**To assess acceptability and how Thumos is delivered in practice through a process evaluation with users, medical educators, and providers.**

We drew upon UK Medical Research Council (MRC) guidance for process evaluations of complex interventions [36] to design a mixed-methods approach to the process evaluation.

- Psychological therapist logs: Weekly logs will assess therapists’ experiences of intervention delivery, including perceived benefits, mechanisms, and challenges.
- Process questionnaires: Process questionnaires at T2 (post-intervention) will explore participants’ reasons for participating, and their perceptions and experiences [37]. Participants in the intervention arm will be asked about their affective attitudes to the intervention, general acceptability, perceived effectiveness, ethicality, intervention coherence and mechanisms, self-efficacy, burdens, opportunity costs, and barriers and facilitators. Participants in the control arm will be asked about their reasons for participating, and their perceptions and experiences of the study.
- Field notes: Researchers’ field notes will track any changes in the delivery of the intervention; enabling recording of protocol changes.
- Participant interviews: At T3, a randomly selected subset of participants from the intervention arm will be invited to 30-minute semi-structured interviews (n = 20) to generate information regarding intervention delivery, feasibility and acceptance, and barriers and facilitators to participation.
- Medical educator interviews: Once all experimental participants have completed T2 data collection, medical educators (n = 15) at schools where students have participated in the study will be interviewed to explore 1) their perceptions of the research process, and 2) perceived benefits or drawbacks of Thumos at an organisational level, 3) ways Thumos could be implemented at scale for medical students.
- Therapist interviews: Once all experimental participants have completed T2 data collection, all trial therapists will be invited to 30-minute semi-structured interviews exploring their experiences of delivering the intervention.

### Safety Reporting

In this study, an adverse event (AE) will be considered any untoward medical occurrence occurring in an ACTIVATE participant whether or not related to the research procedures or intervention. An adverse reaction (AR) will be defined as an untoward and unintended response in a participant that is potentially connected to any aspect of ACTIVATE. A Serious adverse event (SAE) is defined as any adverse event that is serious and is life‑threatening or may lead to death, hospitalisation, disability, or other major medical events. A serious adverse reaction (SAR) is defined as a serious adverse event (SAE) that is related to the study. Adverse events will be recorded and reviewed by the CI, and summary reports provided to the Trial Steering committee (TSC) for review of any possible patterns.

AEs will be monitored and recorded at T2 via the inclusion of a survey question. We will also monitor them via items on the process questionnaires relating to moral and ethical concerns about the study, affective attitude towards the study (like/dislike) and experience of the study (degree of comfort experienced). All participants who indicate they may have experienced an adverse event via the survey question or report significant moral or ethical concerns, strongly disliking the study or feeling very uncomfortable will be contacted for further information and to determine whether the incident can be classed as an AE, AR, SAE or SAR.

If participants indicate during any other part of the study (e.g., during the workshops, the one-to-one call, interviews, or by contacting the study team) that an AE or SAE has occurred, they will similarly be contacted by the study team and asked to provide more information.

An SAE occurring to a research participant will be reported to the Chair of the Ethics Committee where in the opinion of the Chair of TSC that the event was: i) related, that is resulted from the administration of any of the research procedures and ii) unexpected – that is, the type of event is not listed in the protocol as an expected occurrence. The CI and the trial management team will review all SAEs, whether or not they are judged to be attributable to the trial or interventions. Based on this information, and in consultation with the Chair of the TSC, they will determine whether the participant should be withdrawn, and/or the trial should be suspended, stopped or continued. All AEs, ARs, SAEs and SARs will be fully documented and included in both the final trial report and published journal article.

### Data Management

All data will be kept secure and maintained in accordance with the requirements of the Data Protection Act and archived according to clinical trial GCP regulations. Personal data will be stored in encrypted, password protected formats. Identifiable personal data will be stored separately to other study data to help maintain confidentiality. Only the University of Manchester research team members will have access to the data. Only anonymised datasets will be shared with collaborators outside of the University of Manchester for the purposes of analysis.

The full dataset will be retained for up to 5 years in first instance, to enable possible follow-up studies to be conducted, should additional funding be gained. It will then be deleted, and only anonymised datasets will be retained and uploaded to the University of Manchester data archive/repository. Consent forms will also be retained during this time and destroyed at the end of this time.

### Sample size calculation

Simulations (using *powerlmm*) gave approximately 80% power with 200 participants to detect *d* = 0.50 in BRS at four months (T3; two-sided α = .05). They included repeated measurements, participant intraclass correlations of 0.30-0.50 and dropout by T4 of 30% in Thumos and 40% in services-as-usual. Adding 10% for uncertainty in these assumptions yielded a final target of N = 220 participants randomised 1:1 to Thumos or services‑as‑usual.

### Statistical Analysis

Analyses will use R and follow the statistical analysis plan (SAP; S2 File). Participants will be analysed as randomised, whatever intervention they receive. Those without follow-up outcomes will appear in flow and baseline summaries but not the primary outcome model. Treatment labels will remain masked until the primary dataset is locked and masked primary results are checked.

Recruitment, retention and adherence will be summarised as counts and percentages. Minimum adherence requires both workshops within six weeks of randomisation, with at least 75% attendance at each; the individual call is not required.

Baseline characteristics will be summarised by arm using means and standard deviations, medians and interquartile ranges, or counts and percentages, as appropriate. Absolute standardised differences above 0.20 will be highlighted, without significance testing or changes to prespecified adjustment covariates.

The primary outcome is BRS at four months (T3). A repeated measures linear mixed model will include BRS at T2–T4, with fixed effects for arm, categorical time, their interaction, baseline BRS, age, gender and medical school. We will use restricted maximum likelihood estimation and Kenward–Roger tests and confidence intervals. Within-participant covariance will be unstructured, or first-order autoregressive if the model fails to converge. The primary comparison is the adjusted T3 mean difference, Thumos minus services as usual, tested two-sided at α = .05. We will report adjusted arm means, mean differences and Cohen’s *d (using the pooled baseline standard deviation),* with 95% confidence intervals.

Continuous secondary outcomes will use the same approach, adjusted for each outcome’s baseline value. The two sickness absence counts will use separate Poisson mixed models with a log link and participant random intercept, or negative binomial models if overdispersed. We will report adjusted incidence rate ratios, marginal mean days lost by arm and absolute differences, with 95% confidence intervals. QALYs from baseline to T4 will be calculated as the area under the ReQoL-UI utility curve using linear interpolation. They will be compared using linear regression adjusted for baseline utility, age, gender and medical school, within multiple imputation.

Support access will be described by arm and time, not treated as an outcome or covariate. Therapy group and therapist clustering will not be modelled. Secondary and exploratory findings will be supportive, not confirmatory. We will report unadjusted and Benjamini-Hochberg false discovery rate adjusted *p* values within separate T3 and T4 secondary outcome families [38], including BRS only at T4.

Mixed models will use available outcomes, assuming data are missing at random (MAR) given observed outcomes and covariates. We will summarise missingness patterns, response rates and stated reasons for non-response, and assess predictors using logistic regression.

Sensitivity analyses will include multiple imputation under MAR, pooled using Rubin’s rules; pattern mixture adjustments subtracting 0.10, 0.20 or 0.30 points from imputed T3/T4 BRS values in Thumos only; and carrying forward the latest observed value, using baseline if no follow-up value exists. Per protocol analyses will exclude major protocol deviations and non-adherent Thumos participants and require valid T3 BRS within 12–24 weeks of baseline.

A supplementary complier average causal effect analysis of T3 BRS will use a single instrumental variables model fitted by two-stage least squares, with allocation as the instrument for minimum adherence. Both stages will adjust for baseline BRS, age, gender and medical school. All randomised participants will be included, using multiple imputation for missing outcomes. We will report the effect and 95% confidence interval, Thumos adherence proportion and first-stage partial *F* statistic.

Residual and influence diagnostics will check model assumptions. Severe non-normality or unequal residual variances will prompt sensitivity analysis using robust standard errors clustered by participant. Robustness will be judged by consistency in the T3 effect’s direction and magnitude. Scale reliability will be reported as Cronbach’s alpha and McDonald’s omega with 95% bootstrap confidence intervals. Adverse and serious adverse events will be summarised by arm, with risk differences and 95% confidence intervals but no hypothesis tests.

Interviews conducted as part of the process evaluation will be transcribed verbatim and analysed using Reflexive Thematic Analysis (RTA). Field notes and logs will also be analysed using RTA. RTA is a theoretically flexible approach for identifying, analysing, and reporting patterns in qualitative data and is useful for obtaining detailed insights [39]. Internal reliability of qualitative data will be strengthened through the adoption of qualitative credibility strategies such as respondent validation of transcripts, peer debriefing, and negative case analysis. Qualitative data will then be combined with quantitative data from the process questionnaires using a narrative discussion where the quantitative survey scores are presented alongside the themes produced by RTA [40]. In particular, we will follow the approach of Guetterman and Mitchell [41] who included a separate section following initial presentation of the quantitative (frequency) and qualitative (thematic) findings, labelled, ‘mixed-methods integration’. The purpose of this section will be to bring quantitative results together with relevant qualitative insights, to generate a nuanced and full picture of the dataset. This narrative discussion will be facilitated by the use of a side-by-side joint data display; a table where complementary and divergent qualitative and quantitative findings are included in columns to support the generation of complex insights [42].

### Ethics

The trial has been approved by the University of Manchester Research Ethics Panel 1 (2024-21589-38791; 20-12-2024). It has been written in accordance with the SPIRIT statement [43] and has been prospectively registered with the ISRCTN Ref: ISRCTN: ISRCTN12744098. There is an independent Trial Steering Committee (TSC). The University of Manchester has in place research study insurance against liabilities for which it may be legally liable, and this cover includes any such liabilities arising out of this study.

### Trial Status

As of the time of this publication, the study is actively underway in accordance with protocol version 2.3, dated January 15, 2026 (see S2 Detailed Protocol in S2 File). Recruitment of participants started in June 2025 and is anticipated to be completed by March 31^st^, 2026. The intervention phase and the last follow-up assessments are expected to be concluded by February 2027. The study is progressing according to the planned timeline, and data collection and analysis will commence thereafter to generate meaningful results.

### Limitations

This study recognises several limitations. One such limitation is the use of self-reported outcomes rather than researcher-rated measures, which potentially leaves outcomes as vulnerable to response bias and inconsistencies in understanding. Despite this potential limitation, self-reported outcomes were chosen as a convenient and efficient way for participants to capture their unique, subjective experience of study participation.

A sample related limitation to consider is that participants are self-selecting medical students who respond to recruitment invitations, which may introduce selection bias if those motivated to participate differ systematically from the broader population of medical students. A further point of consideration is that self-selecting participants may not be part of the target population, this is an issue with remunerated participation whereby potentially fake participants sign up for the incentives associated with study participation. This study has attempted to reduce the impact of incentivisation by only remunerating for the follow up questionnaires (T2, T3 and T4) at a relatively small amount.

A comparator group limitation to consider is the control condition (services as usual) is not a standardised intervention, as mental health support varies between medical schools, making the comparison less precise.

## Discussion

Here we describe a Randomised Control Trial (RCT) evaluating a cognitive-behaviourally based supportive intervention tailored for medical students. This work builds on earlier pilot studies of Reboot, the intervention on whose principles Thumos is based, which found it was acceptable, feasible and potentially effective in a range of healthcare professional and student groups [9,22–26]. The present study includes important improvements to Reboot and will provide the first definitive evidence of effectiveness. As such, this study represents a step-change in evidence quality and a springboard for implementation.

Mental health distress is a significant problem in medical students [10–12], but existing interventions show limited effectiveness [19,21]. These interventions are also often time-consuming, involving several sessions spaced over weeks or months, which are not always feasible for medical students to attend. In contrast, Thumos comprises only 5 hours of contact, including two online workshops and one one-to-one phone call which can be flexibly scheduled according to students’ timetables. The present study will provide information to relevant bodies to consider whether they want to invest in Thumos provision. Provision could be made at the local (medical school) or national (e.g., Medical Schools Council; British Medical Association) level and delivery would be made possible via a train-the-trainer model, whereby existing psychological therapists could be trained in the intervention protocol via a workshop and observations.

The study will focus on the effectiveness of Thumos in medical students. However, if findings indicate it is effective, it could be adapted and tailored for any healthcare student or professional group internationally, including those working in the private sector and dental professionals (including dentists and dental nurses) [17–20].

## Conclusion

This RCT of Thumos represents a critical step forward in addressing the mental health crisis among medical students. By providing a tailored, prevention-focused intervention that combines group workshops with personalised support, Thumos directly targets the specific stressors medical students encounter during clinical placements. If successful, Thumos could be widely implemented, offering a pragmatic, evidence-based solution to a pervasive problem. The implications extend beyond medical education—by supporting the wellbeing of future healthcare professionals, we could help break the vicious cycle linking poor staff wellbeing with inadequate staffing and compromised patient care.

## Supporting information

S1 File CONSORT Checklist. (DOCX)

S2 File Detailed Protocol. (DOCX)

S3 File Appendix 1. (DOCX)

S4 File Appendix 2. (DOCX)

## References

[1] GBD 2019 Human Resources for Health Collaborators. Measuring the availability of human resources for health and its relationship to universal health coverage for 204 countries and territories from 1990 to 2019: a systematic analysis for the Global Burden of Disease Study 2019. Lancet. 2022;399(10341):2129–54. doi: 10.1016/S0140-6736(22)00532-3 35617980 PMC9168805

[2] The Health Foundation, The Kingsfund, Nuffield Trust. Closing the gap: Key areas for action on the health and care workforce [Internet]. 2019 [cited 2019 Sep 20]. Available from: https://www.kingsfund.org.uk/sites/default/files/2019-03/closing-the-gap-health-care-workforce-overview_0.pdf

[3] Dall’Ora C, Saville C, Rubbo B, Turner L, Jones J, Griffiths P. Nurse staffing levels and patient outcomes: A systematic review of longitudinal studies. Int J Nurs Stud. 2022;134:104311. doi: 10.1016/j.ijnurstu.2022.104311 35780608

[4] Demerouti E, Bakker AB. The Oldenburg Burnout Inventory: A good alternative to measure burnout and engagement. Handbook of stress and burnout in health care. 2008;65(7):1–25.

[5] Toppinen-Tanner S, Ojajärvi A, Väänänen A, Kalimo R, Jäppinen P. Burnout as a predictor of medically certified sick-leave absences and their diagnosed causes. Behav Med. 2005;31(1):18–27. doi: 10.3200/BMED.31.1.18-32 16078523

[6] Bae S-H, Cho M, Kim O, Pang Y, Cha C, Jung H, et al. Predictors of actual turnover among nurses working in Korean hospitals: A nationwide longitudinal survey study. J Nurs Manag. 2021;29(7):2102–14. doi: 10.1111/jonm.13347 33894028

[7] Hodkinson A, Zhou A, Johnson J, Geraghty K, Riley R, Zhou A, et al. Associations of physician burnout with career engagement and quality of patient care: systematic review and meta-analysis. BMJ. 2022;378:e070442. doi: 10.1136/bmj-2022-070442 36104064 PMC9472104

[8] Vogt KS, Simms-Ellis R, Grange A, Griffiths ME, Coleman R, Harrison R, et al. Critical care nursing workforce in crisis: A discussion paper examining contributing factors, the impact of the COVID-19 pandemic and potential solutions. J Clin Nurs. 2023;32(19–20):7125–34. doi: 10.1111/jocn.16642 36823696

[9] Johnson J, Pointon L, Talbot R, Coleman R, Budworth L, Simms-Ellis R, et al. Reboot coaching programme: a mixed-methods evaluation assessing resilience, confidence, burnout and depression in medical students. Scott Med J. 2024;69(1):10–7. doi: 10.1177/00369330231213981 38050379 PMC10986146

[10] Rotenstein LS, Ramos MA, Torre M, Segal JB, Peluso MJ, Guille C, et al. Prevalence of depression, depressive symptoms, and suicidal ideation among medical students: a systematic review and meta-analysis. JAMA. 2016;316(21):2214–36. doi: 10.1001/jama.2016.17324 27923088 PMC5613659

[11] Puthran R, Zhang MWB, Tam WW, Ho RC. Prevalence of depression amongst medical students: a meta-analysis. Med Educ. 2016;50(4):456–68. doi: 10.1111/medu.12962 26995484

[12] Dyrbye LN, Thomas MR, Massie FS, Power DV, Eacker A, Harper W, et al. Burnout and suicidal ideation among U.S. medical students. Ann Intern Med. 2008;149(5):334–41. doi: 10.7326/0003-4819-149-5-200809020-00008 18765703

[13] British Medical Association. Four in ten junior doctors plan to leave the NHS as soon as they can find another job, BMA council chair reveals in New Year’s message [Internet]. 2022 [cited 2023 Jun 23]. Available from: https://www.bma.org.uk/bma-media-centre/four-in-ten-junior-doctors-plan-to-leave-the-nhs-as-soon-as-they-can-find-another-job-bma-council-chair-reveals-in-new-years-message-to-the-country

[14] Tolksdorf KH, Tischler U, Heinrichs K. Correlates of turnover intention among nursing staff in the COVID-19 pandemic: a systematic review. BMC Nurs. 2022;21(1):174. doi: 10.1186/s12912-022-00949-4 35787700 PMC9252069

[15] Hill MR, Goicochea S, Merlo LJ. In their own words: stressors facing medical students in the millennial generation. Med Educ Online. 2018;23(1):1530558. doi: 10.1080/10872981.2018.1530558 30286698 PMC6179084

[16] Hu KS, Chibnall JT, Slavin SJ. Maladaptive perfectionism, impostorism, and cognitive distortions: threats to the mental health of pre-clinical medical students. Acad Psychiatry. 2019;43(4):381–5. doi: 10.1007/s40596-019-01031-z 30725427

[17] Weurlander M, Lönn A, Seeberger A, Broberger E, Hult H, Wernerson A. How do medical and nursing students experience emotional challenges during clinical placements?. Int J Med Educ. 2018;9:74–82. doi: 10.5116/ijme.5a88.1f80 29587248 PMC5952306

[18] Nteveros A, Kyprianou M, Artemiadis A, Charalampous A, Christoforaki K, Cheilidis S, et al. Burnout among medical students in Cyprus: A cross-sectional study. PLoS One. 2020;15(11):e0241335. doi: 10.1371/journal.pone.0241335 33206654 PMC7673498

[19] Witt K, Boland A, Lamblin M, McGorry PD, Veness B, Cipriani A, et al. Effectiveness of universal programmes for the prevention of suicidal ideation, behaviour and mental ill health in medical students: a systematic review and meta-analysis. Evid Based Ment Health. 2019;22(2):84–90. doi: 10.1136/ebmental-2019-300082 30918000 PMC10270399

[20] Chew-Graham CA, Rogers A, Yassin N. “I wouldn’t want it on my CV or their records”: medical students’ experiences of help-seeking for mental health problems. Med Educ. 2003;37(10):873–80. doi: 10.1046/j.1365-2923.2003.01627.x 12974841

[21] Sperling EL, Manion B, Gresner E, LaBarre A, Krych P, Mallender ZC. The effect of resilience training on resilience and stress in medical students: a systematic review and meta-analysis. BMC Med Educ. 2025;26(1):7. doi: 10.1186/s12909-025-08171-x 41316127 PMC12771728

[22] Johnson J, Simms-Ellis R, Janes G, Mills T, Budworth L, Atkinson L, et al. Can we prepare healthcare professionals and students for involvement in stressful healthcare events? A mixed-methods evaluation of a resilience training intervention. BMC Health Serv Res. 2020;20(1):1094. doi: 10.1186/s12913-020-05948-2 33246457 PMC7691965

[23] Janes G, Harrison R, Johnson J, Simms-Ellis R, Mills T, Lawton R. Multiple meanings of resilience: Health professionals’ experiences of a dual element training intervention designed to help them prepare for coping with error. J Eval Clin Pract. 2022;28(2):315–23. doi: 10.1111/jep.13555 33665919

[24] Vogt KS, Grange A, Johnson J, Marran J, Budworth L, Coleman R, et al. Study protocol for the online adaptation and evaluation of the “Reboot” (Recovery-boosting) coaching programme, to prepare critical care nurses for, and aid recovery after, stressful clinical events. Pilot Feasibility Stud. 2022;8(1):63. doi: 10.1186/s40814-022-01014-2 35300720 PMC8927745

[25] Vogt KS, Johnson J, Coleman R, Simms-Ellis R, Harrison R, Shearman N, et al. Can the Reboot coaching programme support critical care nurses in coping with stressful clinical events? A mixed-methods evaluation assessing resilience, burnout, depression and turnover intentions. BMC Health Serv Res. 2024;24(1):343. doi: 10.1186/s12913-023-10468-w 38491374 PMC10941361

[26] Al-Ghunaim T, Johnson J, Biyani CS, Coleman R, Simms-Ellis R, O’Connor DB. Evaluation of the reboot coaching workshops among urology trainees: A mixed method approach. BJUI Compass. 2023;4(5):533–42. doi: 10.1002/bco2.249 37636204 PMC10447217

[27] Dyrbye LN, Eacker A, Durning SJ, Brazeau C, Moutier C, Massie FS, et al. The Impact of Stigma and Personal Experiences on the Help-Seeking Behaviors of Medical Students With Burnout. Acad Med. 2015;90(7):961–9. doi: 10.1097/ACM.0000000000000655 25650824

[28] Beck AT, Rush AJ, Shaw BF, Emery G. Cognitive Therapy of Depression. New York: Guilford Press; 1979.

[29] Hofmann SG, Asnaani A, Vonk IJJ, Sawyer AT, Fang A. The Efficacy of Cognitive Behavioral Therapy: A Review of Meta-analyses. Cognit Ther Res. 2012;36(5):427–40. doi: 10.1007/s10608-012-9476-1 23459093 PMC3584580

[30] Johnson J, Panagioti M, Bass J, Ramsey L, Harrison R. Resilience to emotional distress in response to failure, error or mistakes: A systematic review. Clin Psychol Rev. 2017;52:19–42. doi: 10.1016/j.cpr.2016.11.007 27918887

[31] Smith BW, Dalen J, Wiggins K, Tooley E, Christopher P, Bernard J. The brief resilience scale: assessing the ability to bounce back. Int J Behav Med. 2008;15(3):194–200. doi: 10.1080/10705500802222972 18696313

[32] Löwe B, Unützer J, Callahan CM, Perkins AJ, Kroenke K. Monitoring depression treatment outcomes with the patient health questionnaire-9. Med Care. 2004;42(12):1194–201. doi: 10.1097/00005650-200412000-00006 15550799

[33] Keetharuth AD, Rowen D, Bjorner JB, Brazier J. Estimating a Preference-Based Index for Mental Health From the Recovering Quality of Life Measure: Valuation of Recovering Quality of Life Utility Index. Value Health. 2021;24(2):281–90. doi: 10.1016/j.jval.2020.10.012 33518035 PMC7871010

[34] Yoon S, Lee Y, Han C, Pae C-U, Yoon H-K, Patkar AA, et al. Usefulness of the Patient Health Questionnaire-9 for Korean medical students. Acad Psychiatry. 2014;38(6):661–7. doi: 10.1007/s40596-014-0140-9 24804631

[35] Cameron IM, Crawford JR, Lawton K, Reid IC. Psychometric comparison of PHQ-9 and HADS for measuring depression severity in primary care. Br J Gen Pract. 2008;58(546):32–6. doi: 10.3399/bjgp08X263794 18186994 PMC2148236

[36] Skivington K, Matthews L, Simpson SA, Craig P, Baird J, Blazeby JM, et al. A new framework for developing and evaluating complex interventions: update of Medical Research Council guidance. BMJ. 2021;374:n2061. doi: 10.1136/bmj.n2061 34593508 PMC8482308

[37] Sekhon M, Cartwright M, Francis JJ. Development of a theory-informed questionnaire to assess the acceptability of healthcare interventions. BMC Health Serv Res. 2022;22(1):279. doi: 10.1186/s12913-022-07577-3 35232455 PMC8887649

[38] Benjamini Y, Hochberg Y. Controlling the false discovery rate: a practical and powerful approach to multiple testing. J R Stat Soc Series B Methodol. 1995;57(1):289–300.doi: 10.1111/j.2517-6161.1995.tb02031.x

[39] Braun V, Clarke V. Reflecting on reflexive thematic analysis. Qualitative Research in Sport, Exercise and Health. 2019;11(4):589–97. doi: 10.1080/2159676x.2019.1628806

[40] Kendal S, Louch G, Walker L, Shafiq S, Halligan D, Brierley-Jones L, et al. Implementing and evaluating patient-focused safety technology on adult acute mental health wards. J Psychiatr Ment Health Nurs. 2024;31(5):742–54. doi: 10.1111/jpm.13028 38279658

[41] Guetterman TC, Mitchell N. The role of leadership and culture in creating meaningful assessment: A mixed methods case study. Innov High Educ. 2016;41(1):43–57.

[42] Walton JB, Plano Clark VL, Foote LA, Johnson CC. Navigating Intersecting Roads in a Mixed Methods Case Study: A Dissertation Journey. Journal of Mixed Methods Research. 2019;14(4):436–55. doi: 10.1177/1558689819872422

[43] Chan A-W, Tetzlaff JM, Altman DG, Laupacis A, Gøtzsche PC, Krleža-Jerić K, et al. SPIRIT 2013 statement: defining standard protocol items for clinical trials. Ann Intern Med. 2013;158(3):200–7. doi: 10.7326/0003-4819-158-3-201302050-00583 23295957 PMC5114123

