# Supplementary material for: ACTIVATE: A protocol for a randomised controlled evaluation of Thumos: A cognitive-behavioural intervention for medical students
Source: PLoS One. 2026 Sep 25;21(9):e0355371. doi: 10.1371/journal.pone.0355371 (PMC13614533; doi:10.1371/journal.pone.0355371)
Supplement: Supplementary file 2 [file pone.0355371.s002.docx]

|  | ***ACTIVATE*** |  |
| --- | --- | --- |
|  | **Full Title**  **ACTIVATE:** **A** protocol for a randomised **C**ontrolled evalua**t**ion of **T**humos: a cogn**i**tive-beha**v**ioural **I**nter**Ve**ntion for medic**A**l s**T**ud**E**nts |  |
|  | **Short title**  ACTIVATE: A protocol for a randomised controlled trial of a cognitive behavioural intervention (THUMOS) |  |
|  | **RESEARCH PROTOCOL**  **(Version 2.3) 15^th^ January 2026** |  |
|  | **REC Reference: 2024-21589-38791**  **Sponsor Reference: 2024-21589-38791**  **Funding Reference: TMPSF2024001** **ISRCTN: ISRCTN12744098**  **Authorised by: Dr Judith Johnson** | |

**The University of Manchester: Faculty of Biology, Medicine and Health**

## Protocol amendments

| **Document ID** | **Description of changes from previous version** | **Effective**  **Date** |
| --- | --- | --- |
| Protocol V 1.0 | First Protocol submitted for review by university UREC committee | 20.12.2024  UREC favourable ethical opinion received.  UREC Ref:  2024-21589-38791 |
| Protocol V 2.0 | 1) Update the flow chart to include ReQoL and sickness absence  2) Add in these two measures as measures for T1/2/3/4 questionnaires  3) Inclusion of new edited process evaluation questionnaires  4) Question about service use is added.  5) Added detail about following people up for the adverse events item in T2. | 20.03.2025  Amendment approved by university ethics committee  UREC Amendment Ref: 2025-21589-40219 |
| Protocol V 2.1 | Minor amendment submitted:  1) Updated AE questionnaire Item to include more detail about potential examples of adverse events | 13.05.2025  Amendment approved by university ethics committee  UREC Amendment Ref: 2025-21589-41297 |
| Protocol V 2.2 | Minor amendment submitted:  1) Updated recruitment text and materials to include more details about exclusion/inclusion criteria (e.g. example year of study) and additional details about the intervention arm of the study (e.g. number of workshops). | 21.10.2025  Amendment approved by university ethics committee  UREC Amendment Ref: 2025-21589-44079 |
| Protocol V 2.3 | Minor amendments submitted:  1) Increase recruitment by the number of participants who withdraw following randomisation but by no more than 10% overall.  2) A tightening of the screening process through contacting participants who have not provided a university email address during completion of consent and baseline measures, by contacting them and requesting a university email address at T2 and T3. The addition of a screening question as part of the baseline measure to collect university emails for newly consenting participants in T1. | 11.12.2025  Amendment approved by university ethics committee  UREC Amendment Ref: 2025-21589-44883 |

**ACTIVATE:** **A** protocol for a randomised **C**ontrolled evalua**t**ion of **T**humos: a cogn**i**tive-beha**v**ioural **I**nter**Ve**ntion for medic**A**l s**T**ud**E**nts

Acronym: **ACTIVATE**

This document describes a randomised controlled trial,and provides information about procedures for entering participants. Amendments may be necessary; these will be circulated to all interested parties.

## Abstract

**Background**

Medical students experience high rates of burnout and mental health distress, with approximately half reporting burnout, one-third experiencing depression, and one-tenth having suicidal thoughts. These issues are exacerbated during clinical placements and contribute to workforce attrition after qualification. Existing interventions are largely generic and show limited effectiveness. Thumos, a cognitive-behavioural therapy-based intervention specifically tailored for healthcare professionals and medical students, addresses the stressful situations intrinsic to medical training and practice. This randomised controlled trial will evaluate whether Thumos improves psychological resilience, confidence in dealing with stressful situations, burnout, and depression in medical students compared with services as usual (SAU).

**Methods/design:**
Two hundred and twenty UK medical students in a year that contains a clinical placement of a week or longer will be recruited nationally from UK medical schools. Participants will be randomised 1:1 to receive either Thumos (two online group workshops and one individual video/phone call) or SAU. Assessments will occur at baseline, post-intervention, 4-month, and 9-month follow-up. The primary outcome is psychological resilience measured by the Brief Resilience Scale at 4-month follow-up. A mixed-methods process evaluation will assess acceptability and feasibility.

**Discussion:**
To our knowledge, this is the first randomised controlled trial of an intervention specifically designed to prepare medical students for stressful clinical events. If effective, Thumos could potentially be adapted for medical students in other countries.

**Contents**

[Protocol amendments	2](#_Toc219465157)

[Abstract	4](#_Toc219465158)

[Abbreviations	7](#_Toc219465159)

[Definition of terms	7](#_Toc219465160)

[1. General information	8](#_Toc219465161)

[1.1 Investigator details	8](#_Toc219465162)

[1.2 Sponsor Details	8](#_Toc219465163)

[1.3 Role of the Funder	8](#_Toc219465164)

[1.4 Protocol amendments	8](#_Toc219465165)

[Trial Summary	9](#_Toc219465166)

[2. Introduction	11](#_Toc219465167)

[2.1 Background	11](#_Toc219465168)

[2.2 Rationale for current study	13](#_Toc219465169)

[3. Aims and objectives	13](#_Toc219465170)

[4. Trial Design	14](#_Toc219465171)

[4.1 Blinding	14](#_Toc219465172)

[4.2 Unblinding	15](#_Toc219465173)

[5. Selection of participants	15](#_Toc219465174)

[Study population for randomised controlled trial	17](#_Toc219465175)

[5.1 Inclusion criteria	17](#_Toc219465176)

[5.2 Exclusion criteria	17](#_Toc219465177)

[Study population for process evaluation	17](#_Toc219465178)

[5.3 Inclusion criteria	17](#_Toc219465179)

[5.4 Exclusion criteria	17](#_Toc219465180)

[5.5 Participant identification	17](#_Toc219465181)

[5.6 Informed consent process	18](#_Toc219465182)

[6. Trial treatment	18](#_Toc219465183)

[6.1 Participants randomised to THUMOS	18](#_Toc219465184)

[6.2 Participants randomised to Control Group	18](#_Toc219465185)

[7. Randomisation and enrollment	19](#_Toc219465186)

[8. Outcomes	19](#_Toc219465187)

[8.1 Primary outcome	20](#_Toc219465188)

[8.2 Secondary outcomes	20](#_Toc219465189)

[8.3 Process evaluation outcomes	22](#_Toc219465190)

[9. Assessments and procedures	23](#_Toc219465191)

[9.1 Study assessments schedule	23](#_Toc219465192)

[9.2 Procedures for treatment fidelity	24](#_Toc219465193)

[9.3 Procedure for assessing safety	24](#_Toc219465194)

[9.4 Participant withdrawals	24](#_Toc219465195)

[9.5 Lost to contact	25](#_Toc219465196)

[10. Safety Reporting	25](#_Toc219465197)

[10.1 Definitions	25](#_Toc219465198)

[11. Statistics	28](#_Toc219465199)

[11.1 Sample size	28](#_Toc219465200)

[11.2 Statistical Analysis	29](#_Toc219465201)

[12. Trial supervision	31](#_Toc219465202)

[13.1 Trial Steering Committee	31](#_Toc219465203)

[13.2 Data Monitoring and Ethics Committee	32](#_Toc219465204)

[13.3 Stopping Guidelines	32](#_Toc219465205)

[13.4 Trial Management Group	33](#_Toc219465206)

[14. Data handling and record keeping	33](#_Toc219465207)

[15. Data access and quality assurance	34](#_Toc219465208)

[16. Publication	34](#_Toc219465209)

[17. Finance	35](#_Toc219465210)

[18. Ethics approval & regulatory compliance	35](#_Toc219465211)

[19. Medical Student Involvement	35](#_Toc219465212)

[22. Indemnity / Compensation / Insurance	35](#_Toc219465213)

[23. References	35](#_Toc219465214)

[Appendices	38](#_Toc219465215)

[Appendix 1.0	38](#_Toc219465216)

[1.0. List of eligible UK medical schools	38](#_Toc219465217)

[Appendix 2.0	40](#_Toc219465218)

[2.0 Participant Information Sheet and Consent Form	40](#_Toc219465219)

[Appendix 3.0	49](#_Toc219465220)

[ACTIVATE Statistical Analysis Plan (SAP)	49](#_Toc219465221)

# Abbreviations

## Definition of terms

AE	Adverse Event

CBT	Cognitive-Behavioural Therapy

CI	Chief Investigator

CRF	Case Report Form

DMEC	Data Monitoring and Ethics Committee

GCP	Good Clinical Practice

ISRCTN	International Standard Randomised Controlled Trials	Number

MPS	Medical Protection Society

MRC	Medical Research Council

PCF	Participant Consent Form

PIS	Participant Information Sheet

RCT	Randomised Controlled Trial

REC	Research Ethics Committee

SAE	Serious Adverse Event

SAP	Statistical Analysis Plan

SAU	Services As Usual

SOP	Standard Operating Procedure

TMG	Trial Management Group

TSC	Trial Steering Committee

# 1. General information

## 1.1 Investigator details

**Chief Investigator:**

Dr Judith Johnson

*Reader, School of Health Sciences*

*Director, Manchester Catalys*

*University of Manchester*

*Jean McFarlane Building*

*176 Oxford Rd*

*Manchester*

*M13 9PY*

## 1.2 Sponsor Details

University of Manchester

Oxford Rd,

Manchester

M13 9PL

## 1.3 Role of the Funder

The Medical Protection Society (MPS) Foundation

The funder has reviewed the research protocol but will have no role in data collection, analysis, data interpretation, report writing or in the decision to submit the report for publication.

## 1.4 Protocol amendments

NONE

Trial Summary

| Study title | ACTIVATE: A randomised Controlled evaluation of Thumos: a cognitive-behavioural InterVention for medicAl sTudEnts |
| --- | --- |
| Sponsor | The University of Manchester |
| Funder | The MPS Foundation |
| ISRCTN | ISRCTN12744098 <https://doi.org/10.1186/ISRCTN12744098> |
| Project start date | 01/12/2024 |
| Project end date | 31/03/2027 |
| Aims and objectives | To establish whether medical students receiving Thumos, a cognitive behavioural intervention, will have improved psychological resilience at 4 month follow up in comparison to medical students receiving services as usual (SAU).  Secondary objectives are:   1. To determine whether Thumos is superior to SAU at 9-month follow-up in increasing psychological resilience. 2. To determine whether Thumos is superior to SAU at 4-month and 9-month follow-ups in the endpoints of confidence, burnout, depression, mental health and self-reported sickness absence. |
| Trial design | Randomised controlled parallel groups intervention trial |
| Setting | Online |
| Participants | UK medical students in years involving clinical placements of a week or longer nationally. We will recruit from these groups for the main trial until the recruitment target is reached. |
| Intervention & control groups | UK medical students in clinical placement years will be randomised 1:1 to receive either (Intervention group) Thumos (a CBT intervention of two online group workshops and one individual video/phone call) or (Control group) Services As Usual (SAU). |
| Primary outcome(s) | To determine whether Thumos is superior to SAU at a 4-month follow-up in increasing psychological resilience. |
| Secondary outcome(s) | To determine whether Thumos is superior to SAU at a 9-month follow-up in increasing psychological resilience. To determine whether Thumos is superior to SAU at 4-month and 9-month follow-ups in the endpoints of confidence, burnout, depression, mental health and self-reported sickness absence. |
| Duration of recruitment period and first enrolment date | Recruitment period to run from 1/06/2025 to 31/03/2026. Date of first enrollment is 16/06/2025. |
| Duration of follow-up | Nine months from enrollment. |
| Target sample size | 220 |
| Definition of end of trial | The end of trial is defined as the collection of data from the last recruited participants’ follow-up questionnaire (9-month follow up). |

# 2. Introduction

## 2.1 Background

Healthcare systems internationally are experiencing a workforce crisis, with a current global shortage of six million physicians [1]. While poor workforce planning has contributed to this problem, high turnover has been identified as the main contributing factor [2]. Inadequate staffing is linked with negative patient outcomes including extended hospital stays and mortality [3]. It also creates a vicious cycle, whereby existing staff become overworked and burnt-out, a syndrome characterised by exhaustion and disengagement [4]. Higher burnout then increases the risk of sickness absence and turnover, further exacerbating staffing shortages [5, 6] and directly leading to the delivery of poorer patient care [7].

In a review of 170 studies in doctors, higher burnout was associated with twice the risk of patient safety incidents and a two-fold reduction in patient satisfaction [7]. Within this vicious cycle, provider burnout and poor wellbeing must be recognised as an active component, serving to drive the workforce crisis. Equally, addressing this component by better supporting healthcare professionals could help to break this cycle, leading to reduced sickness absence, better retention and better patient care [8].

Most burnout research has focused on qualified professional groups, but there is a growing recognition that it could be beneficial to intervene during training, prior to professional registration, due to high rates of mental health distress in these cohorts [9]. Studies in medical students indicate half report burnout, one in three report depression and one in ten experience suicidal thoughts [10-12]. Furthermore, the immediate post-qualification years have been recognised as a high-risk time for physicians leaving the profession [13]. Poor mental health and burnout are linked with higher attrition [14], so improving their mental wellbeing could be one way to enhance retention and reduce physician shortages.

Multiple risk factors unique to medical training have been implicated in these mental health difficulties, notably demanding academic requirements and perfectionism-related cognitive patterns [15, 16] and exposure to stressful clinical events on placement [17]. Indeed, rates of burnout in medical students increase significantly during the years involving clinical placements [18]. However, there has been a lack of interventions tailored for medical students and none which prepare medical students for stressful workplace events [19]. Previous evaluations of mental health support for medical students have predominantly examined non-tailored approaches such as mindfulness-based programmes, relaxation techniques, and yoga. [19]. Evidence for these interventions is weak and mixed, and they have been criticised for lacking relevance [20]. A recent meta analysis of resilience interventions for medical students indicated that these interventions are moderately effective at reducing stress and mildly affective at increasing resilience within medical students [21]. The implication that these interventions may have more favourable impacts on stress suggests that further research is required to examine the specific factors that influence resilience. There is an urgent need for effective, preventative interventions which help students cope with the specific occupational stressors they will encounter during their placements, training, and beyond.

The current trial will evaluate Thumos, an intervention that employs cognitive-behavioural therapy (CBT) techniques to prepare healthcare professionals and students to manage stressful work or training events. This approach has been investigated, tested and developed in several studies previously [9, 22-24]. The approach used in Thumos differs from many previously evaluated psychological interventions for medical students in three ways. Firstly, it has been designed with healthcare professionals in mind, based upon a combination of 1) research into the effects of patient safety incidents and other work stressors, 2) resilience theory, and 3) CBT techniques. Secondly, Thumos integrates complementary therapeutic formats: group sessions that facilitate peer connection, alongside an individual consultation that provides personalised support. Thirdly, Thumos addresses the stressful situations that are intrinsic to healthcare work, providing support for those unavoidable events that cannot be improved through organisational interventions.

Characteristics of Thumos may make it particularly well suited for medical students. For example, the preventive framing of the intervention may help address barriers related to help-seeking stigma, which research has identified as a significant obstacle to engagement with mental health support among medical students [11, 25, 26]. It also uses case studies and materials which are tailored towards the stressful experiences medical students may face on placements. This approach increases the sense of relevance of the intervention for participants which may increase engagement [9]. Furthermore, it directly addresses unhelpful perfectionist thinking styles that have been associated with psychological distress in this population [16].

There have been three uncontrolled evaluations of of versions of Reboot, a CBT-based intervention for medical professionals which indicate positive findings. The first was in a group of 66 multi-disciplinary healthcare professionals and students; results indicated it was associated with improvements in participants’ confidence, coping knowledge and psychological resilience [22, 27]. The second was in 80 Critical Care Nurses (CCNs) [23, 28]. Participation was associated with increased confidence and resilience and reduced depression and burnout. Nurses also reported a lower intention to leave clinical practice following the intervention [23]. The third evaluation was in 115 medical students [9]. Findings indicated the intervention was feasible and potentially effective in this group, with participants reporting significant increases in resilience and confidence in coping with adverse events, and significant decreases in burnout and depression [9]. Qualitative findings from these evaluations indicate that participants appreciate the intervention’s tailored format and opportunity for peer support alongside professional input. They describe increased self-awareness and reported the acquisition of psychological skills which they could apply to cope with work stressors [22, 23, 27].

## 2.2 Rationale for current study

Feedback from these evaluations was used to make improvements to, and to further tailor, the intervention towards medical students, including new exercises, reduction of didactic information and changing the phone call discussion. There is now a need to test the intervention, Thumos, and to ascertain whether observed improvements in outcome indicators can be attributed to the intervention. This study addresses this gap, through a definitive randomised controlled trial (RCT). The comparator intervention is Services as Usual (SAU); this comparison will establish whether and what benefits Thumos may offer above what is currently available. The final follow-up time point is 9 months and create the possibility of conducting a subsequent 5-year follow-up study, should initial results be positive.

# 3. Aims and objectives

The main purpose of the trial is to establish whether Thumos increases resilience and improves mental health in medical students. The primary objective is to determine whether Thumos is superior to the control condition (services as usual; SAU) at 4 month follow-up in increasing psychological resilience in medical students. Secondary objectives are:

1. To determine whether Thumos is superior to SAU at 9-month follow-up in increasing psychological resilience.
2. To determine whether Thumos is superior to SAU at 4-month and 9-month follow-ups in the endpoints of confidence, burnout, depression, mental health and self-reported sickness absence.
3. To assess acceptability and how Thumos is delivered in practice through a process evaluation with users, medical educators and providers

# 4. Trial Design

This study is a randomised controlled parallel groups intervention trial, conducted across multiple medical schools in the United Kingdom (UK). All UK medical schools will be invited to participate (see Appendix 1.0). Participants will be self-selecting and screened for eligibility based on the criteria that they are a) UK medical student and b) in a year of study that contains a placement of one week or longer. The trial will compare Thumos to a control condition of services as usual (SAU), where medical students in either condition can continue to access available mental health support as they usually would. Data collection will occur at four timepoints, Time 1 (T1), pre-intervention (baseline); Time 2 (T2), post-intervention (following completion of Thumos/6 weeks after baseline for control group); Time 3 (T3), 4 months following baseline and Time 4 (T4), 9 month following baseline. All elements will be conducted online, with questionnaires administered via Qualtrics and interviews/intervention sessions via online video platform. Informed consent will also be taken online, with participants asked to read the information sheet and provide informed consent prior to responding to any data collection items (see Appendix 2.0). A process evaluation will be conducted which will include medical student participants, medical educators from medical schools of participating students and the Thumos therapists.

We will request permission to contact participants for further follow-up data at 2 and 5-years. If T4 indicates Thumos is effective, we can seek funding and a new ethics approval to conduct a subsequent study of long-term outcomes.

## 4.1 Blinding

Randomisation to the two groups will be undertaken following completion of baseline assessments using the web-based Sealed Envelope software. Randomisation will be in the ratio 1:1 to the two groups. The randomisation system will ensure blinding of the statistician. The CBT therapists, researchers and participants will be unblind.

## 4.2 Unblinding

There is no procedure for unblinding participants and CBT therapists as they are already unblinded. However, in the event that a Data Monitoring and Ethics committee (DMEC) is required for the study, the DMEC will be established and will be able to request unblinded data and recommend study termination to the Trial Steering Committee (TSC) on the grounds of safety or futility should any SAEs occur that are deemed related to the trial.

# 5. Selection of participants

The selection of participants for the randomised controlled trial and the process evaluation is described below in Figure 1.

Fig 1: Flow diagram of participants through the trial

## Study population for randomised controlled trial

# 5.1 Inclusion criteria

UK medical students in years involving clinical placements of a week or longer nationally. We will recruit from these groups to the main trial until recruitment numbers are saturated.

## 5.2 Exclusion criteria

We will not exclude anyone who meets the inclusion criteria. However, we will stop recruiting once we have reached our maximum number of participants to recruit (n=220) and exclude all prospective participants at this time.

## Study population for process evaluation

## 5.3 Inclusion criteria

Participating medical students: We will recruit medical students in the main trial to also take part in the process evaluation. Both control and intervention arm students will be invited to complete process questionnaires; only intervention arm medical students will be invited to take part in qualitative interviews.

UK medical educators: We will recruit medical educators in schools with participating students within the intervention arm to qualitative interviews as part of the process evaluation.

The cognitive behavioural therapists/clinical psychologists delivering the intervention will be invited to qualitative interviews as part of the process evaluation.

## 5.4 Exclusion criteria

We will not exclude anyone who meets the inclusion criteria. However, for qualitative interviews, we will stop recruiting once we have reached data saturation, defined as ‘information redundancy’ where no new information or value is generated from the data.

## 5.5 Participant identification

Medical student participants will be recruited via the Medical Protection Society (emails to medical student members), the Medical Schools Council (email cascade to UK medical schools), direct email communications to heads of UK medical school and via posts on social media (LinkedIn, BlueSky, Twitter, Instagram).

For the process evaluation, medical educators in schools with participating students who were allocated to the intervention arm will be contacted via emails sent to heads of medical schools with participating students. Trial therapists will be contacted directly via email.

## 5.6 Informed consent process

Informed consent will be taken online via Qualtrics, with participants asked to read the information sheet and provide informed consent prior to responding to any data collection items.

# 6. Trial treatment

## 6.1 Participants randomised to THUMOS

Thumos is delivered over a 4-week period and consists of three core components: two online group workshops and one individual coaching call. The group workshops are conducted with around 6-8 participants and last two hours each. During these workshops, participants engage in both individual exercises and group-based activities. These are followed by a one-hour individual phone call, which allows for personalisation of the workshop content to each participant’s specific needs. All sessions are delivered remotely via video platform or phone. The intervention is delivered by qualified Clinical Psychologists or Cognitive Behavioural Therapists who have received specific training in the Thumos intervention protocol manual.

Intervention condition participants can continue to access mental health services that are usually available via medical schools, universities or to the public throughout the period that they are participating in the study.

## 6.2 Participants randomised to Control Group

Control condition participants will not receive an alternative intervention. However, they can continue to access mental health services that are usually available via medical schools, universities or to the public throughout the period that they are participating. Services available to medical students will vary depending upon what their university and medical school provides as standard, but all will be able to access NHS primary care services.

# 7. Randomisation and enrollment

Participants will be randomised using simple 1:1 allocation via the Sealed Envelope platform, which generates the allocation sequence and maintains allocation concealment to ensure investigators remain blinded to treatment assignment. With 220 participants undergoing individual-level randomisation, simple randomisation is expected to achieve adequate balance between treatment arms without the need for blocking or stratification. Any post-randomisation covariate imbalances will be addressed through standard statistical adjustment methods during analysis. As all quantitative outcomes are self-reported rather than researcher-rated, researcher blinding is not a relevant consideration.

# 8. Outcomes

**Outcome Measures**

A core online questionnaire set including measures of resilience (Brief Resilience Scale [29]), burnout (Oldenburg Burnout Inventory; OLBI [4]) confidence in coping with adverse events (CCAE [9]) depression (Patient Health Questionnaire, PHQ-9 [30]), mental health (Recovery of Quality of Life-Utility Index, ReQoL-UI)[31]) sickness absence and support access will be completed at T1, T2, T3 and T4. In addition, at T2 a process questionnaire will be added to this dataset.

Table 1:

| Table 1. Outcome measures | | | |
| --- | --- | --- | --- |
| Outcome | **Time** | **Variable definition** | **Measure** |
| BRS (primary) | T3/T4 | BRS mean score | Adjusted mean difference/Cohen’s *d* |
| CCAE | T3/T4 | CCAE mean score | As above |
| OLBI | T3/T4 | OLBI total and subscales | As above |
| PHQ-9 | T3/T4 | PHQ-9 total score | As above |
| ReQoL-UI | T3/T4 | Utility score | As above |
| ReQoL-UI QALYs | BL-T4 | QALYs area under the curve | Adjusted mean difference |
| Sickness absence | T3/T4 | Days lost in past 4 weeks | Adjusted rate ratios with 95% CI |
| BRS: Brief Resilience Scale; CCAE: Confidence in Coping with Adverse Events; OLBI: Oldenburg Burnout Inventory; PHQ-9: Patient Health Questionnaire-9 item depression scale; ReQoL-UI: Recovering Quality of Life – Utility Index; QALYs: Quality-adjusted life years; BL: Baseline; T1, T2, T3, T4: Time points 1, 2, 3, and 4 (T = time) | | | |

Table 2:

| Table 2. Outcome scoring | | | | | |
| --- | --- | --- | --- | --- | --- |
| Outcome | **Items** | **Measure** | **Construct(s) & direction** | **Time points** | **Derived variable/scoring** |
| BRS (Primary) | 6 | Brief Resilience Scale | Resilience; higher = better | T3 [12–24 wks] | Mean of 6 items (with required reverse scoring) |
| BRS | 6 | Brief Resilience Scale | Resilience; higher = better | T4 [30–44 wks] | Mean score |
| CCAE | 3 | Confidence in Coping with Adverse Events questionnaire | Confidence coping with adverse events; higher = better | T3, T4 [12–24, 30–44] | Mean of 3 items |
| OLBI (total & subscales) | 16 | Oldenburg Burnout Inventory | Burnout (exhaustion, disengagement); higher = worse | T3, T4 [12–24, 30–44] | Subscale means and total mean (with required reverse scoring) |
| PHQ-9 | 9 | Patient Health Questionnaire-9 | Depressive symptoms; higher = worse | T3, T4 [12–24, 30–44] | Sum of 9 items (0–27) |
| ReQoL-UI (utility) | 10 | Recovery of Quality of Life – Utility Index (ReQoL-UI) | Quality of life utility; higher = better | T3, T4 [12–24, 30–44] | Utility index via ReQoL-UI algorithm |
| ReQoL-UI QALYs | 10 | Recovery of Quality of Life – Utility Index (ReQoL-UI) | Quality adjusted life years; higher = better | Baseline–T4 | Area under utility time curve |
| Sickness absence | 2 | Sickness absence items | Days lost last 4 weeks; higher = worse | T3, T4 [12–24, 30–44] | Count of days lost in past 4 weeks |

## 8.1 Primary outcome

**To determine whether Thumos is superior to SAU at a 4-month follow-up in increasing psychological resilience.**

Brief Resilience Scale (BRS): The 6-item BRS measures perceptions of personal resilience, including ‘I tend to bounce back quickly after hard times’. This questionnaire has good internal reliability (α=.80-.91), concurrent validity with other resilience questionnaires [28], and detects learning changes [9].

## 8.2 Secondary outcomes

**To determine whether Thumos is superior to SAU at 4-month and 9-month follow-ups in the endpoints of confidence, burnout, depression, mental health and self-reported sickness absence.**

Oldenburg Burnout Inventory (OLBI). The scale comprises 16 items and two subscales (exhaustion and disengagement). It has been used widely and found to have good internal reliability (α=.88) [4].

Confidence in coping with Adverse Events (CCAE) questionnaire: This 3-item questionnaire assesses how confident participants are in coping with adverse events, e.g., ‘If I was involved in an adverse event for which I thought I held some responsibility I know the things I would do to help manage my stress levels’. Previous studies suggest this scale has good internal reliability (α=0.85) [24] and detects learning changes [9].

Patient-Health Questionnaire-9 (PHQ-9). This 9-item questionnaire is widely used to measure depression and has been validated in various groups, including medical students [32]. It has good internal consistency (α=.83) and captures fluctuations [33].

Recovery of Quality of Life-Utility Index (ReQoL-UI) [31]. Mental health will be measured with the 10-item ReQoL-UI. This validated, reliable questionnaire measures mental health across areas of quality of life. It applies to the full spectrum of mental health conditions and provides Quality of Life Adjusted Years (QALY) estimates.

Self-reported sickness absence. Two items will be used to measure sickness absence, the first which asks about total days lost within the past 4 weeks; the second of which asks how many working or studying days specifically were lost within this timeframe.

Support access. Four items will be used to assess whether participants are accessing any form of psychological wellbeing service, which type of support they are accessing, when they began accessing this, and when they anticipate, they will stop accessing this.

We are adjusting for age, medical school and gender.

## 8.3 Process evaluation outcomes

**To assess acceptability and how Thumos is delivered in practice through a process evaluation with users, medical educators and providers.**

We drew upon UK Medical Research Council (MRC) guidance for process evaluations of complex interventions [34] to design a mixed-methods approach to the process evaluation.

Psychological therapist logs: Weekly logs will assess therapists’ experiences of intervention delivery, including perceived benefits, mechanisms and challenges.

Process questionnaires: Process questionnaires at T2 (post-intervention) will explore participants’ reasons for participating, and their perceptions and experiences [35]. Participants in the intervention arm will be asked about their affective attitudes to the intervention, general acceptability, perceived effectiveness, ethicality, intervention coherence and mechanisms, self-efficacy, burdens, opportunity costs, and barriers and facilitators. Participants in the control arm will be asked about their reasons for participating, and their perceptions and experiences of the study.

Field notes: Researchers’ field notes will track any changes in the delivery of the intervention; enabling recording of protocol changes.

Participant interviews: At T3, a randomly selected subset of participants from the intervention arm will be invited to 30-minute semi-structured interviews (n=20) to generate information regarding intervention delivery, feasibility and acceptance, and barriers and facilitators to participation.

Medical educator interviews: Once all experimental participants have completed T2 data collection, medical educators (n=15) at schools where students have participated in the study will be interviewed to explore 1) their perceptions of the research process, and 2) perceived benefits or drawbacks of Thumos at an organisational level, 3) ways Thumos could be implemented at scale for medical students.

Therapist interviews: Once all experimental participants have completed T2 data collection, all trial therapists will be invited to 30-minute semi-structured interviews exploring their experiences of delivering the intervention.

# 9. Assessments and procedures

Primary and secondary outcomes will be collected via online questionnaires using Qualtrics software. Participants will be contacted at four timepoints (including baseline) via email with a link to complete the questionnaire.

## 9.1 Study assessments schedule

Table of measures used in questionnaire assessments.

Table 3:

|  | Baseline | T2  (post intervention or equivalent time) | T3  (4 month follow up) | T4  (9 month follow up) |
| --- | --- | --- | --- | --- |
| **Enrolment** |  |  |  |  |
| Informed consent form | X | - | - | - |
| **Primary outcome** |  |  |  |  |
| Brief Resilience Scale (BRS) | X | X | X | X |
| **Secondary outcomes** |  |  |  |  |
| Oldenburg Burnout Inventory (OLBI | X | X | X | X |
| Confidence in coping with Adverse Events (CCAE) questionnaire | X | X | X | X |
| Patient-Health Questionnaire-9 (PHQ-9) | X | X | X | X |
| Recovery of Quality of Life-Utility Index (ReQoL-UI) | X | X | X | X |
| Self-reported sickness absence | X | X | X | X |
| Support access | X | X | X | X |
| **Process evaluation** |  |  |  |  |
| Process questionnaires | - | X | - | - |

## 9.2 Procedures for treatment fidelity

Researchers will observe a minimum of 20% of workshop cycles and observe recordings of 10% of video/phone calls, using documented fidelity assessment criteria. These will consider how the intervention is conducted and assess fidelity. All medical student participants and Clinical Psychologists/CBT therapists delivering the intervention will be asked to provide their consent for this at baseline.

## 9.3 Procedure for assessing safety

The researchers will take all appropriate steps during the conduct of the trial for ensuring participant safety, in both arms of the trial. Concerns over safety of THUMOS or involvement in ACTIVATE identified through Adverse Events (AEs) and Serious Adverse Events (SAEs), as described in the follow-up questionnaires would, in the first instance, lead to protocol amendments, but could lead to study termination at any time, please refer to section 13.3 for our stopping guidelines. Our experience with this population and type of intervention suggests that THUMOS (intervention) proving unacceptable or too distressing to participants is a low risk. However, should this outcome occur, it would provide empirical evidence to inform future research.

## 9.4 Participant withdrawals

If a participant is found to be ineligible for the trial after consenting and providing baseline (T1) questionnaire responses, this will be communicated to them by email (including the reasons for their ineligibility) and the reason recorded.

Participants may withdraw their consent for the study at any time, without providing a reason for this. If a participant in either study arm tells us that they would like to withdraw following consent or randomisation, we will ask for their reasons why and record this. Although the participant is not required to give a reason for discontinuing their study participation, a reasonable effort will be made to establish this reason while fully respecting the participants’ rights.

If a participant in the intervention arm tells us that they would like to withdraw once they have completed Workshop 2, we will seek clarity on whether they would like to withdraw from both the intervention and the questionnaires, or whether they would like to withdraw from the intervention but continue to complete follow-up questionnaires. We will let them know that as Workshop 1 and Workshop 2 constitute minimum criteria for engagement, they can continue to respond to questionnaire follow-ups if they choose.

Should a participant withdraw from the study, unless they request that it is withdrawn, we will still use any previous data collected from that participant up to the point of withdrawal for the purposes of analysis. However, we will make no further attempt to contact them or collect data from them.

## 9.5 Lost to contact

Participants in the intervention arm may be considered lost to contact if they do not respond to requests to complete the intervention (e.g. they have completed one workshop). To enhance adherence to the intervention, participants in the intervention arm will be contacted by email, text message and phone call if they do not book onto or attend the workshops or individual phone call. Minimum adherence to the intervention will be defined as completion of both workshops. All randomised participants will be invited to complete the T2, T3 and T4 questionnaires irrespective of their level of adherence, unless they withdraw consent for further contact. The minimum adherence definition will be used for descriptive summaries of engagement and for prespecified per protocol and complier average causal effect (CACE) analyses, but not to determine who is followed up. If during follow up an intervention participant cannot be reached after successive attempted contacts to complete the questionnaire they will be considered lost to contact. Control arm participants will be defined as lost to contact in the follow up stage if after successive contact attempts they do not respond or complete the requested follow up questionnaire. If a participant is lost to contact, this will be recorded in the trial master file. Please refer to the statistical analysis plan for a comprehensive discussion of analytical implication of lost to contact participants.

# 10. Safety Reporting

## 10.1 Definitions

| Term | Definition |
| --- | --- |
| Adverse Event (AE) | Any untoward medical occurrence in a study participant. |
| Unexpected AE/SAE | An adverse event or serious adverse event which has not been pre-specificed as expected. |
| Serious Adverse Event (SAE) | An AE which is serious, defined as any untoward medical occurrence or effect that :   - Leads to death - Poses a threat to life* - Necessitates hospital admission or prolongation of existing hospitalisation and emergency care** - Cause lasting or significant impairment/disability or incapacity*** |
| Related AE/SAE | An AE or SAE which is related to a research procedure |
| Notable Event | An event of particular interest that does not necessarily meet the criteria for seriousness but requires expedited reporting as per the protocol. |

*The term life-threatening in the definition of a serious event refers to an event in which the participant is at risk of death at the time of the event; it does not refer to an event that hypothetically might cause death if it were more severe.

**Hospitalisation is defined as an inpatient admission, regardless of length of stay, even if the hospitalisation is a precautionary measure for continued observation. Hospitalisations for a pre-existing condition, that has not worsened or for an elective procedure do not constitute an SAE.

***Other important medical events that may not result in death, be life-threatening, or require hospitalisation may be considered a serious adverse event/experience when, based upon appropriate medical judgement, they may jeopardise the participant and may require medical or surgical intervention to prevent one of the outcomes listed in this definition.

In this study, an adverse event (AE) will be considered any untoward medical occurrence occurring in an ACTIVATE participant whether or not related to the research procedures or intervention. An adverse reaction (AR) will be defined as an untoward and unintended response in a participant that is potentially connected to any aspect of ACTIVATE. All study participants that have completed baseline measures will be followed up for adverse events unless they explicitly state they wish to withdraw from the study and receive no further contact. Adverse events will be recorded and reviewed by the CI and summary reports provided to the TSC for review of any possible patterns.

Foreseeable adverse events: as a significant proportion of medical students experience mental health problems such as depression, certain expected lower-level AEs will be reported to the TSC only at planned meetings, such as experiences of low mood, anxiety, or lower risk self-harming behaviours (e.g., superficial scratching) that do not need professional medical care. However, SAEs that could potentially occur—though less frequently—include mental health problems requiring hospitalisation or severe self-harm incidents (requiring medical treatment) and suicide. All adverse events will be recorded and shared with the CI. Potentially serious and unexpected adverse events will be reviewed by the CI who makes an assessment as to whether the event is defined as serious in consultation with definitions in the protocol. Advice and consultancy will be sought from the Chair of the TSC.

AEs will be monitored and recorded at T2 via the inclusion of a survey question, “Have you had any serious negative life experiences since you started the study? These might be social (e.g., problems with friends, family, peers, etc), personal (e.g., problems with a partner, housing, finances etc), psychological (e.g., occurrence or increase in symptoms of depression or anxiety, etc), medical (e.g., health-related ailments, self-harm or injury etc) or relate to other issues. If so, please describe what this was or these were. (Yes [please describe/open text box]; No).” We will also monitor them via items on the process questionnaires relating to moral and ethical concerns about the study, affective attitude towards the study (like/dislike) and experience of the study (degree of comfort experienced). Where participants report significant moral or ethical concerns, strongly disliking the study or feeling very uncomfortable, they will be contacted for more information. All participants who 1) agree with the AE statement and provide information which indicates or may indicate an AE in line with our definition, or 2) provide these responses on the process questionnaire will be contacted for further information and to determine whether the incident can be classed as an AE, AR, SAE or SAR.

If participants indicate during any other part of the study (e.g., during the workshops, the one-to-one call, interviews, or by contacting the study team) that an AE or SAE has occurred, they will similarly be contacted by the study team and asked to provide more information. The information sheet provided to participants includes contact details for individuals they should reach out to if they believe they have experienced any harm from participating in the study or if they want to file a formal complaint regarding how the study was conducted.

An SAE occurring to a research participant will be reported to the Chair of the Ethics Committee where in the opinion of the Chair of TSC that the event was: i) related, that is resulted from the administration of any of the research procedures and ii) unexpected – that is, the type of event is not listed in the protocol as an expected occurrence. The CI and the trial management team will review all SAEs, whether or not they are judged to be attributable to the trial or interventions. Based on this information, and in consultation with the Chair of the TSC, they will determine whether the participant should be withdrawn, and/or the trial should be suspended, stopped or continued. All AEs, ARs, SAEs and SARs will be fully documented and included in both the final trial report and published journal article.

# 11. Statistics

A detailed statistical analysis plan (SAP) approved by the independent Statistician can be seen in appendix 3.0. All analysis will be conducted in R (R Foundation for Statistical Computing, Vienna, Austria).

## 11.1 Sample size

In the study of Reboot in medical students [9], the within-group standardised mean change in Brief Resilience Scale (BRS) scores from baseline to 4-month follow up was large (Cohen’s d ≈ 0.90). Mean BRS increased from the “low” resilience band (1–2.99; T1 mean 2.74, SD 0.73) into the “normal” range (3–4.3; T4 mean 3.16, SD 0.72) on the 1–5 scale.

For the present trial, we powered the primary between-group comparison more conservatively for a standardised mean difference at 4-month follow up (T3) of d = 0.50, with 80% power at a two-sided α = 0.05. On the 1–5 BRS scale, *d* = 0.50 corresponds to a shift of roughly 9–11% of the possible range and would be expected to move a meaningful proportion of students from the low resilience band (BRS < 3) into the normal range. Effects of this magnitude are similar to those observed between clinically relevant groups in studies of Reboot with clinicians and are therefore considered potentially clinically important at the group level.

Power was estimated in R using the powerlmm package, under the planned mixed model for repeated measures. The primary contrast was the adjusted mean difference in BRS at T3 between the intervention (Thumos) and services as usual arms (Thumos minus control). Simulations assumed:

- Up to three post-baseline BRS observations per participant (T2, T3, T4); where more than one completion occurred within a time window, the response closest to the nominal time point was retained, preserving the repeated-measures structure;
- A within-participant intra-class correlation coefficient (ICC) of 0.30–0.50, representing a moderate correlation between repeated BRS scores on the same student, consistent with published test–retest data indicating that BRS is reasonably stable over time (doi:10.1080/10705500802222972);
- Variance ratio scenarios across time points using powerlmm defaults, specifying plausible ratios of between-participant to within-participant variance and allowing for heteroscedasticity over time and between arms (see powerlmm reference manual: http://cran.nexr.com/web/packages/powerlmm/powerlmm.pdf);
- Differential attrition of 40% in the control arm and 30% in the intervention arm up to T4, with monotone missingness. These rates were chosen to be conservative, based on studies of Reboot and meta-analytic data from occupational and mental health intervention trials that report similar or lower attrition (e.g. doi: 10.5271/sjweh.4173).

## 11.2 Statistical Analysis

Recruitment, retention, adherence and participant characteristics will be summarised using appropriate descriptive statistics. Minimum adherence to the intervention is defined as completion of two workshops. Adverse events/serious adverse events will be summarised descriptively by arm, with risk differences and 95% confidence intervals and narrative summaries by event type and seriousness.

The primary outcome (T3 BRS) will be analysed using linear mixed models for repeated measures (MMRM) with random intercepts for participant and fixed effects for treatment group, time, group by time interaction, baseline BRS and baseline covariates (age, gender, medical school). Adjustment for baseline BRS and covariates improves the power and plausibility of the analysis (e.g. make missing at random assumptions more credible by conditioning on variables related to outcome *and* dropout). Although the primary estimand is the adjusted mean difference in BRS at T3, we model T2/T4 BRS because BRS is measured repeatedly and the mixed model accounts for within participant correlation; it allows all post baseline data to contribute to estimation of the T3 contrast, improving precision relative to single time point models; and it gives valid inference when outcome data are missing at random given observed covariates and outcomes from studies of Reboot, which is realistic for the longitudinal design.

Continuous outcomes will be analysed on their original scales and results reported as adjusted mean differences with 95% confidence intervals, *p* values and standardised effect sizes (Cohen’s *d*). A two-sided of α = 0.05 is considered significant. Model assumptions will be explored and robust standard errors and/or alternative covariance structures will be considered as necessary.

Secondary count outcomes (sickness absence) will be modelled with Poisson models and binary outcomes (support access indicators) with mixed effects logistic regression, including random intercepts for participant and the same fixed effect structure as primary analyses. We will report incidence rate ratios and odds ratios with 95% confidence intervals and *p* values. Secondary analyses will be interpreted as supportive rather than confirmatory; to describe multiplicity we will report false discovery rate adjusted *q* values for secondary outcomes at T3-T4.

Patterns of data missingness will be described and explored using logistic regression models for missingness indicators. Sensitivity analyses will include multiple imputation under MAR, pattern mixture “worse than MAR” scenarios using fixed negative offsets to imputed BRS values, and a last post baseline carried forward analysis.

Adherence and subgroup effects will be explored in prespecified additional analyses, including per protocol analyses and complier average causal effect (CACE) estimation using randomisation as an instrument for full receipt of the intervention.

Interviews conducted as part of the process evaluation will be transcribed verbatim and analysed using Reflexive Thematic Analysis (RTA). Field notes and logs will also be analysed using RTA. RTA is a theoretically flexible approach for identifying, analysing and reporting patterns in qualitative data and is useful for obtaining detailed insights [36]. Internal reliability of qualitative data will be strengthened through the adoption of qualitative credibility strategies such as respondent validation of transcripts, peer debriefing and negative case analysis. Qualitative data will then be combined with quantitative data from the process questionnaires using a narrative discussion where the quantitative survey scores are presented alongside the themes produced by RTA [37]. In particular, we will follow the approach of Guetterman and Mitchel [38] who included a separate section following initial presentation of the quantitative (frequency) and qualitative (thematic) findings, labelled, ‘mixed-methods integration’. The purpose of this section will be to bring quantitative results together with relevant qualitative insights, to generate a nuanced and full picture of the dataset. This narrative discussion will be facilitated by the use of a side-by-side joint data display; a table where complementary and divergent qualitative and quantitative findings are included in columns to support the generation of complex insights [39].

# 12. Trial supervision

The trial has been carefully designed to ensure compliance with GCP and scientific

integrity. The implementation of the trial through its design and development is

managed by the CI and the co-applicants, in consultation with expert researchers and other expert collaborators from within and outside of the CI's institution.

A trial manager will manage the day to day running of the trial, reporting to the CI. A trial management group (TMG), a trial steering committee (TSC) will be established to oversee specific aspects of the trial as described in detail in the next section/s. A Data Monitoring and Ethics Committee (DMEC) will be formed if deemed necessary by the TSC, with the purpose of reviewing unblinded data. The DMEC will be formed separately and independently to the TSC.

## 13.1 Trial Steering Committee

The Trial Steering Committee (TSC) will provide guidance and supervision for the trial on behalf of the project's sponsor and funder and to ensure that it is conducted to the rigorous standards set out in the University of Manchester's [Code of Good Research Conduct](https://www.manchester.ac.uk/research/environment/governance/conduct/) and [policies and guidelines](https://www.manchester.ac.uk/research/environment/governance/policies-guidelines/). The specific role the TSC will provide is a regular review of the trial's progress including recruitment figures, data completeness, adherence to protocol treatment and follow-up, and safety data. The TSC will ensure the rights, safety and well-being of the participants are given the most important considerations and should prevail over the interests of science and society. It will ensure that all appropriate ethical approvals have been obtained and adhered to and approve any proposals for substantial protocol amendments. It will comprise seven independent members: a chairperson, one clinician, a statistician, a registered psychologist, and two experts by experience (medical students).

## 13.2 Data Monitoring and Ethics Committee

As a relatively small trial of N =220 participants of a preparatory intervention for medical students, ACTIVATE does not require a separate Data Monitoring and Ethics Committee (DMEC). A DMEC will be formed if deemed necessary by the TSC, with the purpose of reviewing unblinded data. The DMEC will be formed separately and independently to the TSC.

## 13.3 Stopping Guidelines

The trial will be paused in the event of two SAEs which are probably related to the study procedures. Information regarding these events will be collected by the CI and research team and passed on to the TSC Chair for consultation. If the TSC Chair deems the SAEs are related to the study procedures, the Chair of the Ethics Committee will be informed and consulted. The advice of the TSC and the Chair of the Ethics Committee will be sought and acted upon. The trial may be permanently discontinued at this point, or continued after review and with recommendations from the TSC and the Chair of the Ethics Committee addressed. The trial may be permanently discontinued in the event of three SAEs which are considered to be probably related to the trial procedures. The TSC Chair who will advise on whether to continue or discontinue the study, will make a recommendation to the sponsor.

All adverse events will be reported to the TSC who will review them. If the rate of AEs is >30% higher in one arm, the TSC may pause the trial to review further and consider whether the study is causing harmful effects. If after this review it is decided that the study is causing harmful events, it may be permanently discontinued.

The trial may also be discontinued if recruitment rates fall below 50% of the monthly target for 4 consecutive months. Recruitment rates will be monitored by the CI and research team and reported to the TSC. Should recruitment fall below this threshold, the CI will seek the advice of the TSC regarding whether to discontinue the study.

If the study is prematurely discontinued, active participants will be informed. In consultation with the TSC Chair, next steps will be agreed upon. It is most likely that data collection would continue but that participants would no longer receive the intervention.

## 13.4 Trial Management Group

A trial management group (TMG) will meet regularly; its membership will include the

investigators, collaborators and PPI medical students. The TMG will be chaired by the CI and is responsible for the day to day running of the trial, ensuring good communication between the members of the research team, overseeing recruitment progress, intervention completion, adverse events and reviewing overall trial progress against milestones. It will oversee the preparation of reports to the TSC (and DMEC if required).

# 14. Data handling and record keeping

Participant contact details, demographic information and quantitative data will be collected via the University of Manchester access to the Qualtrics platform. Qualitative data will be collected via the University of Manchester access to the Microsoft Teams platform, using the recording and transcription functions. Qualitative interviews will be conducted by the University of Manchester-employed researcher. At the end of the study, all quantitative data will be downloaded from Qualtrics and deleted from their system. Qualitative videos will be downloaded from Teams following each interview, and uploaded to secure University of Manchester systems. They will then be deleted from Teams. Once the anonymised transcript for each video has been completed, the video will be deleted from the University of Manchester system, and no copy of the video will exist.

All data will be electronic, there will be no hard copies. Electronic identifiable data will be stored on secure, password protected University drives, in encrypted files. Data will be pseudonymised; a code will be used to match personal identifiable data and research data, which only the University of Manchester team has access to. Personal data will not be shared outside of the University of Manchester research team. Personal identifiable data will be stored for up to 5 years following recruitment of the final participant. This is to enable potential follow-up studies to be conducted, dependent on further grant funding being awarded to conduct these studies. However, the personal information stored will only be used to contact participants to invite them to future studies, not as part of the research data set in and of itself.

# 15. Data access and quality assurance

All data will be kept secure at all times and maintained in accordance with the requirements of the Data Protection Act, and archived according to clinical trial GCP regulations. Personal data will be stored in encrypted, password protected formats. Identifiable personal data will be stored separately to other study data to help maintain confidentiality. Only the University of Manchester research team members will have access to the data. No personal or identifiable data will be transferred. Only anonymised datasets will be shared with collaborators outside of the University of Manchester during the project, for the purposes of analysis.

The full dataset will be retained for up to 5 years in first instance, to enable possible follow-up studies to be conducted, should additional funding be gained. It will then be deleted, and only anonymised datasets will be retained and uploaded to the University of Manchester data archive/repository. Consent forms will also be retained during this time and destroyed at the end of this time.

The anonymised datasets (the anonymised quantitative dataset and the anonymised qualitative transcripts) will be uploaded after the project has finished to the University of Manchester archive/data repository. Participants are asked to give permission on the consent form for the anonymised dataset to be uploaded to a repository. Providing this permission is necessary to take part in the study. To avoid any issues with data sharing, only anonymised data will be uploaded to a repository. The anonymised datasets created during the present study will be available in a public repository, hosted at the University of Manchester research - ([https://figshare.manchester.ac.uk](https://figshare.manchester.ac.uk/)/).

Relevant parties (e.g., ethics committee, TSC) will be notified of any modifications to the protocols or data management plan in a timely manner.

# 16. Publication

The trial protocol will shortly be submitted for publication. Planned main publication in high-impact peer-reviewed journal with paper analysing the primary outcomes of the effectiveness of Thumos in increasing psychological resilience compared to SAU at 4-month follow-up. This paper will also report on the secondary outcomes of the effectiveness of Thumos compared to SAU at post-intervention, 4- and 9-month follow-ups in the endpoints of confidence, burnout, depression, mental health and self-reported sickness absence.

# 17. Finance

This trial is funded by The MPS Foundation. The Funder reviewed and approved the content of the protocol, but does not have a role in data collection, management, analysis, or interpretation; nor in the writing of the final report or decision to submit the report. The views expressed in this publication are those of the authors and not necessarily those of the MPS Foundation.

# 18. Ethics approval & regulatory compliance

The project was approved on 20/12/2024, by the University of Manchester Research Ethics Committee 1 (2nd Floor, Christie Building, The University of Manchester, Oxford Road, Manchester, M139PL, United Kingdom; +44 (0)161 306 6000; urec1@manchester.ac.uk), ref: 2024-21589-38791, prior to study commencement.

# 19. Medical Student Involvement

At least four medical students will be involved; at least two will join the TSC to provide independent oversight to study management, and at least two will join the trial management committee to provide feedback on study materials and delivery.

# 22. Indemnity / Compensation / Insurance

The University of Manchester has in place research study insurance against liabilities for which it may be legally liable and this cover includes any such liabilities arising out of this study.

# 23. References

1.	Haakenstad, A., et al., *Measuring the availability of human resources for health and its relationship to universal health coverage for 204 countries and territories from 1990 to 2019: a systematic analysis for the Global Burden of Disease Study 2019.* The Lancet, 2022. **399**(10341): p. 2129-2154.

2.	The Health Foundation, The Kingsfund, and Nuffield Trust. *Closing the gap: Key areas for action on the health and care workforce*. 2019 [cited 2019 20th September]; Available from: <https://www.kingsfund.org.uk/sites/default/files/2019-03/closing-the-gap-health-care-workforce-overview_0.pdf>

3.	Dall'Ora, C., et al., *Nurse staffing levels and patient outcomes: a systematic review of longitudinal studies.* International Journal of Nursing Studies, 2022: p. 104311.

4.	Demerouti, E. and A.B. Bakker, *The Oldenburg Burnout Inventory: A good alternative to measure burnout and engagement.* Handbook of stress and burnout in health care, 2008. **65**(7): p. 1-25.

5.	Toppinen-Tanner, S., et al., *Burnout as a predictor of medically certified sick-leave absences and their diagnosed causes.* Behavioral medicine, 2005. **31**(1): p. 18-32.

6.	Bae, S.H., et al., *Predictors of actual turnover among nurses working in Korean hospitals: A nationwide longitudinal survey study.* Journal of Nursing Management, 2021. **29**(7): p. 2102-2114.

7.	Hodkinson, A., et al., *Associations of physician burnout with career engagement and quality of patient care: systematic review and meta-analysis.* BMJ, 2022. **378**.

8.	Vogt, K.S., et al., *Critical care nursing workforce in crisis: A discussion paper examining contributing factors, the impact of the COVID‐19 pandemic and potential solutions.* Journal of Clinical Nursing, 2023.

9.	Johnson, J., et al., *Reboot coaching programme: a mixed-methods evaluation assessing resilience, confidence, burnout and depression in medical students.* Scottish Medical Journal, 2023: p. 00369330231213981.

10.	Rotenstein, L.S., et al., *Prevalence of depression, depressive symptoms, and suicidal ideation among medical students: a systematic review and meta-analysis.* Jama, 2016. **316**(21): p. 2214-2236.

11.	Puthran, R., et al., *Prevalence of depression amongst medical students: A meta‐analysis.* Medical education, 2016. **50**(4): p. 456-468.

12.	Dyrbye, L.N., et al., *Burnout and suicidal ideation among US medical students.* Annals of internal medicine, 2008. **149**(5): p. 334-341.

13.	British Medical Association. *Four in ten junior doctors plan to leave the NHS as soon as they can find another job, BMA council chair reveals in New Year's message*. 2022 [cited 2023 23rd June]; Available from: <https://www.bma.org.uk/bma-media-centre/four-in-ten-junior-doctors-plan-to-leave-the-nhs-as-soon-as-they-can-find-another-job-bma-council-chair-reveals-in-new-years-message-to-the-country>.

14.	Tolksdorf, K.H., U. Tischler, and K. Heinrichs, *Correlates of turnover intention among nursing staff in the COVID-19 pandemic: a systematic review.* BMC Nursing, 2022. **21**(1): p. 174.

15.	Hill, M.R., S. Goicochea, and L.J. Merlo, *In their own words: stressors facing medical students in the millennial generation.* Medical education online, 2018. **23**(1): p. 1530558.

16.	Hu, K.S., J.T. Chibnall, and S.J. Slavin, *Maladaptive perfectionism, impostorism, and cognitive distortions: Threats to the mental health of pre-clinical medical students.* Academic Psychiatry, 2019. **43**(4): p. 381-385.

17.	Weurlander, M., et al., *How do medical and nursing students experience emotional challenges during clinical placements?* International journal of medical education, 2018. **9**: p. 74.

18.	Nteveros, A., et al., *Burnout among medical students in Cyprus: A cross-sectional study.* PLoS One, 2020. **15**(11): p. e0241335.

19.	Witt, K., et al., *Effectiveness of universal programmes for the prevention of suicidal ideation, behaviour and mental ill health in medical students: a systematic review and meta-analysis.* BMJ Ment Health, 2019. **22**(2): p. 84-90.

20.	Johnson, J., et al., *Can we prepare healthcare professionals and students for involvement in stressful healthcare events? A mixed-methods evaluation of a resilience training intervention.* BMC Health Serv Res, 2020. **20**(1): p. 1094.

21.	Sperling, E.L., et al., *The effect of resilience training on resilience and stress in medical students: a systematic review and meta-analysis.* BMC Medical Education, 2025.

22.	Johnson, J., Simms-Ellis, R., Janes, G., Mills, T., Budworth, B., Atkinson, L. & Harrison, R., *Can we prepare healthcare professionals for involvement in adverse events? Feasibility study of a resilience training intervention.* BMC Health Services Research, 2020. **20**: p. 1094.

23.	Vogt, K., et al., *Can the Reboot coaching programme support critical care nurses in coping with stressful clinical events? A mixed-methods evaluation assessing resilience, burnout, depression and turnover intentions.* BMC Health Services Research, 2024. **In press**.

24.	Al‐Ghunaim, T., et al., *Evaluation of the reboot coaching workshops among urology trainees: A mixed method approach.* BJUI compass, 2023. **4**(5): p. 533-542.

25.	Chew‐Graham, C.A., A. Rogers, and N. Yassin, *‘I wouldn't want it on my CV or their records': medical students' experiences of help‐seeking for mental health problems.* Medical education, 2003. **37**(10): p. 873-880.

26.	Dyrbye, L.N., et al., *The impact of stigma and personal experiences on the help-seeking behaviors of medical students with burnout.* Academic medicine, 2015. **90**(7): p. 961-969.

27.	Janes, G., et al., *Multiple meanings of resilience: Health professionals' experiences of a dual element training intervention designed to help them prepare for coping with error.* Journal of Evaluation in Clinical Practice, 2021. **28**: p. 315-323.

28.	Vogt, K., et al., *Study protocol for the online adaptation and evaluation of the ‘Reboot’(Recovery-boosting) coaching programme, to prepare critical care nurses for, and aid recovery after, stressful clinical events.* Pilot and Feasibility Studies, 2022. **8**(1): p. 63.

29.	Smith, B.W., et al., *The brief resilience scale: assessing the ability to bounce back.* International journal of behavioral medicine, 2008. **15**(3): p. 194-200.

30.	Löwe, B., et al., *Monitoring depression treatment outcomes with the patient health questionnaire-9.* Medical care, 2004. **42**(12): p. 1194-1201.

31.	Keetharuth, A.D., et al., *Estimating a preference-based index for mental health from the recovering quality of life measure: valuation of recovering quality of life utility index.* Value in Health, 2021. **24**(2): p. 281-290.

32.	Yoon, S., et al., *Usefulness of the Patient Health Questionnaire-9 for Korean medical students.* Academic Psychiatry, 2014. **38**(6): p. 661-667.

33.	Cameron, I.M., et al., *Psychometric comparison of PHQ-9 and HADS for measuring depression severity in primary care.* The British Journal of General Practice, 2008. **58**(546): p. 32.

34.	Skivington, K., et al., *A new framework for developing and evaluating complex interventions: update of Medical Research Council guidance.* bmj, 2021. **374**.

35.	Sekhon, M., M. Cartwright, and J.J. Francis, *Development of a theory-informed questionnaire to assess the acceptability of healthcare interventions.* BMC health services research, 2022. **22**(1): p. 279.

36.	Braun, V. and V. Clarke, *Reflecting on reflexive thematic analysis.* Qualitative research in sport, exercise and health, 2019. **11**(4): p. 589-597.

37.	Kendal, S., et al., *Implementing and evaluating patient‐focused safety technology on adult acute mental health wards.* Journal of Psychiatric and Mental Health Nursing, 2024. **31**(5): p. 742-754.

38.	Guetterman, T.C. and N. Mitchell, *The role of leadership and culture in creating meaningful assessment: A mixed methods case study.* Innovative Higher Education, 2016. **41**(1): p. 43-57.

39.	Walton, J.B., et al., *Navigating intersecting roads in a mixed methods case study: A dissertation journey.* Journal of Mixed Methods Research, 2020. **14**(4): p. 436-455.

# Appendices

# Appendix 1.0

## 1.0. List of eligible UK medical schools

University of Southampton School of Medicine

Hull York Medical School (HYMS)

University of Leicester Medical School

Brighton and Sussex Medical School

University College London Medical School (UCL)

King’s College London GKT School of Medical Education (KCL)

City St George’s, University of London

University of Birmingham School of Medical Sciences

University of Dundee School of Medicine

University of Liverpool School of Medicine

University of Sheffield medical school

University of Manchester medical school

Anglia Ruskin University School of Medicine

Aston University Medical School

Brunel University Medical School

Cardiff University School of Medicine

Edge Hill University Medical School

Imperial College London Faculty of Medicine

Keele University School of Medicine

Kent and Medway Medical School

Lancaster University Medical School

London School of Hygiene and Tropical Medicine (LSHTM)

Newcastle University School of Medical Education

North Wales Medical School, Bangor University

Norwich Medical School, University of East Anglia

Pears Cumbria School of Medicine

Peninsula Medical School Plymouth University

Queen Mary, University of London School of Medicine

Queen’s University Belfast School of Medicine

St Mary’s University, Twickenham School of Medicine

University of Cambridge, School of Clinical Medicine

University of Oxford, Medical Sciences

University of Edinburgh, Medical School

University of Glasgow, School of Medicine

University of Bristol, Medical School

University of Buckingham Medical School

University of Lancashire School of Medicine

Swansea University Medical School

Three Counties Medical School, University of Worcester

Ulster University, School of Medicine

University of Aberdeen School of Medicine

University of Chester Medical School

University of Exeter Medical School

University of Greater Manchester, Medical School

University of Leeds School of Medicine

University of Lincoln Medical School

University of Nottingham School of Medicine

University of St Andrews School of Medicine

University of Sunderland School of Medicine

University of Surrey School of Medicine

University of Warwick Medical School

# Appendix 2.0

## 2.0 Participant Information Sheet and Consent Form

**V3 11-03-2025 – medical students**

**A randomised controlled trial evaluating whether a CBT-based group and one-to-one supportive intervention can improve burnout and mental health in medical students**

**Participant Information Sheet (PIS) for medical students**

You are being invited to take part in a research study evaluating an intervention in medical students. Before you decide whether to take part, it is important for you to understand why the research is being conducted and what it will involve. Please take time to read the following information carefully before deciding whether to take part, and discuss it with others if you wish. Please ask if there is anything that is not clear or if you would like more information. Thank you for taking the time to read this.

**About the research**

**Who will conduct the research?**

Dr Judith Johnson and Lucy Pointon who are based in the Division of Nursing, Midwifery and Social Work at The University of Manchester. A researcher from the University of Sheffield is also participating.

**What is the purpose of the research?**

The study aims to evaluate a supportive programme (Thumos) designed to prepare medical students for stressful events faced at work.

**Am I suitable to take part?**

We’d love to hear from you if you are a medical student in a year involving clinical placements. This is usually year 4 or year 5 for most medical schools, but can vary.

**Will the outcomes of the research be published?**

We will publish our findings in peer-reviewed journal articles. We might also promote the findings via social media.

**Who has reviewed the research project?**

The project has been reviewed by The University of Manchester Research Ethics Committee.

**Who is funding the research project?**

The Medical Protection Society Foundation.

**What would I be asked to do if I took part?**

If you decide to take part in the research study, you will be asked to complete a consent form and some baseline questionnaires, which will ask for demographic details (e.g., age, gender) and your thoughts and perceptions relating to your mental health and psychological wellbeing. They will take about 20m altogether. You will then be randomised to either receive the Thumos intervention, or to the control group, where you will not receive the intervention. Half of participants will be randomly allocated to receive the intervention; the other half will not be allocated to receive the intervention. This decision is completely random and is not affected by any of your responses to the questionnaires. In either group, you can continue to access your usual wellbeing or mental health support services as usual.

If you are randomly allocated to receive the intervention, we will ask you to:

- Engage in the Thumos programme, combining 2 small group online workshops and a personalised coaching call. The workshops will each last up to 2 hours and the coaching call will last up to an hour (5 hours total).
- Complete further online questionnaires after Thumos has finished, first on completion of the coaching call, then 4 months after starting the study and finally 9 months after starting the study. This will take around 20m at each time.
- Over the course of the study, some workshops will be observed by a researcher who will join the call, and we will request permission to video record some of the coaching calls. This is so that we can observe the therapist and identify how closely they are following the intervention plan. Any recordings will be destroyed once they have been watched by the research team and scored for how closely they are following the intervention plan.
- We will also ask your permission to retain your contact details, so we could contact you again in 2 and 5 years’ time, to ask you to complete further questionnaires.
- A subgroup of participants will be asked to answer additional questions as part of a qualitative interview at around 4 months after the first questionnaire, discussing your experiences of Thumos. These will last up to 30 minutes. These interviews will be conducted on Teams and video recorded.

If you are randomly allocated not to receive the intervention, we will ask you to:

- Complete further online questionnaires 4-6 weeks after the first questionnaire, again 4 months after starting the study and then finally 9 months after starting the study. This will take around 20m at each time.
- We will also ask your permission to retain your contact details, so we could contact you again in 2 and 5 years’ time, to ask you to complete further questionnaires.

**Will I be compensated for taking part?**

You will not be compensated for completing the first set of questionnaires. However, for each of the remaining three follow-up questionnaires, you will receive a £10 shopping voucher. For participants who take part in the qualitative interviews, you will receive a further £10 shopping voucher.

**What happens if I do not want to take part or if I change my mind?**

It is up to you to decide whether or not to take part. If you do decide to take part you will be given this information sheet to keep and will be asked to provide your name and tick a box to confirm consent on Qualtrics survey software online. If you decide to take part you are still free to withdraw at any time without giving a reason and without detriment to yourself. However, it will not be possible to remove your data from the project once it has been anonymised as we will not be able to identify your specific data. This does not affect your data protection rights. If you decide not to take part you do not need to do anything further.

For participants who are invited to take part in the qualitative interviews, these will be conducted on Teams and will be video recorded. We need this recording to be able to analyse the data, so we need to do this for you to take part in the interview. If should be comfortable with the recording process at all times and are free to stop recording at any time, should you feel uncomfortable.

**Data Protection and Confidentiality**

**What information will you collect about me?**

In order to participate in this research project we will need to collect information that could identify you, called “personal identifiable information”. Specifically we will need to collect:

- Your name
- Contact details and address (for posting the paper workbook to you)
- Gender
- Ethnicity
- Age
- Region
- Your year of medical school
- Whether you are in a year involving clinical placements
- Whether you have begun or completed one or more clinical placements
- Which medical school you attend
- For participants who take part in the qualitative interviews, these will be conducted on Teams. We will ask permission to video record the discussion.

**Under what legal basis are you collecting this information?**

We are collecting and storing this personal identifiable information in accordance with UK data protection law which protect your rights. These state that we must have a legal basis (specific reason) for collecting your data. For this study, the specific reason is that it is “a public interest task” and “a process necessary for research purposes”.

**What are my rights in relation to the information you will collect about me?**

You have a number of rights under data protection law regarding your personal information. For example you can request a copy of the information we hold about you, including video recordings.

If you would like to know more about your different rights or the way we use your personal information to ensure we follow the law, please consult our [Privacy Notice for Research](http://documents.manchester.ac.uk/display.aspx?DocID=37095).

**Will my participation in the study be confidential and my personal identifiable information be protected?**

In accordance with data protection law, The University of Manchester is the Data Controller for this project. This means that we are responsible for making sure your personal information is kept secure, confidential and used only in the way you have been told it will be used. All researchers are trained with this in mind, and your data will be looked after in the following way:

- For the questionnaire data, we have taken measures to ensure confidentiality, such as providing participants with an assigned ID number only known to the research team (known as pseudonymised).
- We will ask your permission to retain your data for up to 5 years after the end of the study. This is to enable us to contact you again in 2 and 5 years to conduct further follow up studies, should we gain additional funding for this. If you decline to provide permission, your identifiable data will be deleted at the end of the study, which will be 2 months after the final participant has provided their follow-up data. You can indicate your choice on the consent form. If you provide consent for this, your details will be safely stored on UoM servers in a digital folder only accessible to the study team and used only for the purposes described above.
- Data will be held on secure University of Manchester OneDrive servers. Only completely anonymised data will be shared with any other organisations (e.g., anonymous quantitative data files; anonymised transcripts, where all identifying details have been changed). This data will be shared for the purposes of statistical analysis.
- Identifiable and anonymised data will be stored for up to 5 years on University of Manchester secure OneDrive servers.
- At the end of the project we will deposit a fully anonymised dataset [e.g. including de-identified interview transcripts] in an open data repository where it will be permanently stored. We will use the University of Manchester Library. Researchers at other institutions and others can access the anonymised data directly from the repository and use it for further research or to check our analysis and results.
- For participants who take part in online qualitative interviews, the recordings will be used to create transcripts. This will be done initially by the Teams transcription software and then will be checked and corrected by a study team researcher.
  - Personal identifiable information will be removed in the final transcript
  - The recordings will be destroyed following transcription
  - Your participation in the qualitative interview will be recorded in Teams and your personal data will be processed by Microsoft. This may mean that your personal data is transferred to a country outside of the European Economic Area, some of which have not yet been determined by the United Kingdom to have an adequate level of data protection. Appropriate legal mechanisms to ensure these transfers are compliant with the Data Protection Act 2018 and the UK General Data Protection Regulation are in place. The recordings will be removed from the above third party platform and stored on University of Manchester managed file storage as soon as possible following the completion of data collection.

The study team at The University of Manchester will have access to your personal information and they will anonymise it as soon as possible. Your name and any other identifying information will be removed and replaced with a random ID number. The research team will have access to the key that links this ID number to your personal information. Your consent form will be retained for 5 years on secure University of Manchester OneDrive servers for audit purposes.

So that we can provide the shopping/Amazon voucher as a thank you for your time, your full name and email address will be shared with our Finance department who will send the voucher to you. Your full name and email address will be securely retained by Finance for a period of up to 7 years for audit purposes only and then destroyed. It will not be used for them for any other purpose.

If, during the study, you disclose information about patient safety hazards or risks you are facing on placement, we will advise you that you have a responsibility to raise these issues via your clinical supervisor and we will support you to do this. If you refuse to raise issues of concern via the normal routes, and it is likely that disclosure will prevent harm to others in future*,* we may need to contact the relevant NHS trust directly and communicate the risks/concerns directly to the trust’s local patient safety team on your behalf. We will maintain confidentiality wherever possible but break confidentiality and disclose your identity if necessary.

If, during the study, you disclose that you have experienced a serious negative event while participating in the study, we will contact you to request more information about the event, to try and understand if it is related to the study procedures.

Please also note that individuals from The University of Manchester or regulatory authorities may need to look at the data collected for this study to make sure the project is being carried out as planned. This may involve looking at identifiable data. All individuals involved in auditing and monitoring the study will have a strict duty of confidentiality to you as a research participant.

**What if I have a complaint?**

**Contact details for complaints**

If you have a complaint that you wish to direct to members of the research team, please contact: **DR JUDITH JOHNSON,** [**JUDITH.JOHNSON@MANCHESTER.AC.UK**](mailto:JUDITH.JOHNSON@MANCHESTER.AC.UK)**.**

**If you wish to make a formal complaint to someone independent of the research team or if you are not satisfied with the response you have gained from the researchers in the first instance, then please contact:**

The Research Ethics Manager, Research Office, Christie Building, The University of Manchester, Oxford Road, Manchester, M13 9PL, by emailing: [research.complaints@manchester.ac.uk](mailto:research.complaints@manchester.ac.uk)  or by telephoning 0161 306 8089.

If you wish to contact us about your data protection rights, please email [dataprotection@manchester.ac.uk](mailto:dataprotection@manchester.ac.uk) or write to The Information Governance Office, Christie Building, The University of Manchester, Oxford Road, M13 9PL at the University and we will guide you through the process of exercising your rights.

You also have a right to complain to the [Information Commissioner’s Office](https://ico.org.uk/concerns) about complaints relating to your personal identifiable information Tel 0303 123 1113

**Contact Details**

If you have any queries about the study or if you are interested in taking part, then please contact the researchers:

Lucy Pointon: [lucy.pointon@manchester.ac.uk](mailto:lucy.pointon@manchester.ac.uk) or [ThumosTrial@manchester.ac.uk](mailto:ThumosTrial@manchester.ac.uk)

Dr Judith Johnson: [Judith.johnson@manchester.ac.uk](mailto:Judith.johnson@manchester.ac.uk)

**V3 11-03-2025 – medical students**

**A randomised controlled trial evaluating whether a CBT-based group and one-to-one supportive intervention can improve burnout and mental health in medical students**

**Consent Form for medical students**

If you are happy to participate please tick each box and then enter your name below.

|  | **Activities** | Tick to indicate agreement |
| --- | --- | --- |
| 1 | I confirm that I have read the attached information sheet (**Version 3, Date 11-03-2025**) for the above study and have had the opportunity to consider the information and ask questions and had these answered satisfactorily. |  |
| 2 | I understand that my participation in the study is voluntary and that I am free to withdraw at any time without giving a reason and without detriment to myself. I understand that it will not be possible to remove my data from the project once it has been anonymised and forms part of the data set.  I agree to take part on this basis. |  |
| 3 | If I am invited to a qualitative interview and choose to take part in this, I agree to the **interviews** being **video recorded** |  |
| 4 | I agree that any data collected may be included in anonymous form in **publications/conference presentations.** |  |
| 5 | I agree that any research publications can include direct quotes of my responses in **anonymous** format. |  |
| 6 | I understand that a fully anonymised dataset will be deposited in an open data repository at the end of the project. |  |
| 7 | I understand that data collected during the study may be looked at by individuals from The University of Manchester or regulatory authorities, where it is relevant to my taking part in this research. I give permission for these individuals to have access to my data. |  |
| 8 | I understand that my full name and email address will be passed to the University's Finance team for the sole purpose of sending me the **shopping/Amazon voucher.** |  |
| 9 | I understand that there may be instances where during the course of the research information is revealed which means the researchers will be obliged to break confidentiality and this has been explained in more detail in the information sheet. |  |
| 10 | I understand that if I disclose evidence of patient safety risks in my clinical placement, I will be encouraged to report these to the relevant authorities. If I choose not to and it is likely that disclosure will prevent harm to others in future, I understand that the research team may need to raise these risks with the relevant NHS Trust, and that they will make every effort to maintain my confidentiality, but may need to disclose this and will do so if necessary. |  |
| 11 | I understand that some workshops may be observed by a researcher and that my therapist may request permission to video record our coaching call. I understand that this is for the purpose of understanding how closely the therapist is following the intervention protocol, and provide my permission for these observations/recordings. |  |
| 12 | I understand that if I disclose that a serious negative event has occurred to be during the course of the study, the research team will contact me to request more information about this. |  |
| 13 | I agree to take part in this study. |  |

**The following activities are optional, you may participate in the research without agreeing to the following:**

| 14 | I give permission for my contact details to be retained for up to 5 years to enable potential follow up studies to be conducted, regarding the intervention. I agree that the researchers may contact me in future about these projects, but do not give permission to be contacted about any other research projects. |  |
| --- | --- | --- |
| 15 | I agree that the researchers may retain my contact details in order to provide me with a summary of the findings for this study. |  |

**Data Protection**

**The personal information we collect and use to conduct this research will be processed in accordance with UK data protection law as explained in the Participant Information Sheet and the** [**Privacy Notice for Research Participants**](http://documents.manchester.ac.uk/display.aspx?DocID=37095)**.**

By providing my full name and email address below, I declare that I have provided accurate identity details.

________________________	________________________

Name of Participant	Email address	Date

This consent form will be retained on the Qualtrics platform. You can request a copy by contacting the research team (Dr Judith Johnson, [Judith.johnson@manchester.ac.uk](mailto:Judith.johnson@manchester.ac.uk); Lucy Pointon, [lucy.pointon@manchester.ac.uk](mailto:lucy.pointon@manchester.ac.uk))

# Appendix 3.0

**ACTIVATE Statistical Analysis Plan (SAP)**

**Sec 1: Administrative Information**

| **Version (date)** | **1.0 (13/08/26)** | | |
| --- | --- | --- | --- |
| **Project title** | A randomised controlled evaluation of Thumos: a cognitive-behavioural intervention for medical students | | |
| **Acronym** | ACTIVATE | | |
| **SAP author** | Dr Luke Budworth | | |
| **ISRCTN** | 12744098 | | |
| **REC reference** | 2024-21589-38791 | | |
| **Funded by** | The Medical Protection Society (MPS) Foundation | | |
| **Chief Investigator** | Dr Judith Johnson | | |
| **Sponsor** | University of Manchester | | |
|  |  | |  |
| **Approvals** | **Name** | **Signature** | **Date** |
| **Chief Investigator** | Dr Judith Johnson |  |  |
| **Trial Statistician** | Dr Luke Budworth |  |  |
| **TSC Independent Statistician** | Prof Mona Kanaan |  |  |

### ***Overview***

This Statistical Analysis Plan sets out how the primary results paper from the ACTIVATE trial will be analysed and reported. Any related papers, including further exploratory work, are not bound by it but will follow the same principles.

We have tried to pre-specify as much as we can, including a justified set of sensitivity analyses to test the robustness of the primary result. Not everything can be anticipated, so the plan does not rule out exploratory analysis or sensible judgement in analysis and reporting. Where it does specify something, we will follow it as closely as we can.

The SAP follows Sheffield CTRU SOP ST001, "The Statistical Analysis Plan" (02/07/2025), and the Gamble et al. (2017) guidelines on SAP content. A completed reporting checklist is in Section 14.

## **Sec 2: Background & aims**

### ***Background***

Healthcare systems internationally are experiencing a workforce crisis, with a current global shortage of six million physicians [[1](https://ukc-word-edit.officeapps.live.com/we/wordeditorframe.aspx?ui=en-GB&rs=en-US&wopisrc=https%3A%2F%2Flivemanchesterac-my.sharepoint.com%2Fpersonal%2Fjudith_johnson_manchester_ac_uk%2F_vti_bin%2Fwopi.ashx%2Ffiles%2Fe7ceb6b0a8b24c46b9fca48e0c21f06a&wdenableroaming=1&mscc=1&wdodb=1&hid=15D032A2-003B-2001-C43C-45F56ADF876D.0&uih=sharepointcom&wdlcid=en-GB&jsapi=1&jsapiver=v2&corrid=fb1b269f-e890-2117-5f8a-b0b5636b4890&usid=fb1b269f-e890-2117-5f8a-b0b5636b4890&newsession=1&sftc=1&hfto=1787156527793.3&uihit=docaspx&muv=1&ats=PairwiseBroker&cac=1&sams=1&mtf=1&sfp=1&sdp=1&hch=1&hwfh=1&dchat=1&sc=%7B%22pmo%22%3A%22https%3A%2F%2Flivemanchesterac-my.sharepoint.com%22%2C%22pmshare%22%3Atrue%7D&wdorigin=ItemsView&ctp=LeastProtected&rct=Normal&wdhostclicktime=1787156527792&afdflight=83&csiro=1&instantedit=1&wopicomplete=1&wdredirectionreason=Unified_SingleFlush#_ENREF_1)]. While poor workforce planning has contributed to this problem, high turnover has been identified as the main contributing factor [[2](https://ukc-word-edit.officeapps.live.com/we/wordeditorframe.aspx?ui=en-GB&rs=en-US&wopisrc=https%3A%2F%2Flivemanchesterac-my.sharepoint.com%2Fpersonal%2Fjudith_johnson_manchester_ac_uk%2F_vti_bin%2Fwopi.ashx%2Ffiles%2Fe7ceb6b0a8b24c46b9fca48e0c21f06a&wdenableroaming=1&mscc=1&wdodb=1&hid=15D032A2-003B-2001-C43C-45F56ADF876D.0&uih=sharepointcom&wdlcid=en-GB&jsapi=1&jsapiver=v2&corrid=fb1b269f-e890-2117-5f8a-b0b5636b4890&usid=fb1b269f-e890-2117-5f8a-b0b5636b4890&newsession=1&sftc=1&hfto=1787156527793.3&uihit=docaspx&muv=1&ats=PairwiseBroker&cac=1&sams=1&mtf=1&sfp=1&sdp=1&hch=1&hwfh=1&dchat=1&sc=%7B%22pmo%22%3A%22https%3A%2F%2Flivemanchesterac-my.sharepoint.com%22%2C%22pmshare%22%3Atrue%7D&wdorigin=ItemsView&ctp=LeastProtected&rct=Normal&wdhostclicktime=1787156527792&afdflight=83&csiro=1&instantedit=1&wopicomplete=1&wdredirectionreason=Unified_SingleFlush#_ENREF_2)]. Inadequate staffing is linked with negative patient outcomes including extended hospital stays and mortality [[3](https://ukc-word-edit.officeapps.live.com/we/wordeditorframe.aspx?ui=en-GB&rs=en-US&wopisrc=https%3A%2F%2Flivemanchesterac-my.sharepoint.com%2Fpersonal%2Fjudith_johnson_manchester_ac_uk%2F_vti_bin%2Fwopi.ashx%2Ffiles%2Fe7ceb6b0a8b24c46b9fca48e0c21f06a&wdenableroaming=1&mscc=1&wdodb=1&hid=15D032A2-003B-2001-C43C-45F56ADF876D.0&uih=sharepointcom&wdlcid=en-GB&jsapi=1&jsapiver=v2&corrid=fb1b269f-e890-2117-5f8a-b0b5636b4890&usid=fb1b269f-e890-2117-5f8a-b0b5636b4890&newsession=1&sftc=1&hfto=1787156527793.3&uihit=docaspx&muv=1&ats=PairwiseBroker&cac=1&sams=1&mtf=1&sfp=1&sdp=1&hch=1&hwfh=1&dchat=1&sc=%7B%22pmo%22%3A%22https%3A%2F%2Flivemanchesterac-my.sharepoint.com%22%2C%22pmshare%22%3Atrue%7D&wdorigin=ItemsView&ctp=LeastProtected&rct=Normal&wdhostclicktime=1787156527792&afdflight=83&csiro=1&instantedit=1&wopicomplete=1&wdredirectionreason=Unified_SingleFlush#_ENREF_3)]. It also creates a vicious cycle, whereby existing staff become overworked and burnt-out, a syndrome characterised by exhaustion and disengagement [[4](https://ukc-word-edit.officeapps.live.com/we/wordeditorframe.aspx?ui=en-GB&rs=en-US&wopisrc=https%3A%2F%2Flivemanchesterac-my.sharepoint.com%2Fpersonal%2Fjudith_johnson_manchester_ac_uk%2F_vti_bin%2Fwopi.ashx%2Ffiles%2Fe7ceb6b0a8b24c46b9fca48e0c21f06a&wdenableroaming=1&mscc=1&wdodb=1&hid=15D032A2-003B-2001-C43C-45F56ADF876D.0&uih=sharepointcom&wdlcid=en-GB&jsapi=1&jsapiver=v2&corrid=fb1b269f-e890-2117-5f8a-b0b5636b4890&usid=fb1b269f-e890-2117-5f8a-b0b5636b4890&newsession=1&sftc=1&hfto=1787156527793.3&uihit=docaspx&muv=1&ats=PairwiseBroker&cac=1&sams=1&mtf=1&sfp=1&sdp=1&hch=1&hwfh=1&dchat=1&sc=%7B%22pmo%22%3A%22https%3A%2F%2Flivemanchesterac-my.sharepoint.com%22%2C%22pmshare%22%3Atrue%7D&wdorigin=ItemsView&ctp=LeastProtected&rct=Normal&wdhostclicktime=1787156527792&afdflight=83&csiro=1&instantedit=1&wopicomplete=1&wdredirectionreason=Unified_SingleFlush#_ENREF_4)]. Higher burnout then increases the risk of sickness absence and turnover, further exacerbating staffing shortages [[5](https://ukc-word-edit.officeapps.live.com/we/wordeditorframe.aspx?ui=en-GB&rs=en-US&wopisrc=https%3A%2F%2Flivemanchesterac-my.sharepoint.com%2Fpersonal%2Fjudith_johnson_manchester_ac_uk%2F_vti_bin%2Fwopi.ashx%2Ffiles%2Fe7ceb6b0a8b24c46b9fca48e0c21f06a&wdenableroaming=1&mscc=1&wdodb=1&hid=15D032A2-003B-2001-C43C-45F56ADF876D.0&uih=sharepointcom&wdlcid=en-GB&jsapi=1&jsapiver=v2&corrid=fb1b269f-e890-2117-5f8a-b0b5636b4890&usid=fb1b269f-e890-2117-5f8a-b0b5636b4890&newsession=1&sftc=1&hfto=1787156527793.3&uihit=docaspx&muv=1&ats=PairwiseBroker&cac=1&sams=1&mtf=1&sfp=1&sdp=1&hch=1&hwfh=1&dchat=1&sc=%7B%22pmo%22%3A%22https%3A%2F%2Flivemanchesterac-my.sharepoint.com%22%2C%22pmshare%22%3Atrue%7D&wdorigin=ItemsView&ctp=LeastProtected&rct=Normal&wdhostclicktime=1787156527792&afdflight=83&csiro=1&instantedit=1&wopicomplete=1&wdredirectionreason=Unified_SingleFlush#_ENREF_5), [6](https://ukc-word-edit.officeapps.live.com/we/wordeditorframe.aspx?ui=en-GB&rs=en-US&wopisrc=https%3A%2F%2Flivemanchesterac-my.sharepoint.com%2Fpersonal%2Fjudith_johnson_manchester_ac_uk%2F_vti_bin%2Fwopi.ashx%2Ffiles%2Fe7ceb6b0a8b24c46b9fca48e0c21f06a&wdenableroaming=1&mscc=1&wdodb=1&hid=15D032A2-003B-2001-C43C-45F56ADF876D.0&uih=sharepointcom&wdlcid=en-GB&jsapi=1&jsapiver=v2&corrid=fb1b269f-e890-2117-5f8a-b0b5636b4890&usid=fb1b269f-e890-2117-5f8a-b0b5636b4890&newsession=1&sftc=1&hfto=1787156527793.3&uihit=docaspx&muv=1&ats=PairwiseBroker&cac=1&sams=1&mtf=1&sfp=1&sdp=1&hch=1&hwfh=1&dchat=1&sc=%7B%22pmo%22%3A%22https%3A%2F%2Flivemanchesterac-my.sharepoint.com%22%2C%22pmshare%22%3Atrue%7D&wdorigin=ItemsView&ctp=LeastProtected&rct=Normal&wdhostclicktime=1787156527792&afdflight=83&csiro=1&instantedit=1&wopicomplete=1&wdredirectionreason=Unified_SingleFlush#_ENREF_6)] and directly leading to the delivery of poorer patient care [[7](https://ukc-word-edit.officeapps.live.com/we/wordeditorframe.aspx?ui=en-GB&rs=en-US&wopisrc=https%3A%2F%2Flivemanchesterac-my.sharepoint.com%2Fpersonal%2Fjudith_johnson_manchester_ac_uk%2F_vti_bin%2Fwopi.ashx%2Ffiles%2Fe7ceb6b0a8b24c46b9fca48e0c21f06a&wdenableroaming=1&mscc=1&wdodb=1&hid=15D032A2-003B-2001-C43C-45F56ADF876D.0&uih=sharepointcom&wdlcid=en-GB&jsapi=1&jsapiver=v2&corrid=fb1b269f-e890-2117-5f8a-b0b5636b4890&usid=fb1b269f-e890-2117-5f8a-b0b5636b4890&newsession=1&sftc=1&hfto=1787156527793.3&uihit=docaspx&muv=1&ats=PairwiseBroker&cac=1&sams=1&mtf=1&sfp=1&sdp=1&hch=1&hwfh=1&dchat=1&sc=%7B%22pmo%22%3A%22https%3A%2F%2Flivemanchesterac-my.sharepoint.com%22%2C%22pmshare%22%3Atrue%7D&wdorigin=ItemsView&ctp=LeastProtected&rct=Normal&wdhostclicktime=1787156527792&afdflight=83&csiro=1&instantedit=1&wopicomplete=1&wdredirectionreason=Unified_SingleFlush#_ENREF_7)].

In a review of 170 studies in doctors, higher burnout was associated with twice the risk of patient safety incidents and a two-fold reduction in patient satisfaction [[7](https://ukc-word-edit.officeapps.live.com/we/wordeditorframe.aspx?ui=en-GB&rs=en-US&wopisrc=https%3A%2F%2Flivemanchesterac-my.sharepoint.com%2Fpersonal%2Fjudith_johnson_manchester_ac_uk%2F_vti_bin%2Fwopi.ashx%2Ffiles%2Fe7ceb6b0a8b24c46b9fca48e0c21f06a&wdenableroaming=1&mscc=1&wdodb=1&hid=15D032A2-003B-2001-C43C-45F56ADF876D.0&uih=sharepointcom&wdlcid=en-GB&jsapi=1&jsapiver=v2&corrid=fb1b269f-e890-2117-5f8a-b0b5636b4890&usid=fb1b269f-e890-2117-5f8a-b0b5636b4890&newsession=1&sftc=1&hfto=1787156527793.3&uihit=docaspx&muv=1&ats=PairwiseBroker&cac=1&sams=1&mtf=1&sfp=1&sdp=1&hch=1&hwfh=1&dchat=1&sc=%7B%22pmo%22%3A%22https%3A%2F%2Flivemanchesterac-my.sharepoint.com%22%2C%22pmshare%22%3Atrue%7D&wdorigin=ItemsView&ctp=LeastProtected&rct=Normal&wdhostclicktime=1787156527792&afdflight=83&csiro=1&instantedit=1&wopicomplete=1&wdredirectionreason=Unified_SingleFlush#_ENREF_7)]. Within this vicious cycle, provider burnout and poor wellbeing must be recognised as an active component, serving to drive the workforce crisis. Equally, addressing this component by better supporting healthcare professionals could help to break this cycle, leading to reduced sickness absence, better retention and better patient care [[8](https://ukc-word-edit.officeapps.live.com/we/wordeditorframe.aspx?ui=en-GB&rs=en-US&wopisrc=https%3A%2F%2Flivemanchesterac-my.sharepoint.com%2Fpersonal%2Fjudith_johnson_manchester_ac_uk%2F_vti_bin%2Fwopi.ashx%2Ffiles%2Fe7ceb6b0a8b24c46b9fca48e0c21f06a&wdenableroaming=1&mscc=1&wdodb=1&hid=15D032A2-003B-2001-C43C-45F56ADF876D.0&uih=sharepointcom&wdlcid=en-GB&jsapi=1&jsapiver=v2&corrid=fb1b269f-e890-2117-5f8a-b0b5636b4890&usid=fb1b269f-e890-2117-5f8a-b0b5636b4890&newsession=1&sftc=1&hfto=1787156527793.3&uihit=docaspx&muv=1&ats=PairwiseBroker&cac=1&sams=1&mtf=1&sfp=1&sdp=1&hch=1&hwfh=1&dchat=1&sc=%7B%22pmo%22%3A%22https%3A%2F%2Flivemanchesterac-my.sharepoint.com%22%2C%22pmshare%22%3Atrue%7D&wdorigin=ItemsView&ctp=LeastProtected&rct=Normal&wdhostclicktime=1787156527792&afdflight=83&csiro=1&instantedit=1&wopicomplete=1&wdredirectionreason=Unified_SingleFlush#_ENREF_8)].

Most burnout research has focused on qualified professional groups, but there is a growing recognition that it could be beneficial to intervene during training, prior to professional registration, due to high rates of mental health distress in these cohorts [[9](https://ukc-word-edit.officeapps.live.com/we/wordeditorframe.aspx?ui=en-GB&rs=en-US&wopisrc=https%3A%2F%2Flivemanchesterac-my.sharepoint.com%2Fpersonal%2Fjudith_johnson_manchester_ac_uk%2F_vti_bin%2Fwopi.ashx%2Ffiles%2Fe7ceb6b0a8b24c46b9fca48e0c21f06a&wdenableroaming=1&mscc=1&wdodb=1&hid=15D032A2-003B-2001-C43C-45F56ADF876D.0&uih=sharepointcom&wdlcid=en-GB&jsapi=1&jsapiver=v2&corrid=fb1b269f-e890-2117-5f8a-b0b5636b4890&usid=fb1b269f-e890-2117-5f8a-b0b5636b4890&newsession=1&sftc=1&hfto=1787156527793.3&uihit=docaspx&muv=1&ats=PairwiseBroker&cac=1&sams=1&mtf=1&sfp=1&sdp=1&hch=1&hwfh=1&dchat=1&sc=%7B%22pmo%22%3A%22https%3A%2F%2Flivemanchesterac-my.sharepoint.com%22%2C%22pmshare%22%3Atrue%7D&wdorigin=ItemsView&ctp=LeastProtected&rct=Normal&wdhostclicktime=1787156527792&afdflight=83&csiro=1&instantedit=1&wopicomplete=1&wdredirectionreason=Unified_SingleFlush#_ENREF_9)]. Studies in medical students indicate half report burnout, one in three report depression and one in ten experience suicidal thoughts [[10-12](https://ukc-word-edit.officeapps.live.com/we/wordeditorframe.aspx?ui=en-GB&rs=en-US&wopisrc=https%3A%2F%2Flivemanchesterac-my.sharepoint.com%2Fpersonal%2Fjudith_johnson_manchester_ac_uk%2F_vti_bin%2Fwopi.ashx%2Ffiles%2Fe7ceb6b0a8b24c46b9fca48e0c21f06a&wdenableroaming=1&mscc=1&wdodb=1&hid=15D032A2-003B-2001-C43C-45F56ADF876D.0&uih=sharepointcom&wdlcid=en-GB&jsapi=1&jsapiver=v2&corrid=fb1b269f-e890-2117-5f8a-b0b5636b4890&usid=fb1b269f-e890-2117-5f8a-b0b5636b4890&newsession=1&sftc=1&hfto=1787156527793.3&uihit=docaspx&muv=1&ats=PairwiseBroker&cac=1&sams=1&mtf=1&sfp=1&sdp=1&hch=1&hwfh=1&dchat=1&sc=%7B%22pmo%22%3A%22https%3A%2F%2Flivemanchesterac-my.sharepoint.com%22%2C%22pmshare%22%3Atrue%7D&wdorigin=ItemsView&ctp=LeastProtected&rct=Normal&wdhostclicktime=1787156527792&afdflight=83&csiro=1&instantedit=1&wopicomplete=1&wdredirectionreason=Unified_SingleFlush#_ENREF_10)]. Furthermore, the immediate post-qualification years have been recognised as a high-risk time for physicians leaving the profession [[13](https://ukc-word-edit.officeapps.live.com/we/wordeditorframe.aspx?ui=en-GB&rs=en-US&wopisrc=https%3A%2F%2Flivemanchesterac-my.sharepoint.com%2Fpersonal%2Fjudith_johnson_manchester_ac_uk%2F_vti_bin%2Fwopi.ashx%2Ffiles%2Fe7ceb6b0a8b24c46b9fca48e0c21f06a&wdenableroaming=1&mscc=1&wdodb=1&hid=15D032A2-003B-2001-C43C-45F56ADF876D.0&uih=sharepointcom&wdlcid=en-GB&jsapi=1&jsapiver=v2&corrid=fb1b269f-e890-2117-5f8a-b0b5636b4890&usid=fb1b269f-e890-2117-5f8a-b0b5636b4890&newsession=1&sftc=1&hfto=1787156527793.3&uihit=docaspx&muv=1&ats=PairwiseBroker&cac=1&sams=1&mtf=1&sfp=1&sdp=1&hch=1&hwfh=1&dchat=1&sc=%7B%22pmo%22%3A%22https%3A%2F%2Flivemanchesterac-my.sharepoint.com%22%2C%22pmshare%22%3Atrue%7D&wdorigin=ItemsView&ctp=LeastProtected&rct=Normal&wdhostclicktime=1787156527792&afdflight=83&csiro=1&instantedit=1&wopicomplete=1&wdredirectionreason=Unified_SingleFlush#_ENREF_13)]. Poor mental health and burnout are linked with higher attrition [[14](https://ukc-word-edit.officeapps.live.com/we/wordeditorframe.aspx?ui=en-GB&rs=en-US&wopisrc=https%3A%2F%2Flivemanchesterac-my.sharepoint.com%2Fpersonal%2Fjudith_johnson_manchester_ac_uk%2F_vti_bin%2Fwopi.ashx%2Ffiles%2Fe7ceb6b0a8b24c46b9fca48e0c21f06a&wdenableroaming=1&mscc=1&wdodb=1&hid=15D032A2-003B-2001-C43C-45F56ADF876D.0&uih=sharepointcom&wdlcid=en-GB&jsapi=1&jsapiver=v2&corrid=fb1b269f-e890-2117-5f8a-b0b5636b4890&usid=fb1b269f-e890-2117-5f8a-b0b5636b4890&newsession=1&sftc=1&hfto=1787156527793.3&uihit=docaspx&muv=1&ats=PairwiseBroker&cac=1&sams=1&mtf=1&sfp=1&sdp=1&hch=1&hwfh=1&dchat=1&sc=%7B%22pmo%22%3A%22https%3A%2F%2Flivemanchesterac-my.sharepoint.com%22%2C%22pmshare%22%3Atrue%7D&wdorigin=ItemsView&ctp=LeastProtected&rct=Normal&wdhostclicktime=1787156527792&afdflight=83&csiro=1&instantedit=1&wopicomplete=1&wdredirectionreason=Unified_SingleFlush#_ENREF_14)], so improving their mental wellbeing could be one way to enhance retention and reduce physician shortages.

Several causal factors for poor mental health and burnout have been identified which are specific to this group, including a high academic workload, a tendency towards maladaptive perfectionism [[15](https://ukc-word-edit.officeapps.live.com/we/wordeditorframe.aspx?ui=en-GB&rs=en-US&wopisrc=https%3A%2F%2Flivemanchesterac-my.sharepoint.com%2Fpersonal%2Fjudith_johnson_manchester_ac_uk%2F_vti_bin%2Fwopi.ashx%2Ffiles%2Fe7ceb6b0a8b24c46b9fca48e0c21f06a&wdenableroaming=1&mscc=1&wdodb=1&hid=15D032A2-003B-2001-C43C-45F56ADF876D.0&uih=sharepointcom&wdlcid=en-GB&jsapi=1&jsapiver=v2&corrid=fb1b269f-e890-2117-5f8a-b0b5636b4890&usid=fb1b269f-e890-2117-5f8a-b0b5636b4890&newsession=1&sftc=1&hfto=1787156527793.3&uihit=docaspx&muv=1&ats=PairwiseBroker&cac=1&sams=1&mtf=1&sfp=1&sdp=1&hch=1&hwfh=1&dchat=1&sc=%7B%22pmo%22%3A%22https%3A%2F%2Flivemanchesterac-my.sharepoint.com%22%2C%22pmshare%22%3Atrue%7D&wdorigin=ItemsView&ctp=LeastProtected&rct=Normal&wdhostclicktime=1787156527792&afdflight=83&csiro=1&instantedit=1&wopicomplete=1&wdredirectionreason=Unified_SingleFlush#_ENREF_15), [16](https://ukc-word-edit.officeapps.live.com/we/wordeditorframe.aspx?ui=en-GB&rs=en-US&wopisrc=https%3A%2F%2Flivemanchesterac-my.sharepoint.com%2Fpersonal%2Fjudith_johnson_manchester_ac_uk%2F_vti_bin%2Fwopi.ashx%2Ffiles%2Fe7ceb6b0a8b24c46b9fca48e0c21f06a&wdenableroaming=1&mscc=1&wdodb=1&hid=15D032A2-003B-2001-C43C-45F56ADF876D.0&uih=sharepointcom&wdlcid=en-GB&jsapi=1&jsapiver=v2&corrid=fb1b269f-e890-2117-5f8a-b0b5636b4890&usid=fb1b269f-e890-2117-5f8a-b0b5636b4890&newsession=1&sftc=1&hfto=1787156527793.3&uihit=docaspx&muv=1&ats=PairwiseBroker&cac=1&sams=1&mtf=1&sfp=1&sdp=1&hch=1&hwfh=1&dchat=1&sc=%7B%22pmo%22%3A%22https%3A%2F%2Flivemanchesterac-my.sharepoint.com%22%2C%22pmshare%22%3Atrue%7D&wdorigin=ItemsView&ctp=LeastProtected&rct=Normal&wdhostclicktime=1787156527792&afdflight=83&csiro=1&instantedit=1&wopicomplete=1&wdredirectionreason=Unified_SingleFlush#_ENREF_16)] and exposure to stressful clinical events on placement [[17](https://ukc-word-edit.officeapps.live.com/we/wordeditorframe.aspx?ui=en-GB&rs=en-US&wopisrc=https%3A%2F%2Flivemanchesterac-my.sharepoint.com%2Fpersonal%2Fjudith_johnson_manchester_ac_uk%2F_vti_bin%2Fwopi.ashx%2Ffiles%2Fe7ceb6b0a8b24c46b9fca48e0c21f06a&wdenableroaming=1&mscc=1&wdodb=1&hid=15D032A2-003B-2001-C43C-45F56ADF876D.0&uih=sharepointcom&wdlcid=en-GB&jsapi=1&jsapiver=v2&corrid=fb1b269f-e890-2117-5f8a-b0b5636b4890&usid=fb1b269f-e890-2117-5f8a-b0b5636b4890&newsession=1&sftc=1&hfto=1787156527793.3&uihit=docaspx&muv=1&ats=PairwiseBroker&cac=1&sams=1&mtf=1&sfp=1&sdp=1&hch=1&hwfh=1&dchat=1&sc=%7B%22pmo%22%3A%22https%3A%2F%2Flivemanchesterac-my.sharepoint.com%22%2C%22pmshare%22%3Atrue%7D&wdorigin=ItemsView&ctp=LeastProtected&rct=Normal&wdhostclicktime=1787156527792&afdflight=83&csiro=1&instantedit=1&wopicomplete=1&wdredirectionreason=Unified_SingleFlush#_ENREF_17)]. Indeed, rates of burnout in medical students increase significantly during the years involving clinical placements [[18](https://ukc-word-edit.officeapps.live.com/we/wordeditorframe.aspx?ui=en-GB&rs=en-US&wopisrc=https%3A%2F%2Flivemanchesterac-my.sharepoint.com%2Fpersonal%2Fjudith_johnson_manchester_ac_uk%2F_vti_bin%2Fwopi.ashx%2Ffiles%2Fe7ceb6b0a8b24c46b9fca48e0c21f06a&wdenableroaming=1&mscc=1&wdodb=1&hid=15D032A2-003B-2001-C43C-45F56ADF876D.0&uih=sharepointcom&wdlcid=en-GB&jsapi=1&jsapiver=v2&corrid=fb1b269f-e890-2117-5f8a-b0b5636b4890&usid=fb1b269f-e890-2117-5f8a-b0b5636b4890&newsession=1&sftc=1&hfto=1787156527793.3&uihit=docaspx&muv=1&ats=PairwiseBroker&cac=1&sams=1&mtf=1&sfp=1&sdp=1&hch=1&hwfh=1&dchat=1&sc=%7B%22pmo%22%3A%22https%3A%2F%2Flivemanchesterac-my.sharepoint.com%22%2C%22pmshare%22%3Atrue%7D&wdorigin=ItemsView&ctp=LeastProtected&rct=Normal&wdhostclicktime=1787156527792&afdflight=83&csiro=1&instantedit=1&wopicomplete=1&wdredirectionreason=Unified_SingleFlush#_ENREF_18)]. However, there has been a lack of interventions tailored for medical students and none which prepare medical students for stressful workplace events [[19](https://ukc-word-edit.officeapps.live.com/we/wordeditorframe.aspx?ui=en-GB&rs=en-US&wopisrc=https%3A%2F%2Flivemanchesterac-my.sharepoint.com%2Fpersonal%2Fjudith_johnson_manchester_ac_uk%2F_vti_bin%2Fwopi.ashx%2Ffiles%2Fe7ceb6b0a8b24c46b9fca48e0c21f06a&wdenableroaming=1&mscc=1&wdodb=1&hid=15D032A2-003B-2001-C43C-45F56ADF876D.0&uih=sharepointcom&wdlcid=en-GB&jsapi=1&jsapiver=v2&corrid=fb1b269f-e890-2117-5f8a-b0b5636b4890&usid=fb1b269f-e890-2117-5f8a-b0b5636b4890&newsession=1&sftc=1&hfto=1787156527793.3&uihit=docaspx&muv=1&ats=PairwiseBroker&cac=1&sams=1&mtf=1&sfp=1&sdp=1&hch=1&hwfh=1&dchat=1&sc=%7B%22pmo%22%3A%22https%3A%2F%2Flivemanchesterac-my.sharepoint.com%22%2C%22pmshare%22%3Atrue%7D&wdorigin=ItemsView&ctp=LeastProtected&rct=Normal&wdhostclicktime=1787156527792&afdflight=83&csiro=1&instantedit=1&wopicomplete=1&wdredirectionreason=Unified_SingleFlush#_ENREF_19)]. The mental health interventions which have been tested in this group have been largely generic and ‘off-the-shelf’, including mindfulness, relaxation training and yoga [[19](https://ukc-word-edit.officeapps.live.com/we/wordeditorframe.aspx?ui=en-GB&rs=en-US&wopisrc=https%3A%2F%2Flivemanchesterac-my.sharepoint.com%2Fpersonal%2Fjudith_johnson_manchester_ac_uk%2F_vti_bin%2Fwopi.ashx%2Ffiles%2Fe7ceb6b0a8b24c46b9fca48e0c21f06a&wdenableroaming=1&mscc=1&wdodb=1&hid=15D032A2-003B-2001-C43C-45F56ADF876D.0&uih=sharepointcom&wdlcid=en-GB&jsapi=1&jsapiver=v2&corrid=fb1b269f-e890-2117-5f8a-b0b5636b4890&usid=fb1b269f-e890-2117-5f8a-b0b5636b4890&newsession=1&sftc=1&hfto=1787156527793.3&uihit=docaspx&muv=1&ats=PairwiseBroker&cac=1&sams=1&mtf=1&sfp=1&sdp=1&hch=1&hwfh=1&dchat=1&sc=%7B%22pmo%22%3A%22https%3A%2F%2Flivemanchesterac-my.sharepoint.com%22%2C%22pmshare%22%3Atrue%7D&wdorigin=ItemsView&ctp=LeastProtected&rct=Normal&wdhostclicktime=1787156527792&afdflight=83&csiro=1&instantedit=1&wopicomplete=1&wdredirectionreason=Unified_SingleFlush#_ENREF_19)]. Evidence for these interventions is weak and mixed, and they have been criticised for lacking relevance [[20](https://ukc-word-edit.officeapps.live.com/we/wordeditorframe.aspx?ui=en-GB&rs=en-US&wopisrc=https%3A%2F%2Flivemanchesterac-my.sharepoint.com%2Fpersonal%2Fjudith_johnson_manchester_ac_uk%2F_vti_bin%2Fwopi.ashx%2Ffiles%2Fe7ceb6b0a8b24c46b9fca48e0c21f06a&wdenableroaming=1&mscc=1&wdodb=1&hid=15D032A2-003B-2001-C43C-45F56ADF876D.0&uih=sharepointcom&wdlcid=en-GB&jsapi=1&jsapiver=v2&corrid=fb1b269f-e890-2117-5f8a-b0b5636b4890&usid=fb1b269f-e890-2117-5f8a-b0b5636b4890&newsession=1&sftc=1&hfto=1787156527793.3&uihit=docaspx&muv=1&ats=PairwiseBroker&cac=1&sams=1&mtf=1&sfp=1&sdp=1&hch=1&hwfh=1&dchat=1&sc=%7B%22pmo%22%3A%22https%3A%2F%2Flivemanchesterac-my.sharepoint.com%22%2C%22pmshare%22%3Atrue%7D&wdorigin=ItemsView&ctp=LeastProtected&rct=Normal&wdhostclicktime=1787156527792&afdflight=83&csiro=1&instantedit=1&wopicomplete=1&wdredirectionreason=Unified_SingleFlush#_ENREF_20)]. There is an urgent need for effective, preventative interventions which help students cope with the specific occupational stressors they will encounter during their placements, training, and beyond.

The current trial will evaluate Thumos, an intervention that employs cognitive-behavioural therapy (CBT) techniques to prepare healthcare professionals and students to manage stressful work or training events. Thumos has been developed and refined over time in response to pilot testing and evaluations. Thumos differs from previously evaluated psychological interventions for medical students in three ways. Firstly, it has been designed with healthcare professionals in mind, based upon a combination of 1) research into the effects of patient safety incidents and other work stressors, 2) resilience theory, and 3) CBT techniques. Secondly, Thumos includes both one-to-one and group modalities, synergising the benefits of peer engagement through group workshops with professional, confidential support in a subsequent phone call. Thirdly, Thumos addresses the stressful situations that are intrinsic to healthcare work, providing support for those unavoidable events that cannot be improved through organisational interventions.

Characteristics of Thumos may make it particularly well suited for medical students. For example, it is a preventative rather than ameliorative intervention which could help to reduce stigma in participation; a known barrier for medical student uptake of psychological interventions [[11](https://ukc-word-edit.officeapps.live.com/we/wordeditorframe.aspx?ui=en-GB&rs=en-US&wopisrc=https%3A%2F%2Flivemanchesterac-my.sharepoint.com%2Fpersonal%2Fjudith_johnson_manchester_ac_uk%2F_vti_bin%2Fwopi.ashx%2Ffiles%2Fe7ceb6b0a8b24c46b9fca48e0c21f06a&wdenableroaming=1&mscc=1&wdodb=1&hid=15D032A2-003B-2001-C43C-45F56ADF876D.0&uih=sharepointcom&wdlcid=en-GB&jsapi=1&jsapiver=v2&corrid=fb1b269f-e890-2117-5f8a-b0b5636b4890&usid=fb1b269f-e890-2117-5f8a-b0b5636b4890&newsession=1&sftc=1&hfto=1787156527793.3&uihit=docaspx&muv=1&ats=PairwiseBroker&cac=1&sams=1&mtf=1&sfp=1&sdp=1&hch=1&hwfh=1&dchat=1&sc=%7B%22pmo%22%3A%22https%3A%2F%2Flivemanchesterac-my.sharepoint.com%22%2C%22pmshare%22%3Atrue%7D&wdorigin=ItemsView&ctp=LeastProtected&rct=Normal&wdhostclicktime=1787156527792&afdflight=83&csiro=1&instantedit=1&wopicomplete=1&wdredirectionreason=Unified_SingleFlush#_ENREF_11), 21, [22](https://ukc-word-edit.officeapps.live.com/we/wordeditorframe.aspx?ui=en-GB&rs=en-US&wopisrc=https%3A%2F%2Flivemanchesterac-my.sharepoint.com%2Fpersonal%2Fjudith_johnson_manchester_ac_uk%2F_vti_bin%2Fwopi.ashx%2Ffiles%2Fe7ceb6b0a8b24c46b9fca48e0c21f06a&wdenableroaming=1&mscc=1&wdodb=1&hid=15D032A2-003B-2001-C43C-45F56ADF876D.0&uih=sharepointcom&wdlcid=en-GB&jsapi=1&jsapiver=v2&corrid=fb1b269f-e890-2117-5f8a-b0b5636b4890&usid=fb1b269f-e890-2117-5f8a-b0b5636b4890&newsession=1&sftc=1&hfto=1787156527793.3&uihit=docaspx&muv=1&ats=PairwiseBroker&cac=1&sams=1&mtf=1&sfp=1&sdp=1&hch=1&hwfh=1&dchat=1&sc=%7B%22pmo%22%3A%22https%3A%2F%2Flivemanchesterac-my.sharepoint.com%22%2C%22pmshare%22%3Atrue%7D&wdorigin=ItemsView&ctp=LeastProtected&rct=Normal&wdhostclicktime=1787156527792&afdflight=83&csiro=1&instantedit=1&wopicomplete=1&wdredirectionreason=Unified_SingleFlush#_ENREF_22)]. It also uses case studies and materials which are tailored towards the stressful experiences medical students may face on placements. This approach increases the sense of relevance of the intervention for participants which may increase engagement [[9](https://ukc-word-edit.officeapps.live.com/we/wordeditorframe.aspx?ui=en-GB&rs=en-US&wopisrc=https%3A%2F%2Flivemanchesterac-my.sharepoint.com%2Fpersonal%2Fjudith_johnson_manchester_ac_uk%2F_vti_bin%2Fwopi.ashx%2Ffiles%2Fe7ceb6b0a8b24c46b9fca48e0c21f06a&wdenableroaming=1&mscc=1&wdodb=1&hid=15D032A2-003B-2001-C43C-45F56ADF876D.0&uih=sharepointcom&wdlcid=en-GB&jsapi=1&jsapiver=v2&corrid=fb1b269f-e890-2117-5f8a-b0b5636b4890&usid=fb1b269f-e890-2117-5f8a-b0b5636b4890&newsession=1&sftc=1&hfto=1787156527793.3&uihit=docaspx&muv=1&ats=PairwiseBroker&cac=1&sams=1&mtf=1&sfp=1&sdp=1&hch=1&hwfh=1&dchat=1&sc=%7B%22pmo%22%3A%22https%3A%2F%2Flivemanchesterac-my.sharepoint.com%22%2C%22pmshare%22%3Atrue%7D&wdorigin=ItemsView&ctp=LeastProtected&rct=Normal&wdhostclicktime=1787156527792&afdflight=83&csiro=1&instantedit=1&wopicomplete=1&wdredirectionreason=Unified_SingleFlush#_ENREF_9)]. Furthermore, it aims to reduce maladaptive perfectionism, a factor linked with mental health distress in medical students [[16](https://ukc-word-edit.officeapps.live.com/we/wordeditorframe.aspx?ui=en-GB&rs=en-US&wopisrc=https%3A%2F%2Flivemanchesterac-my.sharepoint.com%2Fpersonal%2Fjudith_johnson_manchester_ac_uk%2F_vti_bin%2Fwopi.ashx%2Ffiles%2Fe7ceb6b0a8b24c46b9fca48e0c21f06a&wdenableroaming=1&mscc=1&wdodb=1&hid=15D032A2-003B-2001-C43C-45F56ADF876D.0&uih=sharepointcom&wdlcid=en-GB&jsapi=1&jsapiver=v2&corrid=fb1b269f-e890-2117-5f8a-b0b5636b4890&usid=fb1b269f-e890-2117-5f8a-b0b5636b4890&newsession=1&sftc=1&hfto=1787156527793.3&uihit=docaspx&muv=1&ats=PairwiseBroker&cac=1&sams=1&mtf=1&sfp=1&sdp=1&hch=1&hwfh=1&dchat=1&sc=%7B%22pmo%22%3A%22https%3A%2F%2Flivemanchesterac-my.sharepoint.com%22%2C%22pmshare%22%3Atrue%7D&wdorigin=ItemsView&ctp=LeastProtected&rct=Normal&wdhostclicktime=1787156527792&afdflight=83&csiro=1&instantedit=1&wopicomplete=1&wdredirectionreason=Unified_SingleFlush#_ENREF_16)].

There have been three uncontrolled evaluations of previous versions of Thumos which indicate positive findings. The first was in a group of 66 multi-disciplinary healthcare professionals and students; results indicated it was associated with improvements in participants’ confidence, coping knowledge and psychological resilience [[23](https://ukc-word-edit.officeapps.live.com/we/wordeditorframe.aspx?ui=en-GB&rs=en-US&wopisrc=https%3A%2F%2Flivemanchesterac-my.sharepoint.com%2Fpersonal%2Fjudith_johnson_manchester_ac_uk%2F_vti_bin%2Fwopi.ashx%2Ffiles%2Fe7ceb6b0a8b24c46b9fca48e0c21f06a&wdenableroaming=1&mscc=1&wdodb=1&hid=15D032A2-003B-2001-C43C-45F56ADF876D.0&uih=sharepointcom&wdlcid=en-GB&jsapi=1&jsapiver=v2&corrid=fb1b269f-e890-2117-5f8a-b0b5636b4890&usid=fb1b269f-e890-2117-5f8a-b0b5636b4890&newsession=1&sftc=1&hfto=1787156527793.3&uihit=docaspx&muv=1&ats=PairwiseBroker&cac=1&sams=1&mtf=1&sfp=1&sdp=1&hch=1&hwfh=1&dchat=1&sc=%7B%22pmo%22%3A%22https%3A%2F%2Flivemanchesterac-my.sharepoint.com%22%2C%22pmshare%22%3Atrue%7D&wdorigin=ItemsView&ctp=LeastProtected&rct=Normal&wdhostclicktime=1787156527792&afdflight=83&csiro=1&instantedit=1&wopicomplete=1&wdredirectionreason=Unified_SingleFlush#_ENREF_23), [24](https://ukc-word-edit.officeapps.live.com/we/wordeditorframe.aspx?ui=en-GB&rs=en-US&wopisrc=https%3A%2F%2Flivemanchesterac-my.sharepoint.com%2Fpersonal%2Fjudith_johnson_manchester_ac_uk%2F_vti_bin%2Fwopi.ashx%2Ffiles%2Fe7ceb6b0a8b24c46b9fca48e0c21f06a&wdenableroaming=1&mscc=1&wdodb=1&hid=15D032A2-003B-2001-C43C-45F56ADF876D.0&uih=sharepointcom&wdlcid=en-GB&jsapi=1&jsapiver=v2&corrid=fb1b269f-e890-2117-5f8a-b0b5636b4890&usid=fb1b269f-e890-2117-5f8a-b0b5636b4890&newsession=1&sftc=1&hfto=1787156527793.3&uihit=docaspx&muv=1&ats=PairwiseBroker&cac=1&sams=1&mtf=1&sfp=1&sdp=1&hch=1&hwfh=1&dchat=1&sc=%7B%22pmo%22%3A%22https%3A%2F%2Flivemanchesterac-my.sharepoint.com%22%2C%22pmshare%22%3Atrue%7D&wdorigin=ItemsView&ctp=LeastProtected&rct=Normal&wdhostclicktime=1787156527792&afdflight=83&csiro=1&instantedit=1&wopicomplete=1&wdredirectionreason=Unified_SingleFlush#_ENREF_24)]. The second was in 80 Critical Care Nurses (CCNs) [[25](https://ukc-word-edit.officeapps.live.com/we/wordeditorframe.aspx?ui=en-GB&rs=en-US&wopisrc=https%3A%2F%2Flivemanchesterac-my.sharepoint.com%2Fpersonal%2Fjudith_johnson_manchester_ac_uk%2F_vti_bin%2Fwopi.ashx%2Ffiles%2Fe7ceb6b0a8b24c46b9fca48e0c21f06a&wdenableroaming=1&mscc=1&wdodb=1&hid=15D032A2-003B-2001-C43C-45F56ADF876D.0&uih=sharepointcom&wdlcid=en-GB&jsapi=1&jsapiver=v2&corrid=fb1b269f-e890-2117-5f8a-b0b5636b4890&usid=fb1b269f-e890-2117-5f8a-b0b5636b4890&newsession=1&sftc=1&hfto=1787156527793.3&uihit=docaspx&muv=1&ats=PairwiseBroker&cac=1&sams=1&mtf=1&sfp=1&sdp=1&hch=1&hwfh=1&dchat=1&sc=%7B%22pmo%22%3A%22https%3A%2F%2Flivemanchesterac-my.sharepoint.com%22%2C%22pmshare%22%3Atrue%7D&wdorigin=ItemsView&ctp=LeastProtected&rct=Normal&wdhostclicktime=1787156527792&afdflight=83&csiro=1&instantedit=1&wopicomplete=1&wdredirectionreason=Unified_SingleFlush#_ENREF_25), [26](https://ukc-word-edit.officeapps.live.com/we/wordeditorframe.aspx?ui=en-GB&rs=en-US&wopisrc=https%3A%2F%2Flivemanchesterac-my.sharepoint.com%2Fpersonal%2Fjudith_johnson_manchester_ac_uk%2F_vti_bin%2Fwopi.ashx%2Ffiles%2Fe7ceb6b0a8b24c46b9fca48e0c21f06a&wdenableroaming=1&mscc=1&wdodb=1&hid=15D032A2-003B-2001-C43C-45F56ADF876D.0&uih=sharepointcom&wdlcid=en-GB&jsapi=1&jsapiver=v2&corrid=fb1b269f-e890-2117-5f8a-b0b5636b4890&usid=fb1b269f-e890-2117-5f8a-b0b5636b4890&newsession=1&sftc=1&hfto=1787156527793.3&uihit=docaspx&muv=1&ats=PairwiseBroker&cac=1&sams=1&mtf=1&sfp=1&sdp=1&hch=1&hwfh=1&dchat=1&sc=%7B%22pmo%22%3A%22https%3A%2F%2Flivemanchesterac-my.sharepoint.com%22%2C%22pmshare%22%3Atrue%7D&wdorigin=ItemsView&ctp=LeastProtected&rct=Normal&wdhostclicktime=1787156527792&afdflight=83&csiro=1&instantedit=1&wopicomplete=1&wdredirectionreason=Unified_SingleFlush#_ENREF_26)]. Participation was associated with increased confidence and resilience and reduced depression and burnout. Nurses also reported a lower intention to leave clinical practice following the intervention [[25](https://ukc-word-edit.officeapps.live.com/we/wordeditorframe.aspx?ui=en-GB&rs=en-US&wopisrc=https%3A%2F%2Flivemanchesterac-my.sharepoint.com%2Fpersonal%2Fjudith_johnson_manchester_ac_uk%2F_vti_bin%2Fwopi.ashx%2Ffiles%2Fe7ceb6b0a8b24c46b9fca48e0c21f06a&wdenableroaming=1&mscc=1&wdodb=1&hid=15D032A2-003B-2001-C43C-45F56ADF876D.0&uih=sharepointcom&wdlcid=en-GB&jsapi=1&jsapiver=v2&corrid=fb1b269f-e890-2117-5f8a-b0b5636b4890&usid=fb1b269f-e890-2117-5f8a-b0b5636b4890&newsession=1&sftc=1&hfto=1787156527793.3&uihit=docaspx&muv=1&ats=PairwiseBroker&cac=1&sams=1&mtf=1&sfp=1&sdp=1&hch=1&hwfh=1&dchat=1&sc=%7B%22pmo%22%3A%22https%3A%2F%2Flivemanchesterac-my.sharepoint.com%22%2C%22pmshare%22%3Atrue%7D&wdorigin=ItemsView&ctp=LeastProtected&rct=Normal&wdhostclicktime=1787156527792&afdflight=83&csiro=1&instantedit=1&wopicomplete=1&wdredirectionreason=Unified_SingleFlush#_ENREF_25), [26](https://ukc-word-edit.officeapps.live.com/we/wordeditorframe.aspx?ui=en-GB&rs=en-US&wopisrc=https%3A%2F%2Flivemanchesterac-my.sharepoint.com%2Fpersonal%2Fjudith_johnson_manchester_ac_uk%2F_vti_bin%2Fwopi.ashx%2Ffiles%2Fe7ceb6b0a8b24c46b9fca48e0c21f06a&wdenableroaming=1&mscc=1&wdodb=1&hid=15D032A2-003B-2001-C43C-45F56ADF876D.0&uih=sharepointcom&wdlcid=en-GB&jsapi=1&jsapiver=v2&corrid=fb1b269f-e890-2117-5f8a-b0b5636b4890&usid=fb1b269f-e890-2117-5f8a-b0b5636b4890&newsession=1&sftc=1&hfto=1787156527793.3&uihit=docaspx&muv=1&ats=PairwiseBroker&cac=1&sams=1&mtf=1&sfp=1&sdp=1&hch=1&hwfh=1&dchat=1&sc=%7B%22pmo%22%3A%22https%3A%2F%2Flivemanchesterac-my.sharepoint.com%22%2C%22pmshare%22%3Atrue%7D&wdorigin=ItemsView&ctp=LeastProtected&rct=Normal&wdhostclicktime=1787156527792&afdflight=83&csiro=1&instantedit=1&wopicomplete=1&wdredirectionreason=Unified_SingleFlush#_ENREF_26)]. The third evaluation was in 115 medical students [[9](https://ukc-word-edit.officeapps.live.com/we/wordeditorframe.aspx?ui=en-GB&rs=en-US&wopisrc=https%3A%2F%2Flivemanchesterac-my.sharepoint.com%2Fpersonal%2Fjudith_johnson_manchester_ac_uk%2F_vti_bin%2Fwopi.ashx%2Ffiles%2Fe7ceb6b0a8b24c46b9fca48e0c21f06a&wdenableroaming=1&mscc=1&wdodb=1&hid=15D032A2-003B-2001-C43C-45F56ADF876D.0&uih=sharepointcom&wdlcid=en-GB&jsapi=1&jsapiver=v2&corrid=fb1b269f-e890-2117-5f8a-b0b5636b4890&usid=fb1b269f-e890-2117-5f8a-b0b5636b4890&newsession=1&sftc=1&hfto=1787156527793.3&uihit=docaspx&muv=1&ats=PairwiseBroker&cac=1&sams=1&mtf=1&sfp=1&sdp=1&hch=1&hwfh=1&dchat=1&sc=%7B%22pmo%22%3A%22https%3A%2F%2Flivemanchesterac-my.sharepoint.com%22%2C%22pmshare%22%3Atrue%7D&wdorigin=ItemsView&ctp=LeastProtected&rct=Normal&wdhostclicktime=1787156527792&afdflight=83&csiro=1&instantedit=1&wopicomplete=1&wdredirectionreason=Unified_SingleFlush#_ENREF_9)]. Findings indicated the intervention was feasible and potentially effective in this group, with participants reporting significant increases in resilience and confidence in coping with adverse events, and significant decreases in burnout and depression [[9](https://ukc-word-edit.officeapps.live.com/we/wordeditorframe.aspx?ui=en-GB&rs=en-US&wopisrc=https%3A%2F%2Flivemanchesterac-my.sharepoint.com%2Fpersonal%2Fjudith_johnson_manchester_ac_uk%2F_vti_bin%2Fwopi.ashx%2Ffiles%2Fe7ceb6b0a8b24c46b9fca48e0c21f06a&wdenableroaming=1&mscc=1&wdodb=1&hid=15D032A2-003B-2001-C43C-45F56ADF876D.0&uih=sharepointcom&wdlcid=en-GB&jsapi=1&jsapiver=v2&corrid=fb1b269f-e890-2117-5f8a-b0b5636b4890&usid=fb1b269f-e890-2117-5f8a-b0b5636b4890&newsession=1&sftc=1&hfto=1787156527793.3&uihit=docaspx&muv=1&ats=PairwiseBroker&cac=1&sams=1&mtf=1&sfp=1&sdp=1&hch=1&hwfh=1&dchat=1&sc=%7B%22pmo%22%3A%22https%3A%2F%2Flivemanchesterac-my.sharepoint.com%22%2C%22pmshare%22%3Atrue%7D&wdorigin=ItemsView&ctp=LeastProtected&rct=Normal&wdhostclicktime=1787156527792&afdflight=83&csiro=1&instantedit=1&wopicomplete=1&wdredirectionreason=Unified_SingleFlush#_ENREF_9)]. Qualitative findings from these evaluations indicate that participants appreciate the intervention’s tailored format and opportunity for peer support alongside professional input. They describe increased self-awareness and reported the acquisition of psychological skills which they could apply to cope with work stressors [[9](https://ukc-word-edit.officeapps.live.com/we/wordeditorframe.aspx?ui=en-GB&rs=en-US&wopisrc=https%3A%2F%2Flivemanchesterac-my.sharepoint.com%2Fpersonal%2Fjudith_johnson_manchester_ac_uk%2F_vti_bin%2Fwopi.ashx%2Ffiles%2Fe7ceb6b0a8b24c46b9fca48e0c21f06a&wdenableroaming=1&mscc=1&wdodb=1&hid=15D032A2-003B-2001-C43C-45F56ADF876D.0&uih=sharepointcom&wdlcid=en-GB&jsapi=1&jsapiver=v2&corrid=fb1b269f-e890-2117-5f8a-b0b5636b4890&usid=fb1b269f-e890-2117-5f8a-b0b5636b4890&newsession=1&sftc=1&hfto=1787156527793.3&uihit=docaspx&muv=1&ats=PairwiseBroker&cac=1&sams=1&mtf=1&sfp=1&sdp=1&hch=1&hwfh=1&dchat=1&sc=%7B%22pmo%22%3A%22https%3A%2F%2Flivemanchesterac-my.sharepoint.com%22%2C%22pmshare%22%3Atrue%7D&wdorigin=ItemsView&ctp=LeastProtected&rct=Normal&wdhostclicktime=1787156527792&afdflight=83&csiro=1&instantedit=1&wopicomplete=1&wdredirectionreason=Unified_SingleFlush#_ENREF_9), [23-26](https://ukc-word-edit.officeapps.live.com/we/wordeditorframe.aspx?ui=en-GB&rs=en-US&wopisrc=https%3A%2F%2Flivemanchesterac-my.sharepoint.com%2Fpersonal%2Fjudith_johnson_manchester_ac_uk%2F_vti_bin%2Fwopi.ashx%2Ffiles%2Fe7ceb6b0a8b24c46b9fca48e0c21f06a&wdenableroaming=1&mscc=1&wdodb=1&hid=15D032A2-003B-2001-C43C-45F56ADF876D.0&uih=sharepointcom&wdlcid=en-GB&jsapi=1&jsapiver=v2&corrid=fb1b269f-e890-2117-5f8a-b0b5636b4890&usid=fb1b269f-e890-2117-5f8a-b0b5636b4890&newsession=1&sftc=1&hfto=1787156527793.3&uihit=docaspx&muv=1&ats=PairwiseBroker&cac=1&sams=1&mtf=1&sfp=1&sdp=1&hch=1&hwfh=1&dchat=1&sc=%7B%22pmo%22%3A%22https%3A%2F%2Flivemanchesterac-my.sharepoint.com%22%2C%22pmshare%22%3Atrue%7D&wdorigin=ItemsView&ctp=LeastProtected&rct=Normal&wdhostclicktime=1787156527792&afdflight=83&csiro=1&instantedit=1&wopicomplete=1&wdredirectionreason=Unified_SingleFlush#_ENREF_23)].

Feedback from these evaluations was used to make improvements to further tailor the intervention towards medical students, including the addition of new exercises, reduction of didactic information and changes to the phone call. There is now a need to deliver the finalised version of the intervention, Thumos, and to ascertain whether observed improvements in outcome indicators can be attributed to the intervention. This study addresses this gap, through a definitive randomised controlled trial (RCT). We also extend the final follow-up time point in previous studies of 4 months to 9 months and create the possibility of conducting a subsequent 5-year follow-up study, should initial results be positive.

### ***Aims***

The main purpose of the trial is to establish whether Thumos increases resilience and improves mental health in medical students. The primary objective is to determine whether Thumos is superior to the control condition (services as usual; SAU) at 4 month follow-up in increasing psychological resilience in medical students. Secondary objectives are:

- To determine whether Thumos is superior to SAU at 9-month follow-up in increasing psychological resilience.
- To determine whether Thumos is superior to SAU at 4-month and 9-month follow-ups in the endpoints of confidence, burnout, depression, mental health and self-reported sickness absence.
- To assess acceptability and how Thumos is delivered in practice through a process evaluation with users, medical educators and providers.

## **Sec 3: Trial method**

**Table 1. Trial method summary**

| **Element** | **Specification** |
| --- | --- |
| **Design** | Two-arm, parallel group superiority RCT with individual randomisation 1:1 to Thumos or services as usual (SAU) |
| **Assessments** | T1 baseline; T2 post-intervention, or 6 weeks post-baseline for controls; T3 4 months (primary endpoint); T4 9 months. All questionnaires completed online via Qualtrics |
| **Eligibility** | UK medical students in a year containing a clinical placement of one week or longer, recruited nationally (typically Y4 or Y5) |
| **Sample size** | 220 randomised, 110 per arm |
| **Primary outcome** | Self-reported Brief Resilience Scale (BRS) score at T3 |
| **Secondary outcomes (quantitative)** | BRS at T4. At T3 and T4: Confidence in Coping with Adverse Events (CCAE), Oldenburg Burnout Inventory (OLBI), Patient Health Questionnaire 9 (PHQ-9), Recovering Quality of Life Utility Index (ReQoL-UI), self-reported sickness absence (novel measure). QALYs derived from ReQoL-UI utilities, baseline to T4 |
| **Primary analysis** | Mixed effects model for repeated measures with covariate adjustment, estimating the adjusted mean difference in BRS at T3 |
| **Interim analyses** | None planned |
| **Multiplicity** | Primary endpoint tested two-sided at α = .05. Secondary endpoints reported with 95% CIs and unadjusted p-values, with false discovery rate adjusted p-values alongside for transparency |
| **Statistical software** | R version 4.5.1, or the latest available at the time of analysis. All code version controlled |

## **Sec 4: Objectives & Hypotheses**

### ***Primary objective***

Determine whether Thumos increases resilience at 4 months versus SAU.

### ***Secondary objectives***

- Examine effects at T3 and T4 on confidence, burnout, depression, mental health utility and QALYs, and sickness absence
- Assess acceptability and delivery via mixed methods process evaluation (not outlined in this SAP)

### ***Main hypothesis***

We expect mean BRS at T3 to be higher in the Thumos group than in the SAU group. The trial was powered to detect a conventionally medium sized standardised mean difference (Cohen’s *d* = 0.5). Assuming a baseline SD of around 0.7, this is equivalent to a difference of about 0.35 BRS points.

## **Sec 5: Estimand(s)**

Estimands are specified following the ICH E9(R1) framework (Kahan et al. 2024) [1]. Analysis sets, the compliance definition, protocol deviations and assessment windows referred to below are defined in Section 8.

### ***Primary estimand***

**Table 2. Primary estimand**

| **Attribute** | **Specification** |
| --- | --- |
| **Estimand** | 'Policy' effect of offering Thumos on self-reported resilience at 4 months |
| **Treatment** | Assignment to Thumos versus assignment to services as usual |
| **Population** | UK medical students in a year containing a clinical placement of one week or longer, recruited nationally (typically Y4 or Y5) |
| **Variable** | Adjusted between-arm difference in mean BRS at T3 (approximately 4 months post-baseline), estimated by mixed models accounting for correlation between time points |
| **Summary measure** | Covariate adjusted unstandardised and standardised (*d*; spread = pooled baseline SD) mean difference in BRS at T3, Thumos minus control, with 95% CIs and *p*-values |
| **Intercurrent events and strategies** | Non-adherence to Thumos components. Treatment policy: analyse as allocated. |
|  | Use of external psychological support. Treatment policy: recorded at each time point and described |
|  | Missing T3 outcome. Not an intercurrent event as such but an estimation assumption: MAR given observed data, with planned sensitivity analyses relaxing MAR. |

### ***Secondary estimands***

Treatment, population and intercurrent event strategies are as for the primary estimand. Variables and summary measures differ by outcome. Secondary results are supportive rather than definitive (Section 10).

**Table 3. Secondary estimands**

| **Outcome** | **Time** | **Variable definition** | **Effect measure** |
| --- | --- | --- | --- |
| **BRS** | T4 | Mean of six items, range 1 to 5 | Adjusted mean difference and Cohen's *d*, 95% CI |
| **CCAE** | T3, T4 | Mean of three items | Adjusted mean difference and Cohen's *d*, 95% CI |
| **OLBI** | T3, T4 | Subscale means (exhaustion, disengagement) and total mean | Adjusted mean difference and Cohen's *d*, 95% CI |
| **PHQ-9** | T3, T4 | Sum of nine items, range 0 to 27 | Adjusted mean difference and Cohen's *d*, 95% CI |
| **ReQoL-UI** | T3, T4 | Utility index via the ReQoL-UI algorithm | Adjusted mean difference and Cohen's *d*, 95% CI |
| **ReQoL-UI QALYs** | Baseline to T4 | QALYs, area under the utility time curve with linear interpolation | Adjusted mean difference, 95% CI |
| **Sickness absence** | T3, T4 | Days lost in the past 4 weeks (two items, analysed separately) | Adjusted incidence rate ratio, 95% CI |
| BRS: Brief Resilience Scale; CCAE: Confidence in Coping with Adverse Events; OLBI: Oldenburg Burnout Inventory; PHQ-9: Patient Health Questionnaire 9 item depression scale; ReQoL-UI: Recovering Quality of Life Utility Index; QALYs: quality adjusted life years; T1 to T4: time points 1 to 4 (T = time). | | | |

### ***Supplementary estimand***

**Table 4. Supplementary estimand**

| **Attribute** | **Specification** |
| --- | --- |
| **Estimand** | Effect of receiving Thumos on resilience at 4 months among students who would take it up if offered |
| **Treatment** | Assignment to Thumos versus assignment to services as usual |
| **Population** | Students who would take up Thumos if it were offered to them. Take up means completing both group workshops, the minimum adherence definition in protocol section 9.5; the one-to-one call is not required.    For analysis this counts as attending both workshops within 6 weeks of randomisation with at least 75% of each session attended (Section 8). Take up is a characteristic of the student rather than of the arm they were allocated to, so it is seen directly in the Thumos arm and estimated statistically for the control arm, where nobody was offered the workshops. |
| **Variable** | Mean BRS at T3, defined as for the primary estimand, among students who would take up Thumos |
| **Summary measure** | Complier average causal effect: mean difference with 95% CI, standardised by the pooled baseline SD, reported alongside the primary effect |
| **Intercurrent events and strategies** | Non-adherence. Handled by narrowing who the estimate applies to (students who would take up Thumos), not by excluding or adjusting for anyone. All randomised participants remain in the analysis |
|  | External psychological support and missing T3 outcome. As for the primary estimand. |

## **Sec 6: Sample Size Estimation**

We aim to recruit and randomise 220 participants, with 110 in each arm. Simulations using the R package *powerlmm* showed that 200 participants would provide approximately 80% (or above) power to detect the target treatment effect at T3 under various combinations of assumptions (see Table 5). This target was further increased by 10% (i.e. to 220) as a conservative measure to allow for uncertainty in our simulations’ assumptions.

The target effect was informed by our earlier uncontrolled study; mean BRS increased from 2.72 at baseline to 3.17 (SD = 0.72) at four months, with a paired pre-post *d* = 0.89 (Johnson et al. (2023) [2]). Because this trial had no control group, and because a paired pre-post effect is not directly comparable with a between-group effect, we assumed a more conservative target: *d* = 0.50.

**Table 5. Power simulation tested assumptions**

| **Assumption** | **Value(s)** |
| --- | --- |
| **Primary comparison** | Adjusted difference in mean BRS between Thumos and services as usual at T3 |
| **Target effect** | Standardised between-group difference of *d* = 0.50. With a baseline SD of 0.70-0.90, *d* = 0.50 corresponds to approximately 0.35-0.45 BRS points, or 9-11% of the four point span of the 1-5 scale. As a rough convention, *d* values of 0.2, 0.5 and 0.8 are considered as small, medium and large (Cohen (1992) [3]) |
| **Power and significance level** | 80% power (beta = 20%) at a two-sided alpha of 0.05. |
| **Repeated measurements/within-participant correlation** | Up to three post-baseline BRS measurements per participant assumed. A range of participant ICC values were explored (0.30, 0.40, 0.50). The original BRS study reported test-retest correlations of 0.62-0.69, although these are not identical to model-based ICCs (Smith et al. (2008) [4]) |
| **Variation in change over time** | A range of variance-ratio values were examined (.01, .03, .05) (in *powerlmm*, this represents random-slope variance relative to within-participant residual variance) |
| **Attrition** | Monotone attrition by T4 was conservatively assumed to be 40% in the control and 30% in the intervention arm. These were deliberately cautious based mainly on pilot data (Johnson et al. (2023) [2]) and relevant published evidence (de Miquel et al. (2024) [5]) |

## **Sec 7: Randomisation, Concealment & Blinding**

### ***Randomisation & concealment***

Simple 1:1 individual randomisation via Sealed Envelope, a platform that generates and holds the allocation sequence. Allocation is revealed only after consent and baseline completion within Sealed Envelope; the sequence is not accessible to the trial team.

### ***Blinding***

Because the intervention is cognitive-behavioural and outcomes are self-reported, participants and the intervention team cannot be blinded.

The primary analysis will be conducted with the treatment groups labelled anonymously as A and B. The trial manager will record which arm is which, prepare the analysis dataset and remove or mask variables that could reveal allocation, such as intervention attendance and therapist details.

The allocation key will not be released until the primary analysis dataset has been locked and the masked primary results have been checked for completeness and accuracy.

Analyses requiring information about intervention receipt (e.g. per-protocol and CACE analyses) will be conducted after the allocation key is released. Any accidental unblinding will be documented and reported transparently.

## **Sec 8: Analysis Populations & Protocol Deviations**

### ***Analysis sets***

- **Full analysis set (FAS aka. ITT: intention to treat, primary analysis)**

All randomised participants, analysed as allocated, regardless of intervention receipt or post-randomisation events. Participants with no post-baseline outcomes appear in flow and baseline tables but will not contribute to outcome models. This set underpins the treatment policy estimands (as outlined above).

- **Per protocol set (PP, sensitivity analysis)**

FAS excluding major protocol deviations and excluding Thumos participants who do not meet the compliance definition (see below). Requires a valid T3 BRS within the T3 window.

- **CACE (supplementary estimand)**

Binary indicator of ‘full receipt’ (aka take up) used in the instrumental variables (IV) analysis (see Section 13). All randomised participants will remain in the IV analysis.

### ***Compliance definition (relevant to PP/CACE only)***

For descriptive reporting only, we may present a stricter completion metric that includes the one-to-one call, but PP and CACE will use the minimum adherence definition below to avoid ambiguity:

- **Thumos arm**

Attended both group workshops within 6 weeks of randomisation, with at least 75% attendance of each session. The one-to-one call is not required for minimum adherence.

- **Control arm**

No receipt of any Thumos component. Accessing usual external psychological support is allowed in both arms and is not a deviation (as this is not specified within the trial protocol).

### ***Protocol deviations***

All deviations will be logged and classified.

- **Minor**

Unlikely to affect the primary outcome or its interpretation, for example small lateness to sessions, questionnaire completed within the window but not on the target day. Minor deviations remain in all analysis sets.

- **Major**

May affect the primary outcome. These include:

- Invalid consent or ineligibility
- Allocation error/breach of concealment
- Any receipt of Thumos intervention in the SAU/control arm
- No valid T3 BRS within the prespecified T3 window
- Data integrity issues such as very unusual or impossible values recorded, or identified falsification

Major deviations remain in the FAS but will be excluded from PP. Non-completion of Thumos is not a deviation for the FAS, but it excludes participants from the PP sample and defines non-receipt for the CACE indicator.

### ***Timing rules***

Assessment windows are: T2 = 4–10 weeks, T3 = 12–24 weeks, and T4 = 30–44 weeks post-baseline. Where multiple responses fall within a window, the response closest to the nominal time point will be used.

Any assessments outside these windows will be documented, with the number and timing reported in the main paper. If the proportion of out-of-window assessments is substantial, a sensitivity analysis will assess the impact on results.

## **Sec 9: Outcomes**

### ***Overview***

All measures are administered online via Qualtrics at T1 (baseline), T2, T3 and T4. T3 and T4 are the pre-specified endpoints for efficacy analyses; T1 provides the baseline covariate and T2 contributes to the repeated measures models in Sections 12 and 13.

Table 6 sets out how each outcome is scored, including what happens when individual items are missing from within a measure. The rules and thresholds in the final column are justified below.

### ***Scoring and item level missingness***

Most key items are set as required in Qualtrics, so missing items within an otherwise completed measure should be rare and mainly where a participant exits partway through. Measures completed up to that point are scored normally; the interrupted measure follows the rules in Table 6.

Where published guidance on within scale missingness exists we follow it (NHS Digital for the PHQ-9; ReQoL-UI scoring documentation; Keetharuth et al. (2018) [6]). Otherwise we use person mean substitution, standard practice for patient reported outcome scales, allowing at most one missing item per scale or subscale (effective thresholds of 10-17%, supported by simulation work; Huisman (2000) [7]; Parent (2013) [8]; Jacoby et al. (2022) [9]). Beyond the threshold, scores are set to missing at that time point and handled at the analysis stage by the mixed models and multiple imputation (Section 11).

### ***Scale reliability***

Multi-item scales are useful only if their items measure the intended construct reliability in the population being studied. Several measures here have limited UK validation data in medical students. Poor reliability reduces treatment effects and complicates interpretation, so internal consistency will be reported at each time point to document how the scales perform in this sample and to support the validity of our efficacy conclusions. The estimates and thresholds are specified in Section 10.

**Table 6. Outcome scoring and handling of missing items**

| **Outcome** | **Items** | **Construct/direction** | **Endpoints** | **Derived variable** | **If items are missing** |
| --- | --- | --- | --- | --- | --- |
| **BRS (primary)** | 6 | Resilience; higher is better | T3 [12 to 24 wks] | Mean of six items after required reverse scoring (range 1 to 5) | Score if at least 5 items present, imputing the single missing item by person mean; otherwise missing (max = 16.7%) |
| **BRS** | 6 | Resilience; higher is better | T4 [30 to 44 wks] | As above | As above |
| **CCAE** | 3 | Confidence in coping with adverse events; higher is better | T3, T4 | Mean of three items | All three items required; no imputation (max 0%) |
| **OLBI (total and subscales)** | 16, as two 8 item subscales | Burnout: exhaustion and disengagement; higher is worse | T3, T4 | Subscale means and total mean, after required reverse scoring | Subscale scored if at least 7 of 8 items present, imputing the single missing item by subscale mean; if either subscale cannot be scored, the total is missing (max = 12.5%) |
| **PHQ-9** | 9 | Depressive symptoms; higher is worse | T3, T4 | Sum of nine items (range 0 to 27) | Score if at least 8 items present, imputing the single missing item by person mean and rounding the total to the nearest integer; otherwise missing (max = 11.1%) |
| **ReQoL-UI (utility)** | 10 | Quality of life utility; higher is better | T3, T4 | Utility index via the ReQoL-UI algorithm | Score if at least 9 items present, imputing by person mean; otherwise missing (max = 10%) |
| **ReQoL-UI QALYs** | 10 | Quality adjusted life years; higher is better | Baseline to T4 | Area under the utility time curve (Section 13) | Derived from the utility scores above; a missing utility at any time point is handled at the analysis stage (Sections 11 and 13) |
| **Sickness absence** | 2 | Days lost in past 4 weeks; higher is worse | T3, T4 | Two counts, analysed separately: total days lost, and working or studying days lost | Standalone items; no item level imputation |

BRS: Brief Resilience Scale; CCAE: Confidence in Coping with Adverse Events questionnaire; OLBI: Oldenburg Burnout Inventory; PHQ-9: Patient Health Questionnaire 9 item depression scale; ReQoL-UI: Recovering Quality of Life Utility Index; QALYs: quality adjusted life years. Person mean substitution means replacing a missing item with the mean of that participant's completed items on the same scale or subscale at the same time point. Support access is collected at all four time points but is only summarised descriptively by arm and time point as context for the treatment policy strategy in Section 5, and is not used as a covariate in any model.

## **Sec 10: Statistical Reporting**

See Section 15 for example R code snippets.

### ***Primary inference & analysis scale***

For the primary analysis, the BRS at T3 will be analysed with a two-sided significance level of α = 0.05. Although our hypothesis is that Thumos is beneficial, effects could plausibly occur in either direction. All continuous outcomes will be analysed on their original scales to retain clinical interpretability (e.g. points on the BRS).

### ***Effect measures and standardisation***

For continuous outcomes, Cohen's *d* is reported alongside the adjusted mean difference to give a scale-free measure comparable across outcomes and to link back to the target effect size calculation (Section 6). The standardiser is the pooled baseline (T1) SD of the relevant outcome. The ratio of the observed T3 SD to the baseline SD will be reported once, allowing readers to convert if they want as the choice can meaningfully alter *d* (e.g. a 0.35 point BRS difference is *d* = 0.50 against a baseline SD of 0.70 but *d* = 0.58 where SD = 0.60).

Count outcomes (the two sickness absence items) are reported as adjusted incidence rate ratios. These are already scale-free, so no standardised effect size is calculated for them. A rate ratio on its own can mislead when the underlying rate is low, so each is reported with the adjusted marginal mean days lost in both arms. The absolute difference in days lost, with its 95% CI, will be given alongside the ratio.

QALYs are reported as an adjusted mean difference only (Section 13).

Adverse and serious adverse events are reported as risk differences with 95% CIs, not as ratios (Section 13).

### ***Model diagnostics/robustness***

Model assumptions will be examined using residual and influence diagnostics. If there is clear evidence of severe non-normality or heteroskedasticity, we will repeat the analysis with *clubSandwich* (R package) robust standard errors as a sensitivity (clustered by participant for repeated measures). In all cases, the target estimand remains the adjusted mean difference on the original scale.

### ***Reporting, baseline imbalance & multiplicity***

Every endpoint will be reported as a point estimate with a 95% confidence interval and an exact *p*-value to three decimal places (for example *p* = .054), with values below .001 reported as *p* < .001. The number and percentage of participants contributing to each model will be given by arm and time point alongside every result.

Baseline characteristics will be described, not tested. Because allocation was random, any differences between the arms at baseline arose by chance, and testing whether chance occurred is not a useful question. We will instead report standardised differences, a measure of how far apart the arms are relative to the spread of the variable, and flag anything above 0.20 (just for the sake of interpretation).

We will not apply a formal correction for multiple testing when drawing our main conclusions. The primary endpoint is a single test at α = 0.05. Secondary and exploratory findings will be treated as supportive rather than definitive whatever their *p*-values show. For transparency about the risk of chance positives, we will additionally report false discovery rate adjusted *p*-values for the secondary outcomes, calculated by the Benjamini-Hochberg procedure within two families: all secondary outcomes at T3 and all at T4.

### ***Scale reliability***

Following the rationale set out in Section 9, we will report internal consistency for each multi-item scale at every time point. Cronbach's α and McDonald's ω will be estimated with 95% bootstrap CIs (1000 resamples), pooled across arms per time point and by arm at baseline. Thresholds of 0.70 will be considered acceptable, 0.80 good and 0.90 excellent. For the three-item CCAE we will also report the mean inter-item correlation, with 0.20 to 0.50 indicating adequate coherence, since α is unreliable on three items and will not be interpreted alone.

## **Sec 11: Missing data**

See Section 15 for example R code snippets.

Primary analyses use all available data. Each participant can contribute up to three post-baseline BRS observations (T2-T4). Scoring follows Section 9; where a scale cannot be scored, it is set to missing for that outcome and time point.

The primary analysis assumes outcome data are missing at random (MAR) given observed outcomes and model covariates. We will describe the pattern and predictors of missingness to judge how plausible that is: response rates by arm and time; stated reasons for non-completion where available; and comparisons of baseline characteristics and earlier outcomes (for example BRS at T1 and T2) between participants who do and do not provide BRS at T3 and T4, including logistic regression models with missingness as the outcome.

We will also conduct three types of sensitivity analysis for missing outcome data (Table 7).

**Table 7. Sensitivity analyses for missing outcome data**

| **Analysis** | **Specification** |
| --- | --- |
| **Multiple imputation under MAR** | Imputation models include outcomes at all time points, randomised group, and key baseline covariates (baseline BRS, age, gender, medical school, and baseline values of secondary outcomes where relevant). Carried out separately by arm, reflecting each outcome's type. The analysis mirrors the primary model and pools estimates by Rubin's rules |
| **Pattern mixture delta adjustment** | Fixed negative offsets of 0.10, 0.20 and 0.30 BRS points applied to imputed values for (intervention) participants missing at T3 or T4. Given an expected BRS SD of 0.7 to 0.9, these correspond to roughly 0.15 to 0.45 SDs, keeping the range within clinically plausible bounds |
| **Last post-baseline carried forward** | The most recent observed post-baseline value replaces a missing endpoint: T3 carried to T4, T2 carried to T3 or T4. Where no post-baseline value exists, baseline is carried forward |

Results will be considered robust if the direction and magnitude of the primary T3 BRS effect are broadly consistent across the primary analysis and these sensitivity analyses. We will then consider which assumptions are most plausible given the observed missingness patterns and predictors of dropout, and discuss the range of plausible treatment effects rather than relying on a single estimate.

## **Sec 12: Primary Analysis**

See Section 15 for example R code snippets.

- **Model & outcome**

The primary outcome is BRS at T3, scored as the mean of the six BRS items (range 1–5) at the T3 assessment window (12–24 weeks). Each participant can contribute up to three post-baseline BRS measurements at T2, T3 and T4.

The primary analysis will use a linear mixed model for repeated measures (MMRM) with BRS at T2, T3 and T4 as the repeated outcome and baseline BRS (T1) included as a covariate. The primary contrast is the adjusted between-group difference in mean BRS at T3 (Thumos minus services as usual) estimated from this model.

T4 occurs after T3, but including BRS at T2 and T4 in the model does not change the definition of the primary outcome. The MMRM jointly models the mean trajectory over time and the within-participant correlation structure. It allows all available post-baseline BRS data to contribute to estimation and gives an efficient and unbiased estimate of the T3 treatment contrast under the missing-at-random assumptions described in Section 11.

- **Specification**

Fixed effects will include:

- treatment group (Thumos vs services as usual)
- categorical time (T2, T3, T4)
- treatment-by-time interaction
- baseline BRS (T1)
- baseline covariates: age, gender, and medical school

Baseline BRS is expected to be strongly predictive of BRS at T3 and later, so adjusting for it will increase the precision (power) of the treatment effect estimate. Age, gender and medical school are pre-randomisation characteristics that may also be associated with BRS and with missingness. Including them also improves precision and supports a more plausible missing-at-random mechanism by conditioning on key baseline predictors. Randomisation already protects against confounding, so not adjusting for these covariates would not bias the treatment effect, but it would generally mean less precise estimates.

The model will include a random intercept for participant to account for clustering of repeated BRS scores within students. The within-subject covariance for the residuals at T2–T4 will be specified as unstructured in the primary analysis, allowing each time-specific variance and each covariance to be estimated freely. If this model fails to converge, we will use a more parsimonious structure, such as first-order autoregressive. Degrees of freedom for tests and confidence intervals for fixed effects will be estimated using Kenward-Roger approximation.

- **Diagnostics**

Model assumptions will be checked using:

- residual plots (fitted values vs residuals) and Q-Q plots for normality
- assessment of influential observations and fit indices (for example AIC/BIC)
- inspection of estimated covariance parameters (including whether simpler structures such as AR(1) provide an adequate fit)

If serious departures from assumptions are detected, we will report their impact through pre-specified sensitivity analyses (for example, models with robust standard errors or alternative covariance structures).

## **Sec 13: Additional Analyses**

See Section 15 for example R code snippets.

### ***Overview***

Unless Table 8 states otherwise, secondary outcomes use the same repeated measures framework as the primary analysis: fixed effects for treatment, categorical time, treatment by time, the baseline value of that outcome, and age, gender and medical school; a participant random intercept; unstructured within-participant covariance with AR(1) as a fallback if that fails to converge.

The focus for each outcome is the adjusted between-arm contrast at T3, with T4 reported alongside. Every outcome is reported as adjusted marginal means by arm, a contrast with 95% CI and exact *p*-value, and an FDR adjusted *p*-value within its time point family (Section 10).

Thumos is delivered in therapist led groups of roughly 6 to 8, but randomisation is at the individual level and the control arm has no equivalent grouping, so group and therapist effects are not modelled.

### ***Per protocol and CACE***

The per protocol and CACE analyses need information on who received the intervention, which cannot be used under the masked A and B labelling. They follow lock of the primary analysis dataset, checking of the masked primary results, and release of the allocation key (Section 7).

- **Per protocol (PP)**

We will repeat the primary analysis in the per protocol set, defined in line with Section 8.

- **Complier average causal effect (CACE)**

We will estimate the CACE for BRS at T3 by two stage least squares, using randomised allocation as an instrument for a binary take up indicator (Section 5; Angrist et al. (1996) [10]; Dunn et al. (2005) [11]; Boatman et al. (2018) [12]). Baseline BRS, age, gender and medical school will be included in both stages. Estimation will use a single instrumental variables fit rather than two regressions run in sequence, since the manual two-step version produces the same point estimate but standard errors that are too small.

We will report the effect among students who would take up Thumos with its 95% CI, the estimated proportion of the Thumos arm meeting the take up definition, and the first stage partial *F* statistic as a measure of instrument strength (<10 = weak).

**Table 8. Specification of additional analyses**

| **Analysis** | **Model** | **Effect measure** | **Notes** |
| --- | --- | --- | --- |
| **Continuous secondary outcomes:**  BRS (T4), CCAE, OLBI total and subscales, PHQ-9, ReQoL-UI utility | Linear MMRM as above | Adjusted mean difference and Cohen's *d*, 95% CI | Standardiser for *d* as in Section 10. OLBI subscales interpreted alongside the total as one finding |
| **Sickness absence:**  total days lost, and working or studying days lost, in past 4 weeks (analysed separately) | Poisson GLMM, log link | Adjusted incidence rate ratio, 95% CI, with adjusted mean days lost per arm | Negative binomial if overdispersed |
| **QALYs:**  baseline to T4 | Linear regression, one value per participant, adjusted for baseline utility, age, gender and medical school | Adjusted mean difference, 95% CI | Area under the utility time curve with linear interpolation (Manca et al. (2005) [13]). Run within the multiple imputation framework (Section 11) |
| **Support access (four items)** | None | None | Descriptive only, by arm and time point. Context for the treatment policy strategy in Section 5; not used as a covariate |
| **Per protocol** | Primary model refitted in the per protocol set (Section 8) | Adjusted mean difference in BRS at T3, 95% CI | Reported with the number and baseline characteristics of those excluded |
| **Complier average causal effect** | Two stage least squares, allocation instrumenting take up | Mean difference among students who would take up Thumos, 95% CI, raw and standardised | Specification above |
| **Safety** | None | Risk difference, 95% CI | Participants with at least one AE and at least one SAE by arm, with narrative summaries |

## **Sec 14: SAP Reporting Checklist**

In accordance with Gamble et al. (2017) [14]

**Table 9. SAP reporting checklist**

| **Section/Item** | **Index** | **Checklist description as written** | **How addressed** |
| --- | --- | --- | --- |
| **Section 1: administrative information** |  |  |  |
| Trial and trial registration | 1a | Descriptive title that matches the protocol, with SAP either as a forerunner or subtitle, and trial acronym (if applicable) | A randomised controlled evaluation of Thumos: a cognitive-behavioural intervention for medical students. Acronym: ACTIVATE |
|  | 1b | Trial registration number | ISRCTN 12744098; REC reference 2024-21589-38791 |
| SAP version | 2 | SAP version number with dates | Version 1, dated 13/08/2026 (seventh iteration; Section 16) |
| Protocol version | 3 | Reference to version of protocol being used | Protocol version 2.3, dated 15/01/2026 |
| SAP revisions | 4a | SAP revision history | Section 16 |
|  | 4b | Justification for each SAP revision | Section 16 |
|  | 4c | Timing of SAP revisions in relation to interim analyses, etc. | N/A, no interim analyses planned |
| Roles and responsibility | 5 | Names, affiliations, and roles of SAP contributors | SAP author and trial statistician: Dr Luke Budworth. TSC independent statistician: Prof Mona Kanaan. Chief Investigator: Dr Judith Johnson. Trial Co-Investigator: Dr Chris Taylor. Sponsor: University of Manchester. Funder: MPS Foundation |
| Signatures | 6a | Person writing the SAP | Dr Luke Budworth (Section 1) |
|  | 6b | Senior statistician responsible | Prof Mona Kanaan (Section 1) |
|  | 6c | Chief investigator/clinical lead | Dr Judith Johnson (Section 1) |
| **Section 2: introduction** |  |  |  |
| Background and rationale | 7 | Synopsis of trial background and rationale including a brief description of research question and brief justification for undertaking the trial | Section 2. Evaluates whether Thumos improves resilience and related outcomes in UK medical students in clinical placement years; full rationale in the protocol |
| Objectives | 8 | Description of specific objectives or hypotheses | Section 4. Primary: effect on BRS at T3 (4 months). Secondary: confidence, burnout, depression, mental health utility, QALYs and sickness absence at T3 and T4. Hypothesis: adjusted mean difference at T3 corresponding to d ≈ 0.50 |
| **Section 3: study methods** |  |  |  |
| Trial design | 9 | Brief description of trial design including type of trial (e.g., parallel group, multi-arm, crossover, factorial) and allocation ratio and may include brief description of interventions | Table 1. Two-arm parallel group superiority RCT, 1:1 individual randomisation to Thumos versus services as usual. T1 baseline, T2 around 6 weeks, T3 4 months (primary), T4 9 months. All questionnaires online via Qualtrics |
| Randomisation | 10 | Randomisation details, e.g., whether any minimisation or stratification occurred (including stratifying factors used or the location of that information if it is not held within the SAP) | Section 7. Simple 1:1 via Sealed Envelope, which generates and holds the sequence. No stratification or minimisation. Allocation revealed only after consent and baseline completion |
| Sample size | 11 | Full sample size calculation or reference to sample size calculation in protocol (instead of replication in SAP) | Section 6 and Table 5. Target 220 randomised (110 per arm), simulated in powerlmm. Protocol v2.3 permits recruiting above target to replace post-randomisation withdrawals, capped at 10% overall |
| Framework | 12 | Superiority, equivalence, or noninferiority hypothesis testing framework, including which comparisons will be presented on this basis | Superiority, for all comparisons |
| Statistical interim analysis and stopping guidance | 13a | Information on interim analyses specifying what interim analyses will be carried out and listing of time points | None planned |
|  | 13b | Any planned adjustment of the significance level due to interim analysis | N/A |
|  | 13c | Details of guidelines for stopping the trial early | Specified in the protocol (sections 10 and 13.3), not the SAP |
| Timing of final analysis | 14 | Timing of final analysis, e.g., all outcomes analysed collectively or timing stratified by planned length of follow-up | Section 7. All outcomes analysed collectively after follow-up completion and data lock. Allocation key released only after the dataset is locked and the masked primary results checked |
| **Section 4: statistical principles** |  |  |  |
| Confidence intervals and p values | 15 | Level of statistical significance | Section 10. Primary inference two-sided at α = .05 for BRS at T3, with emphasis on estimation rather than dichotomous significance |
|  | 16 | Description and rationale for any adjustment for multiplicity and, if so, detailing how the type 1 error is to be controlled | Section 10. No formal multiplicity adjustment; the primary endpoint is a single test and carries the trial's conclusion. False discovery rate adjusted p-values (Benjamini-Hochberg) reported for secondary endpoints within two families, T3 and T4, for transparency only |
|  | 17 | Confidence intervals to be reported | 95% CIs for all estimates (Section 10) |
| Adherence and protocol deviations | 18a | Definition of adherence to the intervention and how this is assessed including extent of exposure | Section 8. Minimum adherence, per protocol section 9.5, is completion of both group workshops. Operationalised as attendance at both workshops within 6 weeks of randomisation with at least 75% of each session attended. The one-to-one call is not required. Control arm: no receipt of any Thumos component |
|  | 18b | Description of how adherence to the intervention will be presented | Section 13. Adherence summarised descriptively by arm. PP analysis excludes non-adherent Thumos participants and major deviations; CACE estimated by two stage least squares with allocation instrumenting take up |
|  | 18c | Definition of protocol deviations for the trial | Section 8. Classified minor or major. Major: invalid consent or ineligibility, allocation error or concealment breach, any Thumos receipt in the control arm, no valid T3 BRS within window, data integrity issues |
|  | 18d | Description of which protocol deviations will be summarized | Section 8. All deviations logged and summarised by type and arm before the allocation key is released |
| **Section 5: trial population** |  |  |  |
| Analysis populations | 19 | Definition of analysis populations, e.g., intention to treat, per protocol, complete case, safety | Section 8. Full analysis set (ITT): all randomised, analysed as allocated. Per protocol: FAS excluding major deviations and non-adherent Thumos participants, requiring a valid T3 BRS in window. CACE: all randomised, with a binary take up indicator instrumented by allocation |
| Screening data | 20 | Reporting of screening data (if collected) to describe representativeness of trial sample | Documented in the protocol, not the SAP |
| Eligibility | 21 | Summary of eligibility criteria | Table 1. UK medical students in a year containing a clinical placement of one week or longer, recruited nationally (typically Y4 or Y5). Full criteria in protocol sections 5.1 and 5.2 |
| Recruitment | 22 | Information to be included in the CONSORT flow diagram | Screened where applicable, randomised, received intervention, followed up at T2 to T4, analysed, with reasons for non-participation and loss. Shell in the protocol |
| Withdrawal or follow-up | 23a | Level of withdrawal, e.g., from intervention and/or from follow-up | Withdrawal from intervention and from follow-up summarised separately, by arm and time point |
|  | 23b | Timing of withdrawal/lost to follow-up data | Reported against the T2, T3 and T4 windows (Section 8) |
|  | 23c | Reasons and details of how withdrawal/lost to follow-up data will be presented | Listed and summarised descriptively in the CONSORT diagram and the missing data summary (Section 11) |
| Baseline patient characteristics | 24a | List of baseline characteristics to be summarized | Age, gender, year of study, medical school, and baseline outcomes (BRS, CCAE, OLBI, PHQ-9, ReQoL-UI) |
|  | 24b | Details of how baseline characteristics will be descriptively summarized | Section 10. Continuous: mean and SD, or median and IQR if skewed. Categorical: n and %. Absolute standardised differences between arms reported and flagged above 0.20. Baseline balance is described, not tested |
| **Section 6: analysis** |  |  |  |
| Outcome definitions | 25a | Specification of outcomes and timings. If applicable include the order of importance of primary or key secondary end points (e.g., order in which they will be tested) | Tables 2, 3 and 6. Primary: BRS at T3. Secondary: BRS at T4; CCAE, OLBI (total and subscales), PHQ-9 and ReQoL-UI utility at T3 and T4; QALYs baseline to T4; two sickness absence counts at T3 and T4. No formal testing hierarchy; secondary endpoints are supportive |
|  | 25b | Specific measurement and units (e.g., glucose control, hbA1c [mmol/mol or %]) | Table 6. BRS mean score (1 to 5); CCAE mean score; OLBI subscale and total means; PHQ-9 total (0 to 27); ReQoL-UI utility index; QALYs from utilities; sickness absence as days lost in the past 4 weeks (two items, analysed separately) |
|  | 25c | Any calculation or transformation used to derive the outcome (e.g., change from baseline, QoL score, time to event, logarithm, etc.) | Table 6. Scored per each measure's guidance, with reverse scoring where required. Item level missingness handled by person mean substitution up to the per scale thresholds in Table 6; beyond those the score is set to missing. ReQoL-UI utilities via the published algorithm; QALYs by area under the utility time curve with linear interpolation. No transformations of the analysis scale |
| Analysis methods | 26a | What analysis method will be used and how the treatment effects will be presented | Sections 12 and 13. Primary: linear mixed model for repeated measures (REML) on BRS at T2 to T4 with baseline as covariate, reporting the adjusted Thumos minus SAU difference at T3 with 95% CI, p-value and Cohen's *d* standardised by the pooled baseline SD. Continuous secondary endpoints mirror this. Counts: Poisson GLMM with negative binomial fallback, reported as adjusted incidence rate ratios with adjusted mean days lost per arm. QALYs: linear regression adjusted for baseline utility and the primary covariate set |
|  | 26b | Any adjustment for covariates | Baseline value of the outcome, age, gender and medical school (Section 12) |
|  | 26c | Methods used for assumptions to be checked for statistical methods | Sections 10 and 12. Residual, scale-location and influence diagnostics, Q-Q plots, and inspection of estimated covariance parameters and information criteria |
|  | 26d | Details of alternative methods to be used if distributional assumptions do not hold, e.g., normality, proportional hazards, etc. | Sections 10, 12 and 13. AR(1) covariance if the unstructured model fails to converge; negative binomial if counts are overdispersed; CR2 cluster robust standard errors (clubSandwich) clustered by participant if diagnostics show clear non-normality or heteroskedasticity, with model based and robust SEs reported side by side |
|  | 26e | Any planned sensitivity analyses for each outcome where applicable | Sections 11 and 13. Multiple imputation under MAR; pattern mixture delta adjustment (0.10, 0.20, 0.30 BRS points applied to imputed values in the Thumos arm); last post-baseline carried forward; per protocol analysis; CACE |
|  | 26f | Any planned subgroup analyses for each outcome including how subgroups are defined | None pre-specified. The CACE (Table 4) targets a principal stratum rather than an observed subgroup |
| Missing data | 27 | Reporting and assumptions/statistical methods to handle missing data (e.g., multiple imputation) | Sections 9 and 11. Item level missingness by person mean substitution within thresholds (Table 6). Primary analysis uses all available data and assumes MAR given observed outcomes and model covariates. Missingness patterns and predictors described, including logistic models with missingness as the outcome. Sensitivity analyses per Table 7 |
| Additional analyses | 28 | Details of any additional statistical analyses required, e.g., complier-average causal effect analysis | Section 13 and Table 8. CACE for BRS at T3 by two stage least squares (single IV fit), with allocation instrumenting a binary take up indicator, reporting the effect among students who would take up Thumos with 95% CI, the take up proportion, and the first stage partial F statistic (below 10 flagged as a weak instrument). Assumptions stated in Table 4 |
| Harms | 29 | Sufficient detail on summarizing safety data, e.g., information on severity, expectedness, and causality; details of how adverse events are coded or categorized; how adverse event data will be analysed, i.e., grade 3/4 only, incidence case analysis, intervention emergent analysis | Table 8. Number and proportion of participants with at least one AE and at least one SAE by arm, with risk differences and 95% CIs and narrative summaries by category and seriousness. No hypothesis testing. Definitions, coding, reporting routes and stopping guidelines are in protocol sections 10 and 13.3 |
| Statistical software | 30 | Details of statistical packages to be used to carry out analyses | Table 1 and Section 15. R version 4.5.1 or the latest available at the time of analysis, using lme4, lmerTest, emmeans, mice, AER, clubSandwich and psych. All code version controlled |
| References | 31a | References to be provided for nonstandard statistical methods | Section 18 |
|  | 31b | Reference to Data Management Plan | N/A, no separate DMP; data handling is specified in protocol sections 14 and 15 |
|  | 31c | Reference to the Trial Master File and Statistical Master File | Held by the sponsor, University of Manchester |
|  | 31d | Reference to other standard operating procedures or documents to be adhered to | Sheffield CTRU SOP ST001, "The Statistical Analysis Plan" (02/07/2025) |

## **Sec 15: Example R code snippets**

```
## ACTIVATE SAP Section 15: worked examples on SIMULATED data.
## Verified on R 4.3.3, lme4 1.1-35.1, mice 3.16.0, AER 1.2-12.
## No trial data are used; no result here says anything about Thumos.

pkgs <- c("lme4", "lmerTest", "emmeans", "mice", "broom.mixed", "AER",
          "psych", "GPArotation", "clubSandwich", "tidyr", "dplyr")
new <- setdiff(pkgs, rownames(installed.packages()))
if (length(new)) install.packages(new)
invisible(lapply(pkgs, library, character.only = TRUE))

set.seed(2026)

## ---- 1. Simulate a trial-shaped dataset --------------------
n   <- 220
sim <- tibble(
  pid    = sprintf("P%03d", 1:n),
  treat  = factor(rep(c("SAU","Thumos"), each = n/2), levels = c("SAU","Thumos")),
  age    = round(rnorm(n, 23, 3)),
  gender = factor(sample(c("F","M","Other"), n, TRUE, c(.55,.42,.03))),
  school = factor(sample(paste0("S", 1:12), n, TRUE))
)

u <- rnorm(n, 0, 0.55)                                # participant intercept
brs_items <- sapply(1:6, function(i) pmin(5, pmax(1, round(2.7 + u + rnorm(n, 0, .8)))))
colnames(brs_items) <- paste0("brs_i", 1:6)
sim$brs_T1 <- rowMeans(brs_items)

eff <- ifelse(sim$treat == "Thumos", 0.35, 0)         # true effect, BRS points
brs <- function(m) pmin(5, pmax(1, 2.75 + u + m*eff + rnorm(n, 0, .45)))
sim$brs_T2 <- brs(0.8); sim$brs_T3 <- brs(1); sim$brs_T4 <- brs(0.8)

frail <- rnorm(n, 0, 0.8)                             # frailty for counts
for (t in c("T1","T2","T3","T4")) {
  sim[[paste0("util_", t)]] <- pmin(1, pmax(-.2,
    .70 + .25*(sim$brs_T1 - 2.7) +
    ifelse(t %in% c("T3","T4"), .02*(sim$treat == "Thumos"), 0) + rnorm(n, 0, .12)))
  sim[[paste0("days_", t)]] <- rpois(n, exp(-0.2 + frail -
    ifelse(sim$treat == "Thumos" & t %in% c("T3","T4"), 0.35, 0)))
}

sim$took_up <- ifelse(sim$treat == "Thumos", rbinom(n, 1, .70), 0)

d2 <- runif(n) < ifelse(sim$treat == "SAU", .12, .08) # monotone attrition
d3 <- d2 | runif(n) < ifelse(sim$treat == "SAU", .18, .13)
d4 <- d3 | runif(n) < ifelse(sim$treat == "SAU", .15, .12)
for (v in c("brs","util","days")) {
  sim[[paste0(v,"_T2")]][d2] <- NA
  sim[[paste0(v,"_T3")]][d3] <- NA
  sim[[paste0(v,"_T4")]][d4] <- NA
}

wk <- function(mu, lo, hi) pmin(hi, pmax(lo, round(rnorm(n, mu, 3))))
sim$wk_T2 <- wk(6, 4, 10); sim$wk_T3 <- wk(16, 12, 24); sim$wk_T4 <- wk(36, 30, 44)

## ---- 2. Long format and assessment windows -----------------
long <- sim %>%
  pivot_longer(matches("^(brs|util|days|wk)_T[234]$"),
```

names_to = c(".value","tp"), names_pattern = "(\\w+)_(T[234])") %>%

```
  rename(weeks = wk) %>%
  mutate(time_f = case_when(weeks >=  4 & weeks <= 10 ~ "T2",
                            weeks >= 12 & weeks <= 24 ~ "T3",
                            weeks >= 30 & weeks <= 44 ~ "T4"),
         time_f = factor(time_f, levels = c("T2","T3","T4"))) %>%
  filter(!is.na(time_f)) %>%
  select(pid, treat, age, gender, school, baseline = brs_T1, util_T1,
         took_up, time_f, weeks, brs, util, days)

## ---- 3. Primary analysis -----------------------------------
fit <- lmer(brs ~ treat*time_f + baseline + age + gender + school + (1|pid),
            data = long, REML = TRUE)

emm <- emmeans(fit, ~ treat | time_f)
ctr <- confint(pairs(emm, reverse = TRUE))
cat("\n--- adjusted means ---\n"); print(emm)
cat("\n--- Thumos minus SAU ---\n"); print(ctr)

## ---- 4. Cohen's d, pooled baseline SD ----------------------
sd_bl <- sd(sim$brs_T1, na.rm = TRUE)
d_T3  <- as.data.frame(ctr) %>% filter(time_f == "T3") %>%
  transmute(d = estimate/sd_bl, lcl = lower.CL/sd_bl, ucl = upper.CL/sd_bl)
cat("\n--- Cohen's d at T3 (SD =", round(sd_bl, 3), ") ---\n"); print(d_T3)

## ---- 5. Multiple imputation --------------------------------
wide_mi <- sim %>% select(treat, age, gender, school, took_up,
                          brs_T1, brs_T2, brs_T3, brs_T4)
imp <- mice(wide_mi, m = 10, printFlag = FALSE, seed = 2026)

# T3 is the reference level here, so the pooled treatThumos coefficient
# IS the T3 contrast (emmeans does not pool across imputations).
to_long <- function(d) {
  d$pid <- sim$pid
  d %>% pivot_longer(brs_T2:brs_T4, names_to = "time_f",
                     names_prefix = "brs_", values_to = "brs") %>%
    mutate(time_f = factor(time_f, levels = c("T3","T2","T4")))
}

mi_fit <- function(data_list)
  pool(as.mira(lapply(data_list, function(d)
    lmer(brs ~ treat*time_f + brs_T1 + age + gender + school + (1|pid),
         data = to_long(d)))))

imp_sets <- lapply(1:imp$m, function(i) complete(imp, i))
cat("\n--- MI under MAR: treatment effect at T3 ---\n")
print(summary(mi_fit(imp_sets), conf.int = TRUE) %>%
        filter(term == "treatThumos") %>%
        select(estimate, std.error, `2.5 %`, `97.5 %`))

## ---- 6. Delta adjustment -----------------------------------
was_na <- is.na(wide_mi[, c("brs_T3","brs_T4")])

# arm = "both" penalises every imputed value; arm = "Thumos" penalises the
# intervention arm only. With differential attrition these are NOT
# equivalent: see the two sets of results below.
delta_sets <- function(delta, arm = c("both","Thumos")) {
  arm  <- match.arg(arm)
  cols <- c("brs_T3","brs_T4")
  lapply(imp_sets, function(d) {
    hit <- was_na & (if (arm == "both") TRUE else d$treat == "Thumos")
    d[cols][hit] <- d[cols][hit] - delta
    d
  })
}

cat("\n--- delta adjusted T3 effects ---\n")
for (a in c("both","Thumos")) {
  cat("penalty applied to:", a, "\n")
  for (dl in c(0.10, 0.20, 0.30)) {
    r <- summary(mi_fit(delta_sets(dl, a)), conf.int = TRUE) %>%
           filter(term == "treatThumos")
    cat(sprintf("  delta %.2f:  %.3f (%.3f to %.3f)\n",
                dl, r$estimate, r$`2.5 %`, r$`97.5 %`))
  }
}

## ---- 7. Count outcome --------------------------------------
fit_p <- glmer(days ~ treat*time_f + age + gender + (1|pid),
               data = long, family = poisson(link = "log"))

disp <- sum(residuals(fit_p, type = "pearson")^2) / df.residual(fit_p)
cat("\n--- dispersion ratio ---\n"); print(round(disp, 2))

fit_cnt <- if (disp > 1.5) {              # refit only if overdispersed
  glmer.nb(days ~ treat*time_f + age + gender + (1|pid), data = long)
} else fit_p

cat("\n--- rate ratios ---\n")
print(pairs(emmeans(fit_cnt, ~ treat|time_f, type = "response"), reverse = TRUE))
cat("\n--- mean days lost per arm ---\n")
print(emmeans(fit_cnt, ~ treat|time_f, type = "response"))

## ---- 8. QALYs ----------------------------------------------
util_long <- sim %>%
  select(pid, util_T1, util_T2, util_T3, util_T4) %>%
  pivot_longer(-pid, names_to = "tp", names_prefix = "util_", values_to = "util") %>%
  mutate(weeks = c(T1 = 0, T2 = 6, T3 = 16, T4 = 36)[tp]) %>%
  arrange(pid, weeks)

qaly <- util_long %>% group_by(pid) %>%
  mutate(years = (weeks - lag(weeks))/52.18,
         area  = ((util + lag(util))/2) * years) %>%
  summarise(qaly = sum(area, na.rm = TRUE), .groups = "drop") %>%
  left_join(select(sim, pid, treat, util_T1, age, gender, school), by = "pid")

cat("\n--- QALY difference ---\n")
print(confint(lm(qaly ~ treat + util_T1 + age + gender + school,
                 data = qaly))["treatThumos", ])

## ---- 9. CACE -----------------------------------------------
iv_form <- brs ~ took_up + brs_T1 + age + gender | treat + brs_T1 + age + gender

cace <- suppressWarnings(pool(as.mira(lapply(imp_sets, function(d)
  ivreg(iv_form, data = rename(d, brs = brs_T3))))))
cat("\n--- CACE at T3 (pooled over imputations) ---\n")
print(suppressWarnings(summary(cace, conf.int = TRUE)) %>% filter(term == "took_up"))

obs <- filter(long, time_f == "T3")
cat("\n--- weak instrument test, observed cases ---\n")
print(summary(ivreg(brs ~ took_up + baseline + age + gender |
                          treat   + baseline + age + gender,
                    data = obs), diagnostics = TRUE)$diagnostics["Weak instruments", ])
cat("take up in Thumos arm:", round(mean(obs$took_up[obs$treat == "Thumos"]), 3), "\n")

## ---- 10. Reliability ---------------------------------------
items <- as.data.frame(brs_items)

rel <- function(d) {                    # alpha and omega for one dataset
  a <- suppressWarnings(suppressMessages(
    psych::alpha(d, warnings = FALSE)$total$raw_alpha))
  invisible(capture.output(o <- suppressWarnings(suppressMessages(
    psych::omega(d, nfactors = 1, plot = FALSE)))))
  c(alpha = a, omega = o$omega.tot)
}

est <- rel(items)
bs  <- replicate(1000, {                # percentile bootstrap CIs
  d <- items[sample(nrow(items), replace = TRUE), ]
  tryCatch(rel(d), error = function(e) c(alpha = NA, omega = NA))
})

cat("\n--- BRS reliability at T1 ---\n")
print(round(cbind(estimate = est,
                  t(apply(bs, 1, quantile, c(.025, .975), na.rm = TRUE))), 3))

## ---- 11. Diagnostics ---------------------------------------
cat("\n--- diagnostics ---\n")
cat("scaled residuals |r| > 3:", sum(abs(resid(fit, scaled = TRUE)) > 3), "\n")

cd <- cooks.distance(influence(fit, group = "pid"))
cat("participants above 4/n Cook's D:", sum(cd > 4/length(cd)), "\n")

## ---- 12. Robust standard errors ----------------------------
# Cluster is taken from the model frame, not the original data, because
# lmer drops rows with a missing outcome.
v  <- vcovCR(fit, cluster = fit@frame$pid, type = "CR2")
rb <- as.data.frame(coef_test(fit, vcov = v))

cmp <- data.frame(term      = rb$Coef,
                  model_se  = summary(fit)$coefficients[rb$Coef, "Std. Error"],
                  robust_se = rb$SE)
cmp$ratio <- cmp$robust_se / cmp$model_se

cat("\n--- model vs robust SE, treatment terms ---\n")
print(cmp[grepl("treat", cmp$term), ], row.names = FALSE)

## ---- 13. FDR adjustment ------------------------------------
p_T3 <- c(CCAE = .012, OLBI_tot = .048, OLBI_ex = .031, OLBI_dis = .13,
          PHQ9 = .22, ReQoL = .31, days_all = .44, days_work = .61)
cat("\n--- BH adjusted p-values, T3 family ---\n")
print(round(p.adjust(p_T3, method = "BH"), 3))
```

## **Sec 16: Version history**

**Table 10. SAP version history**

| **Version** | **Date** | **Key changes** (tracked changes documents kept + available on request) |
| --- | --- | --- |
| 0.1 | 27/08/2025 |  |
| 0.2 | 28/08/2025 | Added completed checklist |
| 0.3 | 03/09/2025 | - Modified in line with Christopher Taylor’s comments (draft with comments kept) - Modified to be more in line with previous published examples e.g. SleepWell and iMAPS-2 SAPs - Included more background, specified more detail how to handle missingness in items on measures, clarified things such as missing data approaches, multiplicity adjustment, added code snippets, and added this version history section |
| 0.4 | 25/09/2025 | Modified after further feedback from Chritopher, Judith Johnson, and Lucy Pointon:   - The handling of ‘intercurrent ‘events is narrower, with academic interruptions dropped, and external support is now only explored in sensitivity analyses rather than adjusted directly. - Support access, previously a listed secondary outcome, is no longer specified as such. - The PP/CACE compliance definition is relaxed so that only both workshops (≥75% attendance within 6 weeks) are required, whereas the one-to-one call is optional. - Missing- tem rules are slightly stricter, reverting OLBI back to ≥7 of 8 items per subscale (max 1 imputation) rather than the older ≥6 with up to 2 imputations. - The primary model covariates are trimmed (dropping “year of study”) and clustering sensitivity is simpler (therapist intercept only, without medical school effects). - Section wording is also more extensive, with greater emphasis on SOPs and guidelines. |
| 0.5 | 15/11/2025 | Modified after feedback from our independent statistician. Mainly clarifications, including:   - Converting standardised effect sizes to real units to further justify sample size analysis - Added much more detail to sample size section - Clarified the use of multilevel models - Labelled tables and provided definitions - Added more clarity and corrections around handling missing item data for outcome measures - Rewritten the missing data section for clarity - Added more detail to analyses sections - Added more citations throughout to justify some of our decisions |
| 0.6 | 16/12/2025 | Addressed the following final comments from Mona, the trial independent statistican, namely:   - Added rationale for reliability analyses - Added more clarity in missing data section |
| 1 | 13/08/2026 | - Aligned with protocol v2.3 (15/01/2026). The minimum adherence definition now matches protocol section 9.5 throughout: completion of both group workshops, operationalised as attendance at both within 6 weeks of randomisation with at least 75% of each session attended; the one-to-one call is not required. Earlier wording requiring the call, which had survived in the estimand, additional analyses and checklist sections, has been corrected. - Cohen's *d* standardiser specified. The standardiser is now stated explicitly as the pooled baseline (T1) SD of the relevant outcome, replacing the previous "model based marginal SD at T3" wording. - Support access is no longer analysed as an efficacy endpoint. It is retained, collected at all four time points, and summarised descriptively by arm and time point as context for the treatment policy strategy. It is not used as a covariate for any other outcome, because it is measured post-randomisation and may itself be affected by allocation. - Therapist clustering sensitivity analysis removed. Randomisation is at the individual level and the control arm has no equivalent grouping. The likely magnitude of any clustering is noted in Section 13 instead. - Spceified that delta adjustment now applies to the Thumos arm *only*. Applying the penalty to both arms is not conservative when attrition is differential, since the arm with more missing data is penalised more. - More details on our blinding approach. - Multiplicity wording corrected from Storey *q*-values to false discovery rate adjusted *p*-values by the Benjamini-Hochberg procedure (which is more preferable), calculated within the T3 and T4 families separately. - Estimands restructured into three tables (primary, secondary, supplementary) following ICH E9(R1) attributes. - Structural changes: a new trial method summary table replaces the synopsis; all tables numbered and cross-referenced; sections renumbered, so the reporting checklist is now Section 14; the checklist rebuilt against the Gamble et al. (2017) section structure, including the previously missing statistical principles and trial population headings; references split into background references (Section 17) and SAP method references (Section 18), with in text DOIs replaced by author-date citations. - Section 15 rewritten as a single self-contained script that runs end to end on simulated data, so every analysis specified in Sections 10 to 13 has verified syntax before the trial dataset exists. - Minor corrections to typographical errors, the version date in the header table, and cross-references throughout. |

## **Sec 17: Background references**

1.	Haakenstad, A., et al., *Measuring the availability of human resources for health and its relationship to universal health coverage for 204 countries and territories from 1990 to 2019: a systematic analysis for the Global Burden of Disease Study 2019.* The Lancet, 2022. **399**(10341): p. 2129-2154.

2.	The Health Foundation, The Kingsfund, and Nuffield Trust. *Closing the gap: Key areas for action on the health and care workforce*. 2019 [cited 2019 20th September]; Available from: <https://www.kingsfund.org.uk/sites/default/files/2019-03/closing-the-gap-health-care-workforce-overview_0.pdf>

3.	Dall'Ora, C., et al., *Nurse staffing levels and patient outcomes: a systematic review of longitudinal studies.* International Journal of Nursing Studies, 2022: p. 104311.

4.	Maslach, C. and S.E. Jackson, *The measurement of experienced burnout.* Journal of Occupational Behavior, 1981. **2**(2): p. 99-113.

5.	Toppinen-Tanner, S., et al., *Burnout as a predictor of medically certified sick-leave absences and their diagnosed causes.* Behavioral medicine, 2005. **31**(1): p. 18-32.

6.	Bae, S.H., et al., *Predictors of actual turnover among nurses working in Korean hospitals: A nationwide longitudinal survey study.* Journal of Nursing Management, 2021. **29**(7): p. 2102-2114.

7.	Hodkinson, A., et al., *Associations of physician burnout with career engagement and quality of patient care: systematic review and meta-analysis.* BMJ, 2022. **378**.

8.	Vogt, K.S., et al., *Critical care nursing workforce in crisis: A discussion paper examining contributing factors, the impact of the COVID‐19 pandemic and potential solutions.* Journal of Clinical Nursing, 2023.

9.	Johnson, J., et al., *Reboot coaching programme: a mixed-methods evaluation assessing resilience, confidence, burnout and depression in medical students.* Scottish Medical Journal, 2023: p. 00369330231213981.

10.	Rotenstein, L.S., et al., *Prevalence of depression, depressive symptoms, and suicidal ideation among medical students: a systematic review and meta-analysis.* Jama, 2016. **316**(21): p. 2214-2236.

11.	Puthran, R., et al., *Prevalence of depression amongst medical students: A meta‐analysis.* Medical education, 2016. **50**(4): p. 456-468.

12.	Dyrbye, L.N., et al., *Burnout and suicidal ideation among US medical students.* Annals of internal medicine, 2008. **149**(5): p. 334-341.

13.	British Medical Association. *Four in ten junior doctors plan to leave the NHS as soon as they can find another job, BMA council chair reveals in New Year's message*. 2022 [cited 2023 23rd June]; Available from: <https://www.bma.org.uk/bma-media-centre/four-in-ten-junior-doctors-plan-to-leave-the-nhs-as-soon-as-they-can-find-another-job-bma-council-chair-reveals-in-new-years-message-to-the-country>.

14.	Tolksdorf, K.H., U. Tischler, and K. Heinrichs, *Correlates of turnover intention among nursing staff in the COVID-19 pandemic: a systematic review.* BMC Nursing, 2022. **21**(1): p. 174.

15.	Hill, M.R., S. Goicochea, and L.J. Merlo, *In their own words: stressors facing medical students in the millennial generation.* Medical education online, 2018. **23**(1): p. 1530558.

16.	Hu, K.S., J.T. Chibnall, and S.J. Slavin, *Maladaptive perfectionism, impostorism, and cognitive distortions: Threats to the mental health of pre-clinical medical students.* Academic Psychiatry, 2019. **43**(4): p. 381-385.

17.	Weurlander, M., et al., *How do medical and nursing students experience emotional challenges during clinical placements?* International journal of medical education, 2018. **9**: p. 74.

18.	Nteveros, A., et al., *Burnout among medical students in Cyprus: A cross-sectional study.* PLoS One, 2020. **15**(11): p. e0241335.

19.	Witt, K., et al., *Effectiveness of universal programmes for the prevention of suicidal ideation, behaviour and mental ill health in medical students: a systematic review and meta-analysis.* BMJ Ment Health, 2019. **22**(2): p. 84-90.

20.	Gridley, K., *Resilience training is just a band‐aid solution for doctor well‐being: No.* 2018. **30**(2): p. 261-262.

21.	Chew‐Graham, C.A., A. Rogers, and N. Yassin, *‘I wouldn't want it on my CV or their records': medical students' experiences of help‐seeking for mental health problems.* Medical education, 2003. **37**(10): p. 873-880.

22.	Dyrbye, L.N., et al., *The impact of stigma and personal experiences on the help-seeking behaviors of medical students with burnout.* Academic medicine, 2015. **90**(7): p. 961-969.

23.	Johnson, J., Simms-Ellis, R., Janes, G., Mills, T., Budworth, B., Atkinson, L. & Harrison, R., *Can we prepare healthcare professionals for involvement in adverse events? Feasibility study of a resilience training intervention.* BMC Health Services Research, 2020. **20**: p. 1094.

24.	Janes, G., et al., *Multiple meanings of resilience: Health professionals' experiences of a dual element training intervention designed to help them prepare for coping with error.* Journal of Evaluation in Clinical Practice, 2021. **28**: p. 315-323.

25.	Vogt, K., et al., *Study protocol for the online adaptation and evaluation of the ‘Reboot’(Recovery-boosting) coaching programme, to prepare critical care nurses for, and aid recovery after, stressful clinical events.* Pilot and Feasibility Studies, 2022. **8**(1): p. 63.

26.	Vogt, K., et al., *Can the Reboot coaching programme support critical care nurses in coping with stressful clinical events? A mixed-methods evaluation assessing resilience, burnout, depression and turnover intentions.* BMC Health Services Research, 2024. **In press**

## **Sec 18: SAP references**

1. Kahan BC, Hindley J, Edwards M, Cro S, Morris TP. The estimands framework: a primer on the ICH E9(R1) addendum. *BMJ* 2024;384:e076316.
2. Johnson J, Simms-Ellis R, Grange A, et al. Reboot coaching programme: a mixed-methods evaluation assessing resilience, confidence, burnout and depression in medical students. *Scottish Medical Journal* 2023;68(4):00369330231213981.
3. Cohen J. A power primer. *Psychological Bulletin* 1992;112(1):155-159.
4. Smith BW, Dalen J, Wiggins K, Tooley E, Christopher P, Bernard J. The Brief Resilience Scale: assessing the ability to bounce back. *International Journal of Behavioral Medicine* 2008;15(3):194-200.
5. de Miquel C, Haro JM, van der Feltz-Cornelis CM, Ortiz-Tallo A, Chen T, Sinokki M, Naumanen P, Olaya B, Lima RA. Differential attrition and engagement in randomized controlled trials of occupational mental health interventions in person and online: a systematic review and meta-analysis. *Scandinavian Journal of Work, Environment & Health* 2024;50(8):588-601.
6. Keetharuth AD, Brazier J, Connell J, et al. Recovering Quality of Life (ReQoL): a new generic self-reported outcome measure for use with people experiencing mental health difficulties. *British Journal of Psychiatry* 2018;212(1):42-49.
7. Huisman M. Imputation of missing item responses: some simple techniques. *Quality & Quantity* 2000;34(4):331-351.
8. Parent MC. Handling item-level missing data: simpler is just as good. *The Counseling Psychologist* 2013;41(4):568-600.
9. Jacoby P, Whitehouse A, Leonard H, Saldaris J, Demarest S, Benke T, Downs J. Devising a missing data rule for a quality of life questionnaire: a simulation study. *Journal of Developmental & Behavioral Pediatrics* 2022;43(6):e414-e418.
10. Angrist JD, Imbens GW, Rubin DB. Identification of causal effects using instrumental variables. *Journal of the American Statistical Association* 1996;91(434):444-455.
11. Dunn G, Maracy M, Tomenson B. Estimating treatment effects from randomized clinical trials with noncompliance and loss to follow-up: the role of instrumental variable methods. *Statistical Methods in Medical Research* 2005;14(4):369-395.
12. Boatman JA, Vock DM, Koopmeiners JS, Donny EC. Estimating causal effects from a randomized clinical trial when noncompliance is measured with error. *Biostatistics* 2018;19(1):103-118.
13. Manca A, Hawkins N, Sculpher MJ. Estimating mean QALYs in trial-based cost-effectiveness analysis: the importance of controlling for baseline utility. *Health Economics* 2005;14(5):487-496.
14. Gamble C, Krishan A, Stocken D, et al. Guidelines for the content of statistical analysis plans in clinical trials. *JAMA* 2017;318(23):2337-2343.
15. Sheffield Clinical Trials Research Unit. SOP ST001: The Statistical Analysis Plan. University of Sheffield, 02/07/2025.
