# Supplementary material for: ACTIVATE: A protocol for a randomised controlled evaluation of Thumos: A cognitive-behavioural intervention for medical students
Source: PLoS One. 2026 Sep 25;21(9):e0355371. doi: 10.1371/journal.pone.0355371 (PMC13614533; doi:10.1371/journal.pone.0355371)
Supplement: Supplementary file 3 [file pone.0355371.s003.docx]

**Supporting Information**

**Appendix 1: List of eligible UK medical schools**

University of Southampton School of Medicine

Hull York Medical School (HYMS)

University of Leicester Medical School

Brighton and Sussex Medical School

University College London Medical School (UCL)

King’s College London GKT School of Medical Education (KCL)

City St George’s, University of London

University of Birmingham School of Medical Sciences

University of Dundee School of Medicine

University of Liverpool School of Medicine

University of Sheffield medical school

University of Manchester medical school

Anglia Ruskin University School of Medicine

Aston University Medical School

Brunel University Medical School

Cardiff University School of Medicine

Edge Hill University Medical School

Imperial College London Faculty of Medicine

Keele University School of Medicine

Kent and Medway Medical School

Lancaster University Medical School

London School of Hygiene and Tropical Medicine (LSHTM)

Newcastle University School of Medical Education

North Wales Medical School, Bangor University

Norwich Medical School, University of East Anglia

Pears Cumbria School of Medicine

Peninsula Medical School Plymouth University

Queen Mary, University of London School of Medicine

Queen’s University Belfast School of Medicine

St Mary’s University, Twickenham School of Medicine

University of Cambridge, School of Clinical Medicine

University of Oxford, Medical Sciences

University of Edinburgh, Medical School

University of Glasgow, School of Medicine

University of Bristol, Medical School

University of Buckingham Medical School

University of Lancashire School of Medicine

Swansea University Medical School

Three Counties Medical School, University of Worcester

Ulster University, School of Medicine

University of Aberdeen School of Medicine

University of Chester Medical School

University of Exeter Medical School

University of Greater Manchester, Medical School

University of Leeds School of Medicine

University of Lincoln Medical School

University of Nottingham School of Medicine

University of St Andrews School of Medicine

University of Sunderland School of Medicine

University of Surrey School of Medicine

University of Warwick Medical School
