# Supplementary material for: ACTIVATE: A protocol for a randomised controlled evaluation of Thumos: A cognitive-behavioural intervention for medical students
Source: PLoS One. 2026 Sep 25;21(9):e0355371. doi: 10.1371/journal.pone.0355371 (PMC13614533; doi:10.1371/journal.pone.0355371)
Supplement: Supplementary file 4 [file pone.0355371.s004.docx]

**Supporting Information**

**Appendix 2: Participant Information Sheet and Consent Form**

**V3 11-03-2025 – medical students**

**A randomised controlled trial evaluating whether a CBT-based group and one-to-one supportive intervention can improve burnout and mental health in medical students**

**Participant Information Sheet (PIS) for medical students**

You are being invited to take part in a research study evaluating an intervention in medical students. Before you decide whether to take part, it is important for you to understand why the research is being conducted and what it will involve. Please take time to read the following information carefully before deciding whether to take part and discuss it with others if you wish. Please ask if there is anything that is not clear or if you would like more information. Thank you for taking the time to read this.

**About the research**

**Who will conduct the research?**

Dr Judith Johnson and Lucy Pointon who are based in the Division of Nursing, Midwifery and Social Work at The University of Manchester. A researcher from the University of Sheffield is also participating.

**What is the purpose of the research?**

The study aims to evaluate a supportive programme (Thumos) designed to prepare medical students for stressful events faced at work.

**Am I suitable to take part?**

We’d love to hear from you if you are a medical student in a year involving clinical placements. This is usually year 4 or year 5 for most medical schools but can vary.

**Will the outcomes of the research be published?**

We will publish our findings in peer-reviewed journal articles. We might also promote the findings via social media.

**Who has reviewed the research project?**

The project has been reviewed by The University of Manchester Research Ethics Committee.

**Who is funding the research project?**

The Medical Protection Society Foundation.

**What would I be asked to do if I took part?**

If you decide to take part in the research study, you will be asked to complete a consent form and some baseline questionnaires, which will ask for demographic details (e.g., age, gender) and your thoughts and perceptions relating to your mental health and psychological wellbeing. They will take about 20m altogether. You will then be randomised to either receive the Thumos intervention, or to the control group, where you will not receive the intervention. Half of participants will be randomly allocated to receive the intervention; the other half will not be allocated to receive the intervention. This decision is completely random and is not affected by any of your responses to the questionnaires. In either group, you can continue to access your usual wellbeing or mental health support services as usual.

If you are randomly allocated to receive the intervention, we will ask you to:

- Engage in the Thumos programme, combining 2 small group online workshops and a personalised coaching call. The workshops will each last up to 2 hours and the coaching call will last up to an hour (5 hours total).
- Complete further online questionnaires after Thumos has finished, first on completion of the coaching call, then 4 months after starting the study and finally 9 months after starting the study. This will take around 20m at each time.
- Over the course of the study, some workshops will be observed by a researcher who will join the call, and we will request permission to video record some of the coaching calls. This is so that we can observe the therapist and identify how closely they are following the intervention plan. Any recordings will be destroyed once they have been watched by the research team and scored for how closely they are following the intervention plan.
- We will also ask your permission to retain your contact details, so we could contact you again in 2 and 5 years’ time, to ask you to complete further questionnaires.
- A subgroup of participants will be asked to answer additional questions as part of a qualitative interview at around 4 months after the first questionnaire, discussing your experiences of Thumos. These will last up to 30 minutes. These interviews will be conducted on Teams and video recorded.

If you are randomly allocated not to receive the intervention, we will ask you to:

- Complete further online questionnaires 4-6 weeks after the first questionnaire, again 4 months after starting the study and then finally 9 months after starting the study. This will take around 20m at each time.
- We will also ask your permission to retain your contact details, so we could contact you again in 2 and 5 years’ time, to ask you to complete further questionnaires.

**Will I be compensated for taking part?**

You will not be compensated for completing the first set of questionnaires. However, for each of the remaining three follow-up questionnaires, you will receive a £10 shopping voucher. For participants who take part in the qualitative interviews, you will receive a further £10 shopping voucher.

**What happens if I do not want to take part or if I change my mind?**

It is up to you to decide whether or not to take part. If you do decide to take part you will be given this information sheet to keep and will be asked to provide your name and tick a box to confirm consent on Qualtrics survey software online. If you decide to take part you are still free to withdraw at any time without giving a reason and without detriment to yourself. However, it will not be possible to remove your data from the project once it has been anonymised as we will not be able to identify your specific data. This does not affect your data protection rights. If you decide not to take part you do not need to do anything further.

For participants who are invited to take part in the qualitative interviews, these will be conducted on Teams and will be video recorded. We need this recording to be able to analyse the data, so we need to do this for you to take part in the interview. If should be comfortable with the recording process at all times and are free to stop recording at any time, should you feel uncomfortable.

**Data Protection and Confidentiality**

**What information will you collect about me?**

In order to participate in this research project we will need to collect information that could identify you, called “personal identifiable information”. Specifically we will need to collect:

- Your name
- Contact details and address (for posting the paper workbook to you)
- Gender
- Ethnicity
- Age
- Region
- Your year of medical school
- Whether you are in a year involving clinical placements
- Whether you have begun or completed one or more clinical placements
- Which medical school you attend
- For participants who take part in the qualitative interviews, these will be conducted on Teams. We will ask permission to video record the discussion.

**Under what legal basis are you collecting this information?**

We are collecting and storing this personal identifiable information in accordance with UK data protection law which protect your rights. These state that we must have a legal basis (specific reason) for collecting your data. For this study, the specific reason is that it is “a public interest task” and “a process necessary for research purposes”.

**What are my rights in relation to the information you will collect about me?**

You have a number of rights under data protection law regarding your personal information. For example you can request a copy of the information we hold about you, including video recordings.

If you would like to know more about your different rights or the way we use your personal information to ensure we follow the law, please consult our [Privacy Notice for Research](http://documents.manchester.ac.uk/display.aspx?DocID=37095).

**Will my participation in the study be confidential and my personal identifiable information be protected?**

In accordance with data protection law, The University of Manchester is the Data Controller for this project. This means that we are responsible for making sure your personal information is kept secure, confidential and used only in the way you have been told it will be used. All researchers are trained with this in mind, and your data will be looked after in the following way:

- For the questionnaire data, we have taken measures to ensure confidentiality, such as providing participants with an assigned ID number only known to the research team (known as pseudonymised).
- We will ask your permission to retain your data for up to 5 years after the end of the study. This is to enable us to contact you again in 2 and 5 years to conduct further follow up studies, should we gain additional funding for this. If you decline to provide permission, your identifiable data will be deleted at the end of the study, which will be 2 months after the final participant has provided their follow-up data. You can indicate your choice on the consent form. If you provide consent for this, your details will be safely stored on UoM servers in a digital folder only accessible to the study team and used only for the purposes described above.
- Data will be held on secure University of Manchester OneDrive servers. Only completely anonymised data will be shared with any other organisations (e.g., anonymous quantitative data files; anonymised transcripts, where all identifying details have been changed). This data will be shared for the purposes of statistical analysis.
- Identifiable and anonymised data will be stored for up to 5 years on University of Manchester secure OneDrive servers.
- At the end of the project we will deposit a fully anonymised dataset [e.g. including de-identified interview transcripts] in an open data repository where it will be permanently stored. We will use the University of Manchester Library. Researchers at other institutions and others can access the anonymised data directly from the repository and use it for further research or to check our analysis and results.
- For participants who take part in online qualitative interviews, the recordings will be used to create transcripts. This will be done initially by the Teams transcription software and then will be checked and corrected by a study team researcher.
  - Personal identifiable information will be removed in the final transcript
  - The recordings will be destroyed following transcription
  - Your participation in the qualitative interview will be recorded in Teams and your personal data will be processed by Microsoft. This may mean that your personal data is transferred to a country outside of the European Economic Area, some of which have not yet been determined by the United Kingdom to have an adequate level of data protection. Appropriate legal mechanisms to ensure these transfers are compliant with the Data Protection Act 2018 and the UK General Data Protection Regulation are in place. The recordings will be removed from the above third party platform and stored on University of Manchester managed file storage as soon as possible following the completion of data collection.

The study team at The University of Manchester will have access to your personal information and they will anonymise it as soon as possible. Your name and any other identifying information will be removed and replaced with a random ID number. The research team will have access to the key that links this ID number to your personal information. Your consent form will be retained for 5 years on secure University of Manchester OneDrive servers for audit purposes.

So that we can provide the shopping/Amazon voucher as a thank you for your time, your full name and email address will be shared with our Finance department who will send the voucher to you. Your full name and email address will be securely retained by Finance for a period of up to 7 years for audit purposes only and then destroyed. It will not be used for them for any other purpose.

If, during the study, you disclose information about patient safety hazards or risks you are facing on placement, we will advise you that you have a responsibility to raise these issues via your clinical supervisor and we will support you to do this. If you refuse to raise issues of concern via the normal routes, and it is likely that disclosure will prevent harm to others in future*,* we may need to contact the relevant NHS trust directly and communicate the risks/concerns directly to the trust’s local patient safety team on your behalf. We will maintain confidentiality wherever possible but break confidentiality and disclose your identity if necessary.

If, during the study, you disclose that you have experienced a serious negative event while participating in the study, we will contact you to request more information about the event, to try and understand if it is related to the study procedures.

Please also note that individuals from The University of Manchester or regulatory authorities may need to look at the data collected for this study to make sure the project is being carried out as planned. This may involve looking at identifiable data. All individuals involved in auditing and monitoring the study will have a strict duty of confidentiality to you as a research participant.

**What if I have a complaint?**

**Contact details for complaints**

If you have a complaint that you wish to direct to members of the research team, please contact: **DR JUDITH JOHNSON,** [**JUDITH.JOHNSON@MANCHESTER.AC.UK**](mailto:JUDITH.JOHNSON@MANCHESTER.AC.UK)**.**

**If you wish to make a formal complaint to someone independent of the research team or if you are not satisfied with the response you have gained from the researchers in the first instance, then please contact:**

The Research Ethics Manager, Research Office, Christie Building, The University of Manchester, Oxford Road, Manchester, M13 9PL, by emailing: [research.complaints@manchester.ac.uk](mailto:research.complaints@manchester.ac.uk)  or by telephoning 0161 306 8089.

If you wish to contact us about your data protection rights, please email [dataprotection@manchester.ac.uk](mailto:dataprotection@manchester.ac.uk) or write to The Information Governance Office, Christie Building, The University of Manchester, Oxford Road, M13 9PL at the University and we will guide you through the process of exercising your rights.

You also have a right to complain to the [Information Commissioner’s Office](https://ico.org.uk/concerns) about complaints relating to your personal identifiable information Tel 0303 123 1113

**Contact Details**

If you have any queries about the study or if you are interested in taking part, then please contact the researchers:

Lucy Pointon: [lucy.pointon@manchester.ac.uk](mailto:lucy.pointon@manchester.ac.uk) or [ThumosTrial@manchester.ac.uk](mailto:ThumosTrial@manchester.ac.uk)

Dr Judith Johnson: [Judith.johnson@manchester.ac.uk](mailto:Judith.johnson@manchester.ac.uk)

**V3 11-03-2025 – medical students**

**A randomised controlled trial evaluating whether a CBT-based group and one-to-one supportive intervention can improve burnout and mental health in medical students**

**Consent Form for medical students**

If you are happy to participate please tick each box and then enter your name below.

|  | **Activities** | Tick to indicate agreement |
| --- | --- | --- |
| 1 | I confirm that I have read the attached information sheet (**Version 3, Date 11-03-2025**) for the above study and have had the opportunity to consider the information and ask questions and had these answered satisfactorily. |  |
| 2 | I understand that my participation in the study is voluntary and that I am free to withdraw at any time without giving a reason and without detriment to myself. I understand that it will not be possible to remove my data from the project once it has been anonymised and forms part of the data set.  I agree to take part on this basis. |  |
| 3 | If I am invited to a qualitative interview and choose to take part in this, I agree to the **interviews** being **video recorded** |  |
| 4 | I agree that any data collected may be included in anonymous form in **publications/conference presentations.** |  |
| 5 | I agree that any research publications can include direct quotes of my responses in **anonymous** format. |  |
| 6 | I understand that a fully anonymised dataset will be deposited in an open data repository at the end of the project. |  |
| 7 | I understand that data collected during the study may be looked at by individuals from The University of Manchester or regulatory authorities, where it is relevant to my taking part in this research. I give permission for these individuals to have access to my data. |  |
| 8 | I understand that my full name and email address will be passed to the University's Finance team for the sole purpose of sending me the **shopping/Amazon voucher.** |  |
| 9 | I understand that there may be instances where during the course of the research information is revealed which means the researchers will be obliged to break confidentiality and this has been explained in more detail in the information sheet. |  |
| 10 | I understand that if I disclose evidence of patient safety risks in my clinical placement, I will be encouraged to report these to the relevant authorities. If I choose not to and it is likely that disclosure will prevent harm to others in future, I understand that the research team may need to raise these risks with the relevant NHS Trust, and that they will make every effort to maintain my confidentiality, but may need to disclose this and will do so if necessary. |  |
| 11 | I understand that some workshops may be observed by a researcher and that my therapist may request permission to video record our coaching call. I understand that this is for the purpose of understanding how closely the therapist is following the intervention protocol, and provide my permission for these observations/recordings. |  |
| 12 | I understand that if I disclose that a serious negative event has occurred to be during the course of the study, the research team will contact me to request more information about this. |  |
| 13 | I agree to take part in this study. |  |

**The following activities are optional, you may participate in the research without agreeing to the following:**

| 14 | I give permission for my contact details to be retained for up to 5 years to enable potential follow up studies to be conducted, regarding the intervention. I agree that the researchers may contact me in future about these projects, but do not give permission to be contacted about any other research projects. |  |
| --- | --- | --- |
| 15 | I agree that the researchers may retain my contact details in order to provide me with a summary of the findings for this study. |  |

**Data Protection**

**The personal information we collect and use to conduct this research will be processed in accordance with UK data protection law as explained in the Participant Information Sheet and the** [**Privacy Notice for Research Participants**](http://documents.manchester.ac.uk/display.aspx?DocID=37095)**.**

By providing my full name and email address below, I declare that I have provided accurate identity details.

________________________	________________________

Name of Participant	Email address	Date

This consent form will be retained on the Qualtrics platform. You can request a copy by contacting the research team (Dr Judith Johnson, [Judith.johnson@manchester.ac.uk](mailto:Judith.johnson@manchester.ac.uk); Lucy Pointon, [lucy.pointon@manchester.ac.uk](mailto:lucy.pointon@manchester.ac.uk))
